# What plant is that? Tests of automated image recognition apps for plant identification on plants from the British flora

**DOI:** 10.1093/aobpla/plaa052

**Published:** 2020-09-23

**Authors:** Hamlyn G Jones

**Affiliations:** 1 Division of Plant Sciences, School of Life Sciences, University of Dundee at the James Hutton Institute, Invergowrie, Dundee, UK; 2 School of Agriculture and Environment, University of Western Australia, Perth, WA, Australia

**Keywords:** Apps, artificial intelligence, image recognition, plant identification, smartphones

## Abstract

There has been a recent explosion in development of image recognition technology and its application to automated plant identification, so it is timely to consider its potential for field botany. Nine free apps or websites for automated plant identification and suitable for use on mobile phones or tablet computers in the field were tested on a disparate set of 38 images of plants or parts of plants chosen from the higher plant flora of Britain and Ireland. There were large differences in performance with the best apps identifying >50 % of samples tested to genus or better. Although the accuracy is good for some of the top-rated apps, for any quantitative biodiversity study or for ecological surveys, there remains a need for validation by experts or against conventional floras. Nevertheless, the better-performing apps should be of great value to beginners and amateurs and may usefully stimulate interest in plant identification and nature. Potential uses of automated image recognition plant identification apps are discussed and recommendations made for their future use.

## Teaser

There is much interest in potential uses of artificial intelligence approaches to the automated identification of unknown plants. This paper reviews and compares a number of free smartphone apps that attempt automatically to identify unknown plants from images taken in natural environments, using images of plants growing wild in Britain. These apps are continually improving but the best ones already show an outstanding success rate identifying up to three quarters of samples to family and half to species. Criteria for the selection of an app for any situation are discussed and highlight the importance of minimizing erroneous identifications.

## Introduction

Research into biodiversity and conservation requires species identification, but the availability of botanists with good plant identification skills is declining. It has therefore been suggested that artificial intelligence (AI) might replace such human expertise (Wäldchen *et al.* 2018; August *et al.* 2019), thus mitigating this ‘taxonomic gap’ (Joly *et al.* 2014). Species identification is also of interest to a more general audience including nature lovers, hikers and eco-tourists. There has been rapid recent development of smartphone apps to aid plant identification in the field, ranging from the use of those based on automated image recognition or AI (the subject of this paper), to those that require the user to use traditional dichotomous keys or multi-access keys and those that only provide a selection of images without a clear system for species identification.

There are at least three major challenges facing all automated systems. One is posed by the variability in view and quality of images taken by users in the field. A second challenge is that the characters distinguishing between species are often cryptic either being microscopic or requiring very specific views that may not be present in the images available. Any viable system needs to address both these problems. The third difficulty, particularly relevant for conservation studies, is posed by the rarity of some species which means that they may be absent (or poorly represented) in any reference set of images used for identification.

In spite of these challenges, there has been much progress on the development of image recognition/AI approaches to plant identification over the past 20 years with many hundred academic papers addressing various aspects of this problem (see e.g. Wäldchen and Mäder 2018). Many of the earlier studies of automated identification focussed on images of single organs, especially leaves, imaged on plain backgrounds (Wang *et al.* 2003; Agarwal *et al.* 2006; Valliammal and Geethalakshmi 2011; Hati and Sajeevan 2013; Satti *et al.* 2013; Shayan and Sajeevan 2013; Hsaio *et al.* 2014; Zhao *et al.* 2015; Lee *et al.* 2017; Wäldchen and Mäder 2018). Much effort has been centred on the Cross Language Evaluation Forum (CLEF) initiative (http://www.clef-initiative.eu/association), which since 2013 included the LifeCLEF challenge to develop automated identification systems. The sub-project PlantCLEF focussed on plant identification (Goëau *et al.* 2013; Cappellato *et al.* 2017), with eight different research groups contributing their models to LifeCLEF2017 (Goëau *et al.* 2017) all aiming to compare the performance of ‘experts’ with that of the best deep learning algorithms (Bonnet *et al.* 2018). Increasing the size of the database of images which can be used for identification has been one key to the rapid recent advances in the field. Leafsnap (Kumar *et al.* 2012), for example, was an early automated app for species identification using a large data base of leaf images and provides the basis for PlantSnap evaluated here. Other work has confirmed that deep neural networks can be extraordinarily powerful at detailed recognition even using noisy data (Krause *et al.* 2016) and this approach is widely used in the current apps as outlined in many recent papers and reviews that outline progress with development of image recognition for plant identification (Mata-Montero and Carranza-Rojas 2016; Cappellato *et al.* 2017; Nguyen *et al.* 2018; Wäldchen and Mäder 2018; Wäldchen *et al.* 2018; Rzanny *et al.* 2019). Image recognition technology is now maturing so rapidly that there are now a number of automatic plant identification apps available for smartphones and tablets, so it is timely to consider the state of the art in this technology. In this article I investigate the consistency and accuracy of a number of free automated image recognition apps for plant identification using a small subset of plant images from the British flora as an example (Table 1) and discuss their potential use by amateur or professional botanists.

**Table 1.** Description of the apps tested in this study (Beware: there are many other apps with very similar names). All apps were only tested in Camera mode, even where uploading is possible. The additional Android app, Plant identifier by Rakata Tech (https://play.google.com/store/apps/details?id=rakta.plant.identification&hl=en_GB), was also tested but discarded early because it failed with almost all samples.

App	Operating system	Comments	Expert/community id	Camera	
Bing https://www.bing.com/	Android/iOS/web	Rather slow; confidence hierarchy: ‘Looks like/Could be/Related images’	No	Camera/upload	
Candide (Plant ID) v.1.15.0 https://candidegardening.com/	Android/iOS	Targets genus level accuracy; confidence hierarchy: ‘List of answers/Could also be/Couldn’t find close match/’	Community	Camera/upload (but photos of screen subject to Moiré patterning)	
Flora Incognita^a^ https://floraincognita.com/	Android/iOS	User grouping into class needed; confidence hierarchy: % similarity, or ‘not identified with satisfactory accuracy’; sometimes needs two images; limited number of answers	Expert	Camera/upload (adjustable picture; can use several photos)	Location: yes
Google Lens	Android/iOS/web	Quick; confidence hierarchy: ‘Identification/Related results/Related content/Similar images’	No	Camera	
iPlant Plant identifier v.1.0.0	iOS	Generally one answer only; no confidence level given	No	Camera (can adjust photo)	
Plant.id https://plant.id/	Web (five analyses per week)	Can use several photos; confidence level: gives a % confidence; little control over photo; API available for advanced users	Expert (but only via Flowerchecker (paid)) Can feedback	Camera/upload (can use several photos)	Can use location
PlantNet https://identify.plantnet.org/	Android/iOS/web	Some user input required; has different data sets for different parts of the world; trims photos oddly, can use several photos: gives confidence score (0–5); can feedback; API available for advanced users	Can feedback	Camera/upload (can use several photos)	
PlantSnap^b^ https://www.plantsnap.com/	Android/iOS (10 samples per day—premium version available)	Possible expert id; can remove adverts in premium version; confidence hierarchy: ‘Identified/then offers more options’	Expert can feedback	Camera/upload (can trim photo)	Often suggests non-British spp.
Seek (iNaturalist) https://www.inaturalist.org/pages/seek_app	Android/iOS	Used in live mode only; appears to perform worse on uploaded photos; sometimes takes time to settle then may change; works offline	Can add to iNaturalist database	Camera/upload (appears to perform worse on uploaded photos)	Uses location to filter species, but can operate offline

^a^ Rzanny *et al.* (2019).

^b^ Kumar *et al.* (2012).

## Materials and Methods

The 10 apps studied are listed in Table 1, though one was discarded early on as its accuracy in tests was inadequate. All apps aim automatically to identify plants from images taken using smartphones in the field, and are available for download for free onto hand-held devices or run online (though two allow only a limited number of analyses per day or week). Initial tests compared the performance of the different apps either when using real plants outdoors in the field or using photographs taken of those same plants and subsequently displayed on a computer screen and reimaged by the smartphone; the results showed no consistent variation in performance, so all further results are based solely on the performance with stored images. These stored images were displayed on the screen of a Macbook Pro 13″ and viewed using a smartphone (OnePlus A5000 with Android 9) or an iPad Mini (for Plant Identifier); replicates were repeated hand-held views of the same original image. All analyses were conducted during November and December 2019; note that several of the apps tested are continually developing as user feedback occurs, though most apps do not make it clear exactly how or when that may occur.

**Figure 1.**
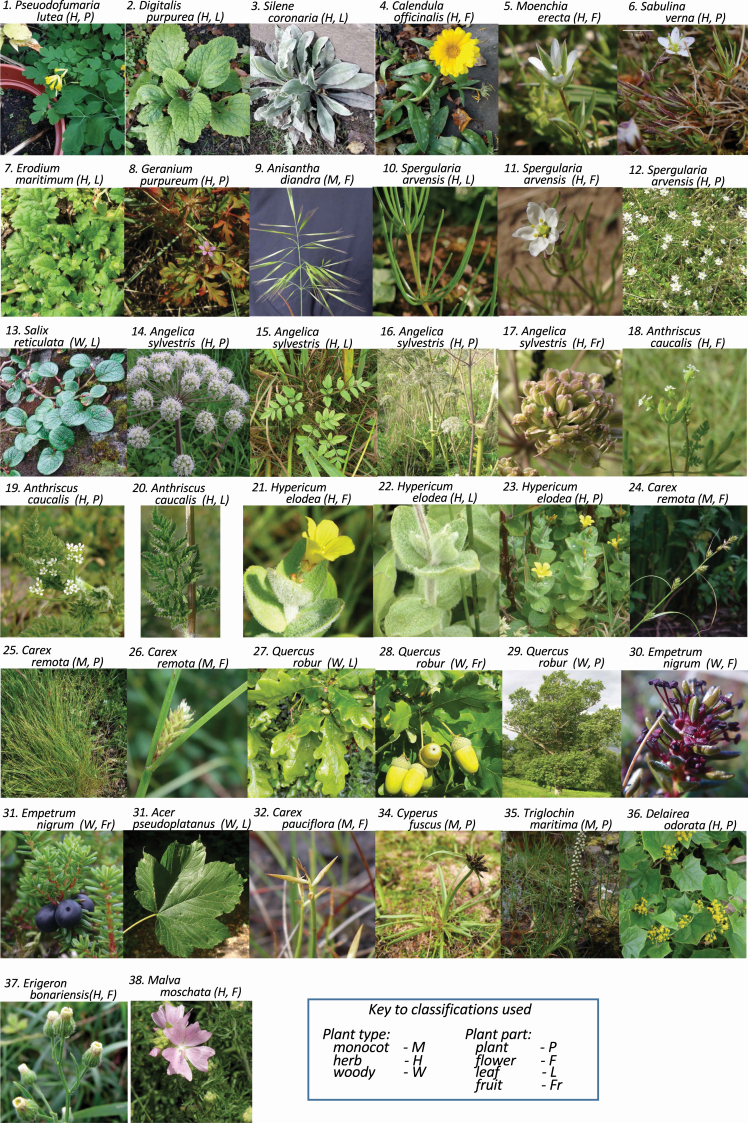
The images used for the current tests.

For this study I subjectively chose a range of images of wild and naturalized British species from a range of plant groups and families of angiosperms, identified according to Stace (2019), that were as contrasting as possible. The chosen images included ones I expected to be either easy or challenging and included leaves, flowers, fruits or whole plants of varying degrees of clarity/complexity (see Fig. 1). Five independent identifications were run with each app on each of the 38 sample images. The performance of each app was evaluated either across the whole data set (190 identifications for each app = 5 reps × 38 samples), or separately for each of the 24 species, or separately for each class of plant (monocotyledonous, herbaceous, woody) or for each part of plant imaged (leaf, flower, fruit, whole plant).

The raw identification data produced by the nine apps are presented in **Supporting Information—Table S1**. Apps were assessed using a range of possible scoring systems. The main approach used attempts to balance these various objectives in one number and is described in Box 1. Alternatively, the score for only the first suggestion was used: this score avoids bias against apps which only produce one suggestion, such as Seek. Another, more conventional, measure of accuracy was simply to count the percentage of samples that were correct to species, to genus or to family (as used for example in LifeCLEF 2017, Bonnet *et al.* 2018). As important for users is the frequency of incorrect or misleading identifications; this was measured either as the percentage of tests that were misleadingly wrong (=%mad; scored as % < 0; **see**  **Supporting Information—Table S1**), or as the percentage of tests that gave the wrong family or worse (=%wrong, i.e. ignoring cases where no id was returned). Slight subjective adjustments to the strict scoring were permitted to allow for identifications whose precision did not quite fit the defined categories **[see**  **Supporting Information—Table S1****]**, with the delineation between scores of 0 and −5 being particularly subjective. Alternative scientific names were accepted where these were given and English names were also accepted.

**Box 1 Scoring systems**

Three systems were used to assess performance of the different apps. (i) First choice: for this method only the first suggested identification was used, scored according to the weighting in column 1 of the table below. (ii) Weighted score: this score took account of up to the first four choices of identification with the second suggestion increasing any existing score by 50 % of the difference between the previous score and what it would be if first choice, the third as 25 % and the fourth as 13 %. (iii) Inconsistency (variability): this is scored as the number of Families ‘identified’ among the five replicates (with each ‘irrelevant’ being counted as a separate one), 0.2 was added to the score for each different species or genus within a family suggested.

**Table:** 

	First choice	Second choice	Third choice
Correct to species	100	50 % of upward difference from previous score to what it would be if first choice	25 % of upward difference from previous score to what it would be if first choice
Very close/indistinguishable	95		
Small genus/similar species	90		
Correct genus	80		
Similar genus	70		
Correct family	50		
Similar family	40		
Good try (similar looking plant)	10–20		
Unknown/very vaguely similar	0		
Totally wrong/misleading	−5	−4	−2

Another feature of interest to users is the consistency of identification for repeated evaluations of the same original image. For this I propose a measure that gives information about the underlying nature of the variation. To calculate this, first a measure of inconsistency (***I***: a number between 1 and 5) was determined by counting the number of families ‘identified’ among the five replicates (with each ‘irrelevant’ being counted as a separate one), where different species or genera were given these added 0.2 to the score. A consistency score, ***C***, was then calculated as 5 − ***I***, giving a number between 0 and 4. Inevitably there is a close negative association between this score and the percentage of correct identifications, so any trends need to be noted with care.

As the scoring systems used led to results that deviate strongly from normality, only non-parametric statistical tests could be used. Differences between the performance of the apps were therefore assessed either using a Kruskal–Wallis test (Sokal and Rohlf 2012) or, where proportions were available (Table 4), an (*n* − 1) Chi-square test for proportions (Campbell 2007), both used a Bonferroni correction for multiple comparisons. Tests for differences between results for different plant parts or for different plant types were also tested using the Kruskal–Wallis procedure. The consistency of ranking of the apps across subsets was tested using Kendall’s coefficient of concordance (Kendall and Gibbons 1990). These procedures are rather conservative. Calculations were performed in SPSS Statistics, version 22 from IBM. Because of the very subjective nature of the selection process for test images, and the small numbers of images in each category, any comparisons between different classes of sample (e.g. between plant parts, or between plant types) can only be taken as indicative and cannot be extrapolated to other data sets.

## Results and Discussion

### Identification success

The choice of scoring system is somewhat subjective and depends on the objectives of the user (whether interested in the first suggestion only; whether interested in identification to species, genus or family; or whether the number of errors is important). Whichever system was used, it is clear that some apps performed substantially better than others, whether classified in terms of the first suggestion only (Tables 2 and 4) or the overall score (Table 3). The average scores for the first return for the different apps varied between 13.4 and 69.8 with the top five apps (PlantNet, Flora Incognita, Seek, Google Lens and Plant.id) all with an average score greater than 50 (Table 2) and significantly (*P* < 0.05) outperforming Candide, Bing and iPlant. Results using the overall score (Table 3) led to slightly enhanced identification scores (ranging from 13.4 to 75.1). There was no evidence for any different ranking of the apps for different plant groups (e.g. monocots, herbaceous plants or woody plants (*P* = 0.164)) or for different plant parts (e.g. flowers or leaves (*P* = 0.891)), though the limited numbers of images used restrict the power of such comparisons. Further evidence for consistent ranking of the apps’ performance across the different subclasses was the highly significant Kendall’s coefficient of concordance (*W* = 0.88, *P* < 0.001). To put these scores in context, an average score of 50 or more implies a result somewhere between half of all observations being correct to the species level, or all observations being correct to family.

**Table 2.** Average scores obtained for the first identification by each of the nine apps for each subgrouping of the samples, whether by plant type (monocot, herb or woody) or by plant part (flower, fruit, plant or leaf), together with the number of samples in each subset. The rankings of the apps for each subset separately are also shown in parentheses. Grand totals indicated by a common letter are not significantly different at the 5 % level of significance (Kruskal–Wallis test with Bonferroni correction; *N* = 342). Neither of the comparisons between parts of the plant or between types of plant approached significance by the same test.

	Flower	Fruit	Leaf	Plant	Herb	Monocot	Woody	Average	
Plant.id	76 (1)	67 (4)	62 (1)	71 (1)	65 (1)	66 (2)	92 (1)	69.8	a
Google Lens	69 (2)	70 (3)	58 (2)	61 (3)	56 (3)	70 (1)	83 (2)	63.4	ab
Seek	56 (5)	79 (1)	51 (3)	68 (2)	63 (2)	51 (4)	63 (4)	60.7	ab
Flora Incognita	67 (3)	33 (6)	48 (4)	56 (4)	52 (4)	61 (3)	61 (5)	60.3	ab
PlantNet	58 (4)	73 (2)	42 (5)	50 (5)	49 (5)	50 (5)	65 (3)	52.1	ab
PlantSnap	42 (6)	47 (5)	31 (6)	43 (6)	38 (6)	31 (7)	53 (6)	39.7	bc
Candide	29 (7)	1 (9)	19 (7)	29 (7)	23 (7)	37 (6)	17 (8)	24.3	c
Bing	17 (8)	18 (8)	8 (9)	22 (8)	19 (8)	12 (9)	12 (9)	16.3	c
iPlant	12 (9)	26 (7)	11 (8)	14 (9)	7 (9)	18 (8)	30 (7)	13.4	c
Average	47.2	46.3	36.6	46.0	41.3	43.9	53.0	44.4	
No. of samples	12	3	10	13	24	24	7	38	

**Table 3.**
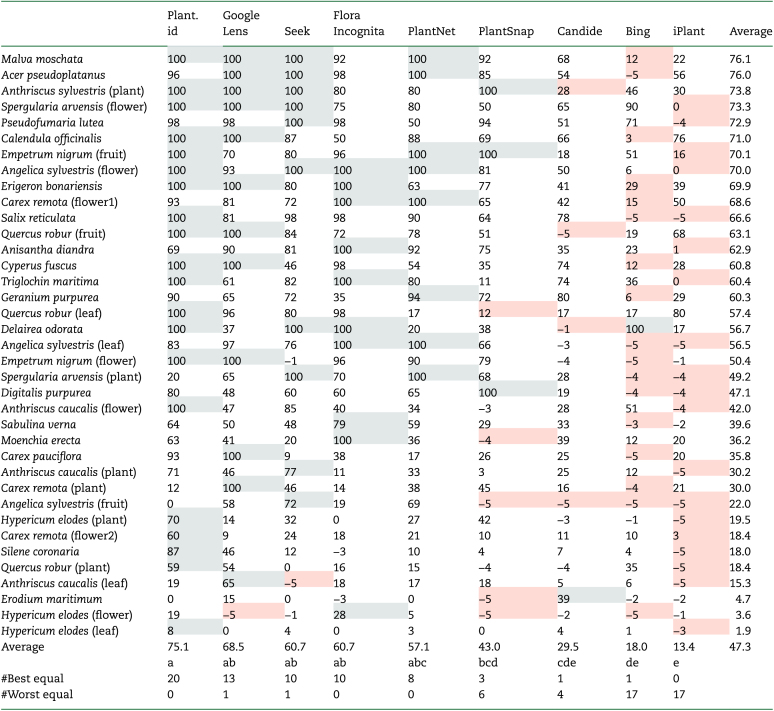
Full-weighted scores by sample, ordered by the average score across all apps. The best apps for each sample are highlighted in gray, while the worst apps for each sample are highlighted in orange; the number of times each app was best (or best equal) and worst (or worst equal) is summarized below the table. Means indicated by a common letter are not significantly different at the 5 % level of significance (Kruskal–Wallace test with Bonferroni correction; *N* = 342). A Kruskal–Wallis test also confirms that the sample averages are not equal (*P* < 0.001).

**Table 4.** Percentage of all observations achieving different levels of accuracy for the first identification returned. The column headed %mad gives the percentage of totally misleading identifications scored at <0. The final column shows the percentage of tests that gave an incorrect family (or worse) (i.e. omitting any where no id was suggested). Means within any column followed by a common letter are not significantly different at the 5 % level of significance according to a (*n* − 1) Chi-square test for proportions (with Bonferroni correction).

	% = 100	% ≥ 80	% ≥ 50	%mad	%wrong
Plant.id	57a	70a	73a	3a	27bc
Flora Incognita	46ab	56ab	64abc	7ab	14ab
Google Lens	45ab	56ab	71ab	8ab	28cd
Seek	35ab	55abc	68ab	4a	12a
PlantNet	39bc	49bc	55bc	11b	43de
PlantSnap	24c	38c	47cd	32cd	54ef
Candide	1d	18d	35de	25c	61f
Bing	3d	17d	23e	57e	67f
iPlant (Plant Identifier)	1d	8d	24e	40d	60f
Average	27.7	40.8	51.1	20.8	40.7

As expected, there were large differences among the samples in the success with which they could be identified by the apps tested (Table 3), though it was rarely obvious in advance (to me) which samples would be identified successfully. There was no obvious relationship either to rarity of the species, or to clarity of the image. The full-weighted scores for each sample averaged across all apps ranged from <5.0 to >75 (Table 3; **see**  **Supporting Information—Table S1**). The percentages of all first identifications (190 for each app) that were accurate to species, genus and family, respectively, are shown in Table 4. Impressively, for the four top apps, over 50 % of observations were correct to at least Genus.

### Error rate, confidence and consistency

Table 3 also shows two measures of the failure rate (%mad and %wrong). The best four apps all made significantly (*P* < 0.05) fewer totally misleading or ‘mad’ suggestions than the bottom four apps. Seek and Flora Incognita stood out as the most conservative apps making the fewest wrong suggestions as they tended not to give an answer where there is uncertainty. Those apps where substantial numbers of observations (>50 %) were seriously wrong would be likely to be potentially misleading especially for beginners.

The reliability or consistency of identification is another measure of interest to the user. Unsurprisingly the variability was negatively associated with identification score, because consistency is perfect (score = 4.0) where the average identification score across the five reps is 100. It is notable, however, that on this criterion Seek, along with Plant.id and Flora Incognita were significantly more consistent than the bottom five apps (Table 5). The frequency with which the five replicate results were very different is somewhat surprising, given that the five replicates were all images of the same sample photograph. This effect occurred to a greater or lesser degree with all the apps, but was worst in Candide **[see**  **Supporting Information—Table S1****]** and may have related to the Moiré patterning discussed below.

**Table 5.** The average consistency, ***C***, for each app across all samples within each class of analysis is shown, together with the ranking for each app in parentheses. Means indicated by a common letter are not significantly different at the 5 % level of significance (Kruskal–Wallis test with Bonferroni correction; *N* =332). Neither of the comparisons between parts of the plant or between types of plant approached significance by the same test.

	Flower	Fruit	Leaf	Plant	Herb	Monocot	Woody	Average
Seek	3.3 (3)	3.5 (1)	3.4 (1)	3.5 (1)	3.6 (1)	2.6 (4)	3.6 (2)	3.4a
Plant.id	3.4 (2)	3.3 (2)	3.2 (2)	2.9 (3)	3.0 (2)	3.3(2)	3.7 (1)	3.2a
Flora Incognita	3.5 (1)	2.3 (6)	2.8 (4)	3.0 (2)	2.9 (3)	3.3 (1)	3.3 (3)	3.0a
Google lens	3.1 (4)	2.7 (3)	3.1 (3)	2.7 (4)	2.8 (4)	3.1 (3)	3.1 (4)	2.9ab
PlantNet	2.7 (5)	2.7 (4)	2.4 (5)	2.2 (5)	2.4 (5)	2.5 (5)	2.4(7)	2.5bc
PlantSnap	2.1 (7)	2.3 (7)	2.0 (7)	2.2 (6)	2.0 (6)	2.1 (8)	2.7 (5)	2.1bcd
iPlant	2.3 (6)	2.6 (5)	2.2 (6)	1.7 (8)	2.0 (7)	2.1 (7)	2.4 (6)	2.1bcd
Candide	2.1 (8)	0.7(9)	1.1 (8)	1.8 (7)	1.6 (8)	2.3 (6)	0.8 (8)	1.6 cd
Bing	1.4 (9)	1.3 (8)	1.0 (9)	1.5 (9)	1.5 (9)	1.5 (9)	0.6 (9)	1.3d
Average	2.7	2.4	2.3	2.4	2.4	2.5	2.5	2.5

For any app it is useful to have an index of confidence in the identification. Only Flora Incognita, Plant.id and PlantNet return explicit confidence levels, though Seek has a particularly conservative identification strategy. Most of the other apps have only rudimentary delineation of confidence levels, sometimes only reporting when no clear identification has been achieved. In this study no account was taken of the confidence levels reported, but the confidence is likely to be a key criterion in more rigorous botanical applications.

### Camera

It should be noted that the precise scores obtained are very dependent on the type of sample and quality of the photograph used, which were purposefully very varied in this study. Most apps allow the use either of a live image taken with the smartphone camera or the uploading of a previously stored image. Where uploading is not explicitly incorporated it is still possible to store images for later display on a screen and reimaging (as done in all the present tests). When taking photos of a screen image on a smartphone, some apps (but especially Candide) introduced significant Moiré patterning (caused by the interaction between the pixel array of the screen and the imager: Oster and Nishijima 1963), this sometimes necessitated the capture of several images before a useable one could be obtained. This problem could be a function of the specific smartphone/screen combination used in this study and may have lowered the scores obtained (and reduced the consistency) for this app. Similar effects may apply to other apps in comparison with a direct use of the original image, but in all cases care was taken to only use images with minimal apparent Moiré patterning for the analyses, so I expect any such effect to have been small.

Another potentially valuable feature is the ability to use more than one image of a particular plant, for example of a leaf and of a flower. Plant.id, PlantNet and Flora Incognita all allow multiple images, indeed Flora Incognita prefers multiple images of specific views. Although this capacity was not tested in this study, with Flora Incognita an additional view of the same original image was sometimes required to facilitate identification and this was incorporated with a penalty **[see**  **Supporting Information—Table S1****]**.

### Internet requirement

All the image recognition apps discussed here, except Seek, appear to require internet access to make their identification, though it is always possible to store pictures for later identification. The ability to operate without internet connection is likely to be an important criterion in the choice of app. Seek just requires brief access in order to generate a list of species expected nearby (but note that if one is too rigorous in only allowing known species for the area, new species or arrivals would not be detectable).

### Who should be the users

An important question remains as to who the users of these automatic apps might be. For beginners and amateurs, such apps have great potential for providing shortcuts to the often technical or tedious keys in most botanical floras, at least suggesting the family or genus. A potential disadvantage, however, is that their ease of use may actually inhibit the development of botanical skills. With all apps at present, any suggested id always needs validation against conventional keys (whether on paper or a smartphone), or at least against a database of reliable images. More rigorous botanical studies such as those on biodiversity critically depend on accurate plant identification; automatic apps potentially have a role here but can only replace botanists if errors can be eliminated, or uncertain identifications flagged. In a simple test of the application of AI identifiers on a large data set, PlantNet has been used to validate (or otherwise) 31 973 ‘flower’ images retrieved from a social media platform (Flickr) in London (August *et al.* 2019). One could envisage use of such automated analysis in ecological surveys as long as adequately high confidence thresholds were adopted so that one could rely on the identifications. Unfortunately, because many plants can only be accurately identified using specific seasonal, cryptic or microscopic characters (frequently difficult even for trained botanists to identify; Bonnet *et al.* 2018), it is unlikely that this approach can by itself give full biodiversity information as required for a rigorous survey. Three of the image recognition apps have the capacity to obtain an expert identification as a supplement to any automatically derived identification. This is similar to those apps that rely on experts or the user community to provide accurate identifications (e.g. Flowerchecker (https://flowerchecker.com), iSpot (https://ispotnature.org) or SmartPlant (https://smartplantapp.com)).

### Other comments

Several of the apps allow users to confirm identification by feedback to the developers (see Table 1); this type of crowdsourcing information can lead to continuing improvement in the ability of the app to identify future samples (Nguyen *et al.* 2018). Nevertheless, I am concerned that without rigorous quality control of feedback such an approach can potentially build in error. Independent studies with PlantNet on the same images as used here (P. Bonnet, pers. comm.) provide an example of one app’s improvement over time from an identification percentage of c. 50 % for the programme’s version in December 2019 to 76 % for that in May 2018. The ability to tailor results tuned to the local flora is particularly useful for studies of native floras, as in ecological surveys, though only some of the apps target the relevant local flora. Of course, one use of these apps, especially ones aimed at gardeners (e.g. Candide), is to identify garden or alien plants which might not normally be recorded in the local flora. A potentially very useful development is that some of the apps can also be made available as Application Programming Interfaces (APIs) for incorporation into user software (see Table 1), such an approach will greatly widen the applicability of the technology.

It is worth noting that several of the apps evaluated here were disadvantaged by the methodology used, especially those such as Candide subject to Moiré patterning, and those designed to use several images (e.g. Flora Incognita, Plant.id and PlantNet), yet in spite of this they produced very creditable results across a wide range of images. In view of the very subjective choice of images used for testing it is not possible to state which app would be best in any particular situation, or how well the results will extrapolate to other regions, but the general rankings derived here are probably indicative.

## Conclusions

1. The potential for combining several images of different plant organs is a very powerful tool for enhancing identification performance (Nguyen *et al.* 2018; Rzanny *et al.* 2019). This useful tool is implemented in PlantNet, Plant.id, Flora Incognita, and though not tested in the present study, would be likely to improve accuracy further.
2. It is likely that any app giving a better than 50 % chance of the first suggestion being the correct genus or better (in this case Plant.id, Google Lens, Seek and Fora Incognita) would be a very valuable tool to botanists and ecologists in the field. A particular advantage of Seek is its ability to operate without internet connectivity.
3. These results confirm that automatic image recognition has now matured to a stage where it provides a powerful tool for field botanists in support of many botanical and ecological studies, with several viable apps readily available for free. Nevertheless, any suggested id still needs some independent confirmation, while for quantitative studies, further validation using experts or rigorous floras will still be required for some time, especially for rare or hard-to-distinguish species.
4. Notwithstanding the limitations for quantitative studies, any of the better-performing apps here should be of great value to beginners and amateurs in plant identification and may even stimulate interest in plants, plant identification and nature in general. There is, however, a concern that their ease of use may actually act as a disincentive to gaining further knowledge.
5. The performance of automatic plant identification apps is expected to improve rapidly, especially with the further incorporation of crowd-sourced data. Two of the tested apps are available as APIs for incorporation into other software; this will potentially open many new applications.

## Supporting Information

The following additional information is available in the online version of this article—

Table S1. This table summarizes the raw data for each replicate identification with each of the apps tested. The scoring systems used are detailed in Box 1 of the main paper. Note that suggested identifications are only shown until no further improvement in weighted score is made. Confidence levels are shown where available.

plaa052_suppl_Supplementary_Material

## Notes

**Form & Function. Chief Editor**: Kate McCulloh
