# Supplementary material for: What plant is that? Tests of automated image recognition apps for plant identification on plants from the British flora
Source: AoB Plants. 2020 Sep 23;12(6):plaa052. doi: 10.1093/aobpla/plaa052 (PMC7640754; doi:10.1093/aobpla/plaa052)
Supplement: plaa052_suppl_Supplementary_Material [file plaa052_suppl_supplementary_material.pdf]

Supplementary information - Table S1

N.B. 1. For Flora Incognita, sometimes it was necessary to show two views of the same photograph, before an identification could be achieved.
If so this was accepted as a first effort but with a penalty as listed under "Comments"
N.B. 2. Other comments where subjective adjustments are made to scores are also explained in "Comments"

Observation	Rep	Sample	App	Species	Woody/Herb/ Monocot	Part (F/Lf/Pl)	I. 1st effort	II. Weighted Score	Variability	Comment	1st	2nd	3rd	4th	#wrong
1	1	P.lutea	Plant.id	Psuedofumaria lutea	Herb	Plant	98	98	1	v.old name	Borkhausenia lutea(78%)				
2	2	P.lutea	Plant.id	Psuedofumaria lutea	Herb	Plant	98	98		v.old name	Borkhausenia lutea(95%)				
3	3	P.lutea	Plant.id	Psuedofumaria lutea	Herb	Plant	98	98		v.old name	Borkhausenia lutea(84%)				
4	4	P.lutea	Plant.id	Psuedofumaria lutea	Herb	Plant	98	98		v.old name	Borkhausenia lutea(93%)				
5	5	P.lutea	Plant.id	Psuedofumaria lutea	Herb	Plant	98	98		v.old name	Borkhausenia lutea(86%)				
6	1	D.purp.	Plant.id	Digitalis purpurea	Herb	Leaf	0	50	2		Borago officinalis(52%)	D.purpurea(27%)			1
7	2	D.purp.	Plant.id	Digitalis purpurea	Herb	Leaf	100	100			D.purpurea(58%)				
8	3	D.purp.	Plant.id	Digitalis purpurea	Herb	Leaf	100	100			D.purpurea(55%)				
9	4	D.purp.	Plant.id	Digitalis purpurea	Herb	Leaf	0	50			Borago officinalis(33%)	D.purpurea(32%)			1
10	5	D.purp.	Plant.id	Digitalis purpurea	Herb	Leaf	100	100			D.purpurea(28%)				
11	1	S.coron.	Plant.id	Silene coronaria	Herb	Leaf	100	100	2		Lychnis coronaria(56%)				
12	2	S.coron.	Plant.id	Silene coronaria	Herb	Leaf	100	100			Lychnis coronaria(44%)				
13	3	S.coron.	Plant.id	Silene coronaria	Herb	Leaf	100	100			Lychnis coronaria(43%)				
14	4	S.coron.	Plant.id	Silene coronaria	Herb	Leaf	100	100			Lychnis coronaria(30%)				
15	5	S.coron.	Plant.id	Silene coronaria	Herb	Leaf	10	33			Verbascum thapsus(21%)	Verbascum(20%)	L.coronaria(11%)		1
16	1	C.off.	Plant.id	Calendula officinalis	Herb	Plant	100	100	1		C.officinalis(47%)				
17	2	C.off.	Plant.id	Calendula officinalis	Herb	Plant	100	100			C.officinalis(72%)				
18	3	C.off.	Plant.id	Calendula officinalis	Herb	Plant	100	100			C.officinalis(50%)				
19	4	C.off.	Plant.id	Calendula officinalis	Herb	Plant	100	100			C.officinalis(68%)				
20	5	C.off.	Plant.id	Calendula officinalis	Herb	Plant	100	100			C.officinalis(35%)				
21	1	M.erecta	Plant.id	Moenchia erecta	Herb	Flower	0	50	2		Ornithogallum umbellatum(46%)	M.erecta(16%)			1
22	2	M.erecta	Plant.id	Moenchia erecta	Herb	Flower	0	50			Ornithogallum umbellatum(28%)	M.erecta(18%)			1
23	3	M.erecta	Plant.id	Moenchia erecta	Herb	Flower	0	13			Ornithogallum umbellatum(29%)	Thelymitra(8%)	Stellaria		1
24	4	M.erecta	Plant.id	Moenchia erecta	Herb	Flower	100	100			M.erecta(33%)				
25	5	M.erecta	Plant.id	Moenchia erecta	Herb	Flower	100	100			M.erecta(29%)				
26	1	S.verna	Plant.id	Sabulina verna	Herb	Plant	50	75	2.8	Minuartia=Sabulina	Sagina nodosa(28%)	M. verna(11%)			1
27	2	S.verna	Plant.id	Sabulina verna	Herb	Plant	80	90			Minuartia michauxii(12%)	M.verna(12%)			
28	3	S.verna	Plant.id	Sabulina verna	Herb	Plant	0	0			Saxifraga(17%)	Ornithogallum(9%)	Ornithogallum		1
29	4	S.verna	Plant.id	Sabulina verna	Herb	Plant	50	75			Sagina nodosa(11%)	M.verna(12%)			1
30	5	S.verna	Plant.id	Sabulina verna	Herb	Plant	50	80			M.laricifolia(9%)	Polytrichum juniperinum(7%)	Polytrichum commune		
31	1	E.marit.	Plant.id	Erodium maritimum	Herb	Leaf	0	0	4		Veronica persica(12%)	Sedum(11%)	Leptinella		1
32	2	E.marit.	Plant.id	Erodium maritimum	Herb	Leaf	0	0			Chrysanthemum(13%)	Aster(7%)	Leucanthemum		1
33	3	E.marit.	Plant.id	Erodium maritimum	Herb	Leaf	0	0			Ranunculus parviflorus(17%)	Leptinella(10%)	Veronica		1
34	4	E.marit.	Plant.id	Erodium maritimum	Herb	Leaf	0	0			Ranunculus parviflorus(27%)	Senecio(13)	Veronica		1
35	5	E.marit.	Plant.id	Erodium maritimum	Herb	Leaf	0	0			Senecio jacobaea(19%)	Cucurbita pepo(6%)	Ranunculus parviflorus		1
36	1	G.purp.	Plant.id	Geranium purpureum	Herb	Plant	80	90	1		G.carolinianum(36%)	G.purpureum(12%)			
37	2	G.purp.	Plant.id	Geranium purpureum	Herb	Plant	80	90			G.robertianum(65%)	G.purpureum(13%)			
38	3	G.purp.	Plant.id	Geranium purpureum	Herb	Plant	80	90			G.robertianum(75%)	G.purpureum(10%)			
39	4	G.purp.	Plant.id	Geranium purpureum	Herb	Plant	80	90			G.robertianum(83%)	G.purpureum(5%)			
40	5	G.purp.	Plant.id	Geranium purpureum	Herb	Plant	80	90			G.robertianum(68%)	G.purpureum(7%)			
41	1	A.diandra	Plant.id	Anisantha diandra	Monocot	Flower	90	93	2	B.sterilis v similar; Bromus and Anisantha accepted	B.sterilis(32%)	Bromus(12%)	B.diandrus		
42	2	A.diandra	Plant.id	Anisantha diandra	Monocot	Flower	10	10		B.sterilis v similar; Bromus and Anisantha accepted	Cyperus pohlii(39%)	Cyperus(9.4)	Phragmites		1
43	3	A.diandra	Plant.id	Anisantha diandra	Monocot	Flower	10	54		B.sterilis v similar; Bromus and Anisantha accepted	Cyperus pohlii(35%)	Bromus(9.3)	B.sterilis		1
44	4	A.diandra	Plant.id	Anisantha diandra	Monocot	Flower	90	93			B.sterilis(33%)	Bromus(14%)	B.diandrus		
45	5	A.diandra	Plant.id	Anisantha diandra	Monocot	Flower	90	95			B.sterilis(32%)	B.diandrus(13%)			
46	1	S.arv.-lf	Plant.id	Spergula arvensis	Herb	Leaf	100	100	1		S.arvensis(92%)				
47	2	S.arv.-lf	Plant.id	Spergula arvensis	Herb	Leaf	100	100			S.arvensis(66%)				
48	3	S.arv.-lf	Plant.id	Spergula arvensis	Herb	Leaf	100	100			S.arvensis(80%)				
49	4	S.arv.-lf	Plant.id	Spergula arvensis	Herb	Leaf	100	100			S.arvensis(92%)				
50	5	S.arv.-lf	Plant.id	Spergula arvensis	Herb	Leaf	100	100			S.arvensis(92%)	Pinus			
51	1	S.arv.-fl	Plant.id	Spergula arvensis	Herb	Flower	100	100	1		S.arvensis(92%)				
52	2	S.arv.-fl	Plant.id	Spergula arvensis	Herb	Flower	100	100			S.arvensis(81%)				
53	3	S.arv.-fl	Plant.id	Spergula arvensis	Herb	Flower	100	100			S.arvensis(76%)				
54	4	S.arv.-fl	Plant.id	Spergula arvensis	Herb	Flower	100	100			S.arvensis(92%)	S.media(3%)			
55	5	S.arv.-fl	Plant.id	Spergula arvensis	Herb	Flower	100	100			S.arvensis(93%)	S.media(3%)			
56	1	S.arv.-pl	Plant.id	Spergula arvensis	Herb	Plant	100	100	5		S.arvensis(45%)				
57	2	S.arv.-pl	Plant.id	Spergula arvensis	Herb	Plant	0	0			Erodium circuitarum(7%)	Claytonia(15%)	Hepatica		1
58	3	S.arv.-pl	Plant.id	Spergula arvensis	Herb	Plant	0	0			Ornithogallum umbellatum(45%)	Ornithogallum(13%)	Zephyranthes		1
59	4	S.arv.-pl	Plant.id	Spergula arvensis	Herb	Plant	0	0			Claytonia virginica(34%)	Hepatica nobilis(14%)	Cichorium		1
60	5	S.arv.-pl	Plant.id	Spergula arvensis	Herb	Plant	0	0			Hepatica nobilis(34%)	Dismophyta(&%)	Wahlenbergia		1
61	1	S.retic.	Plant.id	Salix reticulata	Woody	Leaf	100	100	1		S.reticulata(93%)				
62	2	S.retic.	Plant.id	Salix reticulata	Woody	Leaf	100	100			S.reticulata(86%)				
63	3	S.retic.	Plant.id	Salix reticulata	Woody	Leaf	100	100			S.reticulata(75%)				
64	4	S.retic.	Plant.id	Salix reticulata	Woody	Leaf	100	100			S.reticulata(78%)				
65	5	S.retic.	Plant.id	Salix reticulata	Woody	Leaf	100	100			S.reticulata(66%)				
66	1	A.sylv-fl	Plant.id	Angelica sylvestris	Herb	Flower	100	100	1		A.sylvestris(28%)				
67	2	A.sylv-fl	Plant.id	Angelica sylvestris	Herb	Flower	100	100			A.sylvestris(85%)				
68	3	A.sylv-fl	Plant.id	Angelica sylvestris	Herb	Flower	100	100			A.sylvestris(85%)				
69	4	A.sylv-fl	Plant.id	Angelica sylvestris	Herb	Flower	100	100			A.sylvestris(47%)				
70	5	A.sylv-fl	Plant.id	Angelica sylvestris	Herb	Flower	100	100			A.sylvestris(61%)				
71	1	A.sylv-lf	Plant.id	Angelica sylvestris	Herb	Leaf	100	100	2		A.sylvestris(47%)				
72	2	A.sylv-lf	Plant.id	Angelica sylvestris	Herb	Leaf	100	100			A.sylvestris(23%)				
73	3	A.sylv-lf	Plant.id	Angelica sylvestris	Herb	Leaf	100	100			A.sylvestris(48%)				
74	4	A.sylv-lf	Plant.id	Angelica sylvestris	Herb	Leaf	100	100			A.sylvestris(17%)				
75	5	A.sylv-lf	Plant.id	Angelica sylvestris	Herb	Leaf	0	13			Rosa pendulina(15%)	Sambucus nigra(9%)	Rosa(8%)	A.sylvestris(6%)	1
76	1	A.syl-pl	Plant.id	Angelica sylvestris	Herb	Plant	100	100	1		A.sylvestris(89%)				
77	2	A.syl-pl	Plant.id	Angelica sylvestris	Herb	Plant	100	100			A.sylvestris(64%)				
78	3	A.syl-pl	Plant.id	Angelica sylvestris	Herb	Plant	100	100			A.sylvestris(64%)				
79	4	A.syl-pl	Plant.id	Angelica sylvestris	Herb	Plant	100	100			A.sylvestris(73%)				
80	5	A.syl-pl	Plant.id	Angelica sylvestris	Herb	Plant	100	100			A.sylvestris(74%)				
81	1	A.sylv-fr	Plant.id	Angelica sylvestris	Herb	Fruit	0	0	3.2		Allium porrum(15%)	A.sylvestris(12%)%)			1
82	2	A.sylv-fr	Plant.id	Angelica sylvestris	Herb	Fruit	0	0			Hydrangea(10%)	Allium porrum(9%)	Hydrangea		1
83	3	A.sylv-fr	Plant.id	Angelica sylvestris	Herb	Fruit	0	0			Porrum sphaerocephalon(8%)	Trifolium(7%)	Hydrangea		1
84	4	A.sylv-fr	Plant.id	Angelica sylvestris	Herb	Fruit	0	0			Chrysanthemum(15%)	Dahli(a14%)	Musa		1
85	5	A.sylv-fr	Plant.id	Angelica sylvestris	Herb	Fruit	0	0			Allium porrum(34%)	Porrum sphaerocephalon(1 0%)			1
86	1	A.caucal.-fl	Plant.id	Anthriscus caucalis	Herb	Flower	100	100	1		A.caucalis(93%)				
87	2	A.caucal.-fl	Plant.id	Anthriscus caucalis	Herb	Flower	100	100			A.caucalis(48%)				
88	3	A.caucal.-fl	Plant.id	Anthriscus caucalis	Herb	Flower	100	100			A.caucalis(41%)				
89	4	A.caucal.-fl	Plant.id	Anthriscus caucalis	Herb	Flower	100	100			A.caucalis(53%)				
90	5	A.caucal.-fl	Plant.id	Anthriscus caucalis	Herb	Flower	100	100			A.caucalis(35%)				
91	1	A.caucal.-pl	Plant.id	Anthriscus caucalis	Herb	Plant	100	100	3.2		A.caucalis(95%)				

92	2	A.caucal.-pl	Plant.id	Anthriscus caucalis	Herb	Plant	50	78			Scandix pecten-veneris(20%)	A.sylvestris(19%)	A.caucalis		1
93	3	A.caucal.-pl	Plant.id	Anthriscus caucalis	Herb	Plant	80	92		A.sylvestris=90	A.sylvestris(41%)	Scandix(19%)	A.cerefolium	A.caucalis	
94	4	A.caucal.-pl	Plant.id	Anthriscus caucalis	Herb	Plant	0	23			Cardamine(15%)	Cardamine(10%)	A.sylvestris(8%)		1
95	5	A.caucal.-pl	Plant.id	Anthriscus caucalis	Herb	Plant	50	60			Scandix pecten-veneris(34%)	Achillea millefolium(16%)	A.sylvestris(12%)		1
96	1	A.caucal-lf	Plant.id	Anthriscus caucalis	Herb	Leaf	0	25	2		Tanacetum vulgare(24%)	Daucus(13%)	Conium(10%)		1
97	2	A.caucal-lf	Plant.id	Anthriscus caucalis	Herb	Leaf	-5	-5			Davallia(17%)	Thuja(13%)	Asplenium		1
98	3	A.caucal-lf	Plant.id	Anthriscus caucalis	Herb	Leaf	0	0			Tanacetum vulgare(24%)	Davallia(21%)	Phacelia		1
99	4	A.caucal-lf	Plant.id	Anthriscus caucalis	Herb	Leaf	0	42			Tanacetum vulgare(18%)	Conium maculatum(16%)	A.sylvestris		1
100	5	A.caucal-lf	Plant.id	Anthriscus caucalis	Herb	Leaf	0	33			Tanacetum vulgare(30%)	Conium maculatum(5%)	Senecio	A.sylvestris	1
101	1	H.elod-Fl	Plant.id	Hypericum elodes	Herb	Flower	0	0	5		Vebascum thapsus22%)	Verbascum(9%0	V. pulverentum		1
102	2	H.elod-Fl	Plant.id	Hypericum elodes	Herb	Flower	0	0			Oxalis pes-caprea(22%)	Primular vulgaris(17%)	Caltha		1
103	3	H.elod-Fl	Plant.id	Hypericum elodes	Herb	Flower	-5	-5			Phalaenopsis(17)	Caltha(15%)	Oxalis(5)		1
104	4	H.elod-Fl	Plant.id	Hypericum elodes	Herb	Flower	0	0			Oxalis corniculata(8%)	Digitalis(7%)	Pistia		1
105	5	H.elod-Fl	Plant.id	Hypericum elodes	Herb	Flower	100	100			H.elodes(26.3)				
106	1	H.elod-lf	Plant.id	Hypericum elodes	Herb	Leaf	0	2	2	Similar to Stachys byzantina/Verbascum	Ocimum basilicum(15%)	Origanum(6%)	Mentha suaveolens	Stachys byzantina	1
107	2	H.elod-lf	Plant.id	Hypericum elodes	Herb	Leaf	10	10		Similar to Stachys byzantina/Verbascum	Stachys byzantina(58%)	Verbascum thapsus(10%)	Verbascum		1
108	3	H.elod-lf	Plant.id	Hypericum elodes	Herb	Leaf	10	10		Similar to Stachys byzantina/Verbascum	Stachys byzantina(58%)	Verbascum thapsus(6%)	Mentha		1
109	4	H.elod-lf	Plant.id	Hypericum elodes	Herb	Leaf	10	10			Stachys byzantina(38%)	Verbascum thapsus(26%)			1
110	5	H.elod-lf	Plant.id	Hypericum elodes	Herb	Leaf	10	10			Stachys byzantina(42%)	Tradescantia(15%)			1
111	1	H.elod-pl	Plant.id	Hypericum elodes	Herb	Plant	100	100	2		H. elodes (94%)				
112	2	H.elod-pl	Plant.id	Hypericum elodes	Herb	Plant	0	0			Lysimachia nummularia(57%)	Oreganum(7%)	Euphorbia amygdaloides		1
113	3	H.elod-pl	Plant.id	Hypericum elodes	Herb	Plant	0	50			Lysimachia nummularia(23%)	H.elodes(16)			1
114	4	H.elod-pl	Plant.id	Hypericum elodes	Herb	Plant	100	100			H.elodes(83%)				
115	5	H.elod-pl	Plant.id	Hypericum elodes	Herb	Plant	100	100			H.elodes(85%)				
116	1	C.remot-fl1	Plant.id	Carex remota	Monocot	Flower	80	85	1		C.pallescens(11%)	Anthoxanthum(7%)	C.remota		
117	2	C.remot-fl1	Plant.id	Carex remota	Monocot	Flower	80	90			C.pallescens(15%)	C.remota(7%)			
118	3	C.remot-fl1	Plant.id	Carex remota	Monocot	Flower	100	100			C.remota(45%)				
119	4	C.remot-fl1	Plant.id	Carex remota	Monocot	Flower	100	100			C.remota(20%)				
120	5	C.remot-fl1	Plant.id	Carex remota	Monocot	Flower	80	90		v. similar sp	C.canescens(24%)				
121	1	C.remot-pl	Plant.id	Carex remota	Monocot	Plant	10	25	4		Juncus effusus(18%)	Schoenus nigricans(9%)	Apodasmia		1
122	2	C.remot-pl	Plant.id	Carex remota	Monocot	Plant	-5	5			Equisetum hyemale(23%)	J.effusus(11%)	Apodasmia		1
123	3	C.remot-pl	Plant.id	Carex remota	Monocot	Plant	10	10		Apodasmia similar	Apodasmia(17%)	Ammophila(11%)	J.effusus		1
124	4	C.remot-pl	Plant.id	Carex remota	Monocot	Plant	10	10			Juncus effusus(25%)	Equisetum hyemale(15%)	Juncus	Schoenus	1
125	5	C.remot-pl	Plant.id	Carex remota	Monocot	Plant	10	10		Apodasmia similar	Apodasmia(18%)	Equisetum(18%)	Juncus		1
126	1	C.remot-fl2	Plant.id	Carex remota	Monocot	Flower	-5	43	2		Heliconia (15%)	C. pallescens(8%)	C.hirta		1
127	2	C.remot-fl2	Plant.id	Carex remota	Monocot	Flower	-5	43			Heliconia (9%)	C.hirtifolia((5%)	C.blanda		1
128	3	C.remot-fl2	Plant.id	Carex remota	Monocot	Flower	-5	43			Heliconia (16%)	C.pallescens(8%)	C.hirta		1
129	4	C.remot-fl2	Plant.id	Carex remota	Monocot	Flower	80	90		v.similar sp	C.canescens(9%)				
130	5	C.remot-fl2	Plant.id	Carex remota	Monocot	Flower	80	80			C.hirta(10%)				
131	1	Q.rob-lf	Plant.id	Quercus robur	Woody	Leaf	100	100	1		Q.robur(33%)				
132	2	Q.rob-lf	Plant.id	Quercus robur	Woody	Leaf	100	100			Q.robur(27%)				
133	3	Q.rob-lf	Plant.id	Quercus robur	Woody	Leaf	100	100			Q.robur(31%)				
134	4	Q.rob-lf	Plant.id	Quercus robur	Woody	Leaf	100	100			Q.robur(68%)				
135	5	Q.rob-lf	Plant.id	Quercus robur	Woody	Leaf	100	100			Q.robur(71%)				
136	1	Q.rob-fr	Plant.id	Quercus robur	Woody	Fruit	100	100	1		Q.robur(22%)				
137	2	Q.rob-fr	Plant.id	Quercus robur	Woody	Fruit	100	100			Q.robur(28%)				
138	3	Q.rob-fr	Plant.id	Quercus robur	Woody	Fruit	100	100			Q.robur(34%)				
139	4	Q.rob-fr	Plant.id	Quercus robur	Woody	Fruit	100	100			Q.robur(27%)				
140	5	Q.rob-fr	Plant.id	Quercus robur	Woody	Fruit	100	100			Q.robur(28%)				
141	1	Q.rob-pl	Plant.id	Quercus robur	Woody	Plant	0	0	3		Portulacaria afra(32%)	Acer campestre(14%)	Ilex aquifolium		1
142	2	Q.rob-pl	Plant.id	Quercus robur	Woody	Plant	80	80			Q.ilex(14%)	Q.coccifera(7%)	Platanus		
143	3	Q.rob-pl	Plant.id	Quercus robur	Woody	Plant	80	80			Q.ilex(7%)	Platanus(7%)			
144	4	Q.rob-pl	Plant.id	Quercus robur	Woody	Plant	10	55			Platanus orientalis(14%)	Q.robur(12%)			1
145	5	Q.rob-pl	Plant.id	Quercus robur	Woody	Plant	80	80			Q.ilex(15%)	Q.Coccifera(7%)			
146	1	E.nigr-fl	Plant.id	Empetrum nigrum	Woody	Flower	100	100	1		E.nigrum(14%)				
147	2	E.nigr-fl	Plant.id	Empetrum nigrum	Woody	Flower	100	100			E.nigrum(67%)				
148	3	E.nigr-fl	Plant.id	Empetrum nigrum	Woody	Flower	100	100			E.nigrum(61%)				
149	4	E.nigr-fl	Plant.id	Empetrum nigrum	Woody	Flower	100	100			E.nigrum(70%)				
150	5	E.nigr-fl	Plant.id	Empetrum nigrum	Woody	Flower	100	100			E.nigrum(60%)				
151	1	E.nigr-fr	Plant.id	Empetrum nigrum	Woody	Fruit	100	100	1		E.nigrum(66%)				
152	2	E.nigr-fr	Plant.id	Empetrum nigrum	Woody	Fruit	100	100			E.nigrum(71%)				
153	3	E.nigr-fr	Plant.id	Empetrum nigrum	Woody	Fruit	100	100			E.nigrum(69%)				
154	4	E.nigr-fr	Plant.id	Empetrum nigrum	Woody	Fruit	100	100			E.nigrum(65%)				
155	5	E.nigr-fr	Plant.id	Empetrum nigrum	Woody	Fruit	100	100			E.nigrum(69%)				
156	1	A.pseudo	Plant.id	Acer pseudoplatanus	Woody	Leaf	80	90	1.2		Acer opalus(34%)	A.pseudoplatanus(27%)			
157	2	A.pseudo	Plant.id	Acer pseudoplatanus	Woody	Leaf	80	90			Acer opalus(35%)	A.pseudoplatanus(31%)			
158	3	A.pseudo	Plant.id	Acer pseudoplatanus	Woody	Leaf	100	100			A.pseudoplatanus(29%)	Acer opalus(17%)			
159	4	A.pseudo	Plant.id	Acer pseudoplatanus	Woody	Leaf	100	100			A.pseudoplatanus(23%)				
160	5	A.pseudo	Plant.id	Acer pseudoplatanus	Woody	Leaf	100	100			A.pseudoplatanus(20%)				
161	1	C.pauci	Plant.id	Carex pauciflora	Monocot	Flower	10	65	1.2		Dulichium arundinaceum(24%)	C.intumescens(12%)	C.radiata		1
162	2	C.pauci	Plant.id	Carex pauciflora	Monocot	Flower	100	100			C.pauciflora(60%)				
163	3	C.pauci	Plant.id	Carex pauciflora	Monocot	Flower	100	100			C.pauciflora(73%)				
164	4	C.pauci	Plant.id	Carex pauciflora	Monocot	Flower	100	100			C.pauciflora(49%)				
165	5	C.pauci	Plant.id	Carex pauciflora	Monocot	Flower	100	100			C.pauciflora(72%)				
166	1	C.Fuscus	Plant.id	Cyperus fuscus	Monocot	Plant	100	100	1		C.fuscus(50%)				
167	2	C.Fuscus	Plant.id	Cyperus fuscus	Monocot	Plant	100	100			C.fuscus(75%)				
168	3	C.Fuscus	Plant.id	Cyperus fuscus	Monocot	Plant	100	100			C.fuscus(63%)				
169	4	C.Fuscus	Plant.id	Cyperus fuscus	Monocot	Plant	100	100			C.fuscus(50%)				
170	5	C.Fuscus	Plant.id	Cyperus fuscus	Monocot	Plant	100	100			C.fuscus(55%)				
171	1	T.marit	Plant.id	Triglochin maritima	Monocot	Plant	100	100	1		T.maritima(41%)				
172	2	T.marit	Plant.id	Triglochin maritima	Monocot	Plant	100	100			T.maritima(38%)				
173	3	T.marit	Plant.id	Triglochin maritima	Monocot	Plant	100	100			T.maritima(60%)				
174	4	T.marit	Plant.id	Triglochin maritima	Monocot	Plant	100	100			T.maritima(38%)				
175	5	T.marit	Plant.id	Triglochin maritima	Monocot	Plant	100	100			T.maritima(36%)				
176	1	Delairia	Plant.id	Delairea odorata	Herb	Plant	100	100	1		D.odorata(83%)				
177	2	Delairia	Plant.id	Delairea odorata	Herb	Plant	100	100			D.odorata(91%)				
178	3	Delairia	Plant.id	Delairea odorata	Herb	Plant	100	100			D.odorata(61%)				
179	4	Delairia	Plant.id	Delairea odorata	Herb	Plant	100	100			D.odorata(84%)				
180	5	Delairia	Plant.id	Delairea odorata	Herb	Plant	100	100			D.odorata(58%)				
181	1	E.bonar	Plant.id	Erigeron bonariensis	Herb	Flower	100	100	1	Erigeron=Conyza	Conyza bonariensis(51%)				
182	2	E.bonar	Plant.id	Erigeron bonariensis	Herb	Flower	100	100			Conyza bonariensis(49%)				
183	3	E.bonar	Plant.id	Erigeron bonariensis	Herb	Flower	100	100			Conyza bonariensis(61%)				
184	4	E.bonar	Plant.id	Erigeron bonariensis	Herb	Flower	100	100			Conyza bonariensis(61%)				
185	5	E.bonar	Plant.id	Erigeron bonariensis	Herb	Flower	100	100			Conyza bonariensis(55%)				
186	1	M.mosch	Plant.id	Malva moschata	Herb	Flower	100	100	1		M.moschata(92%)				
187	2	M.mosch	Plant.id	Malva moschata	Herb	Flower	100	100			M.moschata(88%)				
188	3	M.mosch	Plant.id	Malva moschata	Herb	Flower	100	100			M.moschata(91%)				
189	4	M.mosch	Plant.id	Malva moschata	Herb	Flower	100	100			M.moschata(91%)				
190	5	M.mosch	Plant.id	Malva moschata	Herb	Flower	100	100			M.moschata(92%)				
191	1	P.lutea	Google Lens	Psuedofumaria lutea	Herb	Plant	100	100	1.2		P.lutea				
192	2	P.lutea	Google Lens	Psuedofumaria lutea	Herb	Plant	80	90			Corydalis flavula	P.lutea			
193	3	P.lutea	Google Lens	Psuedofumaria lutea	Herb	Plant	100	100			P.lutea				
194	4	P.lutea	Google Lens	Psuedofumaria lutea	Herb	Plant	100	100			P.lutea				

195	5	P.lutea	Google Lens	Psuedofumaria lutea	Herb	Plant	100	100			P.lutea			
196	1	D.purp.	Google Lens	Digitalis purpurea	Herb	Leaf	100	100	3		Lady's glove			
197	2	D.purp.	Google Lens	Digitalis purpurea	Herb	Leaf	-5	-5			Cactus	Borage	Irrelevant	1
198	3	D.purp.	Google Lens	Digitalis purpurea	Herb	Leaf	-5	-5			Cactus	Borage	Labiate	1
199	4	D.purp.	Google Lens	Digitalis purpurea	Herb	Leaf	100	100			Lady's glove			
200	5	D.purp.	Google Lens	Digitalis purpurea	Herb	Leaf	0	50			Borage	Lady's glove		1
201	1	S.coron.	Google Lens	Silene coronaria	Herb	Leaf	10	33	1.2	Stachys/Verbascum both10	Gt mullein	Lamb's ear	Rose campion	1
202	2	S.coron.	Google Lens	Silene coronaria	Herb	Leaf	10	55		Stachys/Verbascum both10	Gt mullein	Rose campion		1
203	3	S.coron.	Google Lens	Silene coronaria	Herb	Leaf	10	55		Stachys/Verbascum both10	Mullein	Rose campion		1
204	4	S.coron.	Google Lens	Silene coronaria	Herb	Leaf	10	55			Mullein	Rose campion		1
205	5	S.coron.	Google Lens	Silene coronaria	Herb	Leaf	10	33			Gt mullein	Lamb's ear	Rose campion	1
206	1	C.off.	Google Lens	Calendula officinalis	Herb	Plant	100	100	1		Pot marigold			
207	2	C.off.	Google Lens	Calendula officinalis	Herb	Plant	100	100			Pot marigold			
208	3	C.off.	Google Lens	Calendula officinalis	Herb	Plant	100	100			Pot marigold			
209	4	C.off.	Google Lens	Calendula officinalis	Herb	Plant	100	100			Pot marigold			
210	5	C.off.	Google Lens	Calendula officinalis	Herb	Plant	100	100			Pot marigold			
211	1	M.erecta	Google Lens	Moenchia erecta	Herb	Flower	50	50	2.2		Minuartia	Arenaria serpylifolia	A. ciliata	
212	2	M.erecta	Google Lens	Moenchia erecta	Herb	Flower	50	50			Arenaria ciliata	Cerastium fontanum	Minuartia	1
213	3	M.erecta	Google Lens	Moenchia erecta	Herb	Flower	50	50			Minuartia	Arenaria serpylifolia	Cerastium	1
214	4	M.erecta	Google Lens	Moenchia erecta	Herb	Flower	5	5		Slight similarity	Ornithogallum	Amana	Lomatogonium	1
215	5	M.erecta	Google Lens	Moenchia erecta	Herb	Flower	50	50			Minuartia	Arenaria	Cerastium	1
216	1	S.verna	Google Lens	Sabulina verna	Herb	Plant	50	50	1		Minuartia	Arenaria		
217	2	S.verna	Google Lens	Sabulina verna	Herb	Plant	50	50			Minuartia	Arenaria		
218	3	S.verna	Google Lens	Sabulina verna	Herb	Plant	50	50			Minuartia	Arenaria		
219	4	S.verna	Google Lens	Sabulina verna	Herb	Plant	50	50			Minuartia			
220	5	S.verna	Google Lens	Sabulina verna	Herb	Plant	50	50			Minuartia			
221	1	E.marit.	Google Lens	Erodium maritimum	Herb	Leaf	50	50	3		Chrysosplenium	Moschatel		1
222	2	E.marit.	Google Lens	Erodium maritimum	Herb	Leaf	0	25			Liverwort	Marchantia	Erodium	1
223	3	E.marit.	Google Lens	Erodium maritimum	Herb	Leaf	0	0			Liverwort	Leptinella	Moschatel	1
224	4	E.marit.	Google Lens	Erodium maritimum	Herb	Leaf	0	0			Saxifraga(17%)	Liverwort		1
225	5	E.marit.	Google Lens	Erodium maritimum	Herb	Leaf	0	0			Liverwort	Mitella		1
226	1	G.purp.	Google Lens	Geranium purpureum	Herb	Plant	100	100	2.2		Little robin			
227	2	G.purp.	Google Lens	Geranium purpureum	Herb	Plant	0	25			Lythrum hyssopifolia	Saxifraga	G.purpureum	1
228	3	G.purp.	Google Lens	Geranium purpureum	Herb	Plant	100	100			G.purpureum			
229	4	G.purp.	Google Lens	Geranium purpureum	Herb	Plant	100	100			G.purpureum			
230	5	G.purp.	Google Lens	Geranium purpureum	Herb	Plant	0	0			Lythrum hyssopifolia	Frankenia		1
231	1	A.diandra	Google Lens	Anisantha diandra	Monocot	Flower	90	90	1.2	B.inermis v close	B.inermis	Bromus		
232	2	A.diandra	Google Lens	Anisantha diandra	Monocot	Flower	90	90		B.inermis v close	B.inermis	B.arvensis		
233	3	A.diandra	Google Lens	Anisantha diandra	Monocot	Flower	90	90		B.inermis v close	B.inermis	B.sterilis		
234	4	A.diandra	Google Lens	Anisantha diandra	Monocot	Flower	90	90		B.inermis v close	B.madritensis	B.inermis	B.sterilis	
235	5	A.diandra	Google Lens	Anisantha diandra	Monocot	Flower	90	90		B.inermis v close	B.inermis	B.madritensis		
236	1	S.arv.-lf	Google Lens	Spergula arvensis	Herb	Leaf	100	100	1		S.arvensis			
237	2	S.arv.-lf	Google Lens	Spergula arvensis	Herb	Leaf	100	100			S.arvensis			
238	3	S.arv.-lf	Google Lens	Spergula arvensis	Herb	Leaf	100	100			S.arvensis			
239	4	S.arv.-lf	Google Lens	Spergula arvensis	Herb	Leaf	100	100			S.arvensis			
240	5	S.arv.-lf	Google Lens	Spergula arvensis	Herb	Leaf	100	100			S.arvensis			
241	1	S.arv.-fl	Google Lens	Spergula arvensis	Herb	Flower	100	100	1		S.arvensis			
242	2	S.arv.-fl	Google Lens	Spergula arvensis	Herb	Flower	100	100			S.arvensis			
243	3	S.arv.-fl	Google Lens	Spergula arvensis	Herb	Flower	100	100			S.arvensis			
244	4	S.arv.-fl	Google Lens	Spergula arvensis	Herb	Flower	100	100			S.arvensis			
245	5	S.arv.-fl	Google Lens	Spergula arvensis	Herb	Flower	100	100			S.arvensis			
246	1	S.arv.-pl	Google Lens	Spergula arvensis	Herb	Plant	0	25	2.2		Oldenlandia	Arenaria ciliata	Heydotis	1
247	2	S.arv.-pl	Google Lens	Spergula arvensis	Herb	Plant	50	50			Arenaria ciliata	Oldenlandia	Heydotis	1
248	3	S.arv.-pl	Google Lens	Spergula arvensis	Herb	Plant	50	75			Arenaria ciliata	S.arvensis		1
249	4	S.arv.-pl	Google Lens	Spergula arvensis	Herb	Plant	100	100			S.arvensis			
250	5	S.arv.-pl	Google Lens	Spergula arvensis	Herb	Plant	50	75			Arenaria ciliata	S.arvensis		1
251	1	S.retic.	Google Lens	Salix reticulata	Woody	Leaf	0	45	2		Gaultheria	Salix herbacea		1
252	2	S.retic.	Google Lens	Salix reticulata	Woody	Leaf	90	90			S.herbacea	S.arctica		
253	3	S.retic.	Google Lens	Salix reticulata	Woody	Leaf	90	90			S.herbacea	Arctuous alpina		
254	4	S.retic.	Google Lens	Salix reticulata	Woody	Leaf	90	90			S.herbacea	S.arctica		
255	5	S.retic.	Google Lens	Salix reticulata	Woody	Leaf	90	90			S.herbacea	S.arctica		
256	1	A.sylv-fl	Google Lens	Angelica sylvestris	Herb	Flower	100	100	1.2		Wild angelica			
257	2	A.sylv-fl	Google Lens	Angelica sylvestris	Herb	Flower	100	100			Wild angelica			
258	3	A.sylv-fl	Google Lens	Angelica sylvestris	Herb	Flower	100	100			Wild angelica			
259	4	A.sylv-fl	Google Lens	Angelica sylvestris	Herb	Flower	50	65			Water hemlock	Angelica arguta		1
260	5	A.sylv-fl	Google Lens	Angelica sylvestris	Herb	Flower	100	100			Wild angelica			
261	1	A.sylv-lf	Google Lens	Angelica sylvestris	Herb	Leaf	80	90	1		Angelica genuflexa	Angelica		
262	2	A.sylv-lf	Google Lens	Angelica sylvestris	Herb	Leaf	100	100			A.sylvestris			
263	3	A.sylv-lf	Google Lens	Angelica sylvestris	Herb	Leaf	100	100			A.sylvestris			
264	4	A.sylv-lf	Google Lens	Angelica sylvestris	Herb	Leaf	100	100			A.sylvestris			
265	5	A.sylv-lf	Google Lens	Angelica sylvestris	Herb	Leaf	90	95			Angelica	A.sylvestris		
266	1	A.syl-pl	Google Lens	Angelica sylvestris	Herb	Plant	100	100	1		Wild angelica			
267	2	A.syl-pl	Google Lens	Angelica sylvestris	Herb	Plant	100	100			Wild angelica			
268	3	A.syl-pl	Google Lens	Angelica sylvestris	Herb	Plant	100	100			Wild angelica			
269	4	A.syl-pl	Google Lens	Angelica sylvestris	Herb	Plant	100	100			Wild angelica			
270	5	A.syl-pl	Google Lens	Angelica sylvestris	Herb	Plant	100	100			Wild angelica			
271	1	A.sylv-fr	Google Lens	Angelica sylvestris	Herb	Fruit	0	45	3		Laserpitium	Angelica arguta		1
272	2	A.sylv-fr	Google Lens	Angelica sylvestris	Herb	Fruit	0	50			Laserpitium	A.sylvestris		1
273	3	A.sylv-fr	Google Lens	Angelica sylvestris	Herb	Fruit	-5	-5			"jade"	"seeds"	Irrelevant	1
274	4	A.sylv-fr	Google Lens	Angelica sylvestris	Herb	Fruit	100	100			Wild angelica			
275	5	A.sylv-fr	Google Lens	Angelica sylvestris	Herb	Fruit	100	100			Wild angelica			
276	1	A.caucal.-fl	Google Lens	Anthriscus caucalis	Herb	Flower	0	0	3.2		Cryptantha	Turgenia		1
277	2	A.caucal.-fl	Google Lens	Anthriscus caucalis	Herb	Flower	70	70			Torilis			
278	3	A.caucal.-fl	Google Lens	Anthriscus caucalis	Herb	Flower	0	25			Cornsalad	Mountain sweet cicely	Bifora	1
279	4	A.caucal.-fl	Google Lens	Anthriscus caucalis	Herb	Flower	70	70			Caucalis platycarpos			
280	5	A.caucal.-fl	Google Lens	Anthriscus caucalis	Herb	Flower	70	70		Torilis & Caucalis similar genera	Caucalis platycarpos	Torilis		
281	1	A.caucal.-pl	Google Lens	Anthriscus caucalis	Herb	Plant	0	25	3		Hoary alyssum	Scandix		1
282	2	A.caucal.-pl	Google Lens	Anthriscus caucalis	Herb	Plant	70	70			Caucalis platycarpos	Cow parsley		
283	3	A.caucal.-pl	Google Lens	Anthriscus caucalis	Herb	Plant	70	70			Caucalis platycarpos	Torilis nodosa		
284	4	A.caucal.-pl	Google Lens	Anthriscus caucalis	Herb	Plant	70	70			Caucalis platycarpos	Beteroa		
285	5	A.caucal.-pl	Google Lens	Anthriscus caucalis	Herb	Plant	-5	-5			"rockfoils"	"Herbaceous plant"		1
286	1	A.caucal-lf	Google Lens	Anthriscus caucalis	Herb	Leaf	50	60	1.6		Wild carrot	Chaerophyllum temulum		1
287	2	A.caucal-lf	Google Lens	Anthriscus caucalis	Herb	Leaf	70	75			Torilis nodosa	Daucus carota	Caucalis	
288	3	A.caucal-lf	Google Lens	Anthriscus caucalis	Herb	Leaf	70	70			Chaerophyllum bulbosus	Torilis		1
289	4	A.caucal-lf	Google Lens	Anthriscus caucalis	Herb	Leaf	50	60			Hogweed	Hemlock		1
290	5	A.caucal-lf	Google Lens	Anthriscus caucalis	Herb	Leaf	50	60			Wild carrot	Torilis nodosa		1
291	1	H.elod-Fl	Google Lens	Hypericum elodes	Herb	Flower	-5	-5	4		Tripodion	Irrelevant		1
292	2	H.elod-Fl	Google Lens	Hypericum elodes	Herb	Flower	-5	-5			"waxes"	Waltheria	Malvella	1
293	3	H.elod-Fl	Google Lens	Hypericum elodes	Herb	Flower	-5	-5			Flaxes	Gratiola	Irrelevant	1
294	4	H.elod-Fl	Google Lens	Hypericum elodes	Herb	Flower	-5	-5			Nonea	Tripodion		1
295	5	H.elod-Fl	Google Lens	Hypericum elodes	Herb	Flower	-5	-5			Flaxes	"Herbaceous plant"		1
296	1	H.elod-lf	Google Lens	Hypericum elodes	Herb	Leaf	-5	-5	4		Sideritis	Irrelevant		1
297	2	H.elod-lf	Google Lens	Hypericum elodes	Herb	Leaf	-5	-5			Tansey	Tradescantia	Sideritis	1
298	3	H.elod-lf	Google Lens	Hypericum elodes	Herb	Leaf	5	5			Torilis	Tradescantia	Lamb's ear	1
299	4	H.elod-lf	Google Lens	Hypericum elodes	Herb	Leaf	-5	-5			Torilis	Tradescantia	Sideritis	1
300	5	H.elod-lf	Google Lens	Hypericum elodes	Herb	Leaf	10	10			Lamb's ear	Gt mullein		
301	1	H.elod-pl	Google Lens	Hypericum elodes	Herb	Plant	-5	-5	4.2		Musk mallow	Irrelevant		1
302	2	H.elod-pl	Google Lens	Hypericum elodes	Herb	Plant	50	50			Hypericum anagalloides	Abeloschus	Echballium	
303	3	H.elod-pl	Google Lens	Hypericum elodes	Herb	Plant	0	0			Oxalis pes-caprea(22%)	Bacopa		1
304	4	H.elod-pl	Google Lens	Hypericum elodes	Herb	Plant	25	25			Petrocosmia	H.anagalloides	Lysimachia	1
305	5	H.elod-pl	Google Lens	Hypericum elodes	Herb	Plant	0	0			Creeping woodsorrel	Irrelevant		1
306	1	C.remot-fl1	Google Lens	Carex remota	Monocot	Flower	90	90	2	v. similar sp	C.canescens	C.brunnescens		
307	2	C.remot-fl1	Google Lens	Carex remota	Monocot	Flower	90	90		v. similar sp	C.canescens	C.scoparia		

308	3	C.remot-fl1	Google Lens	Carex remota	Monocot	Flower	90	90		v. similar sp	C.canescens	C.leorina			
309	4	C.remot-fl1	Google Lens	Carex remota	Monocot	Flower	0	45			Musk mallow	C.canescens			1
310	5	C.remot-fl1	Google Lens	Carex remota	Monocot	Flower		90			C.canescens	C.echinata			
311	1	C.remot-pl	Google Lens	Carex remota	Monocot	Plant	80	85	1.2		Elongated sedge	C.canescens			
312	2	C.remot-pl	Google Lens	Carex remota	Monocot	Plant	80	80			C.pelita	C.lasiocarpa			
313	3	C.remot-pl	Google Lens	Carex remota	Monocot	Plant	80	80			C.hirta	C.pelita			
314	4	C.remot-pl	Google Lens	Carex remota	Monocot	Plant	80	85			C.hirta	C.canescens			
315	5	C.remot-pl	Google Lens	Carex remota	Monocot	Plant	80	80			C.pelita	C.hirta			
316	1	C.remot-fl2	Google Lens	Carex remota	Monocot	Flower	10	10	4		Puccinellia	Nardus			1
317	2	C.remot-fl2	Google Lens	Carex remota	Monocot	Flower	10	10			Puccinellia	Spreading rush			1
318	3	C.remot-fl2	Google Lens	Carex remota	Monocot	Flower	-5	3			Bamboo	Sweet grass	Poa		1
319	4	C.remot-fl2	Google Lens	Carex remota	Monocot	Flower	10	10			Festuca	Coryneophorus			1
320	5	C.remot-fl2	Google Lens	Carex remota	Monocot	Flower	10	10			Bluestem	Heteropogon			1
321	1	Q.rob-lf	Google Lens	Quercus robur	Woody	Leaf	80	80	1.2		Quercus				
322	2	Q.rob-lf	Google Lens	Quercus robur	Woody	Leaf	100	100			Q.robur				
323	3	Q.rob-lf	Google Lens	Quercus robur	Woody	Leaf	100	100			Q.robur				
324	4	Q.rob-lf	Google Lens	Quercus robur	Woody	Leaf	100	100			Q.robur				
325	5	Q.rob-lf	Google Lens	Quercus robur	Woody	Leaf	100	100			Q.robur				
326	1	Q.rob-fr	Google Lens	Quercus robur	Woody	Fruit	100	100	1		Q.robur				
327	2	Q.rob-fr	Google Lens	Quercus robur	Woody	Fruit	100	100			Q.robur				
328	3	Q.rob-fr	Google Lens	Quercus robur	Woody	Fruit	100	100			Q.robur				
329	4	Q.rob-fr	Google Lens	Quercus robur	Woody	Fruit	100	100			Q.robur				
330	5	Q.rob-fr	Google Lens	Quercus robur	Woody	Fruit	100	100			Q.robur				
331	1	Q.rob-pl	Google Lens	Quercus robur	Woody	Plant	100	100	4		Q.robur				
332	2	Q.rob-pl	Google Lens	Quercus robur	Woody	Plant	100	100			Q.robur				
333	3	Q.rob-pl	Google Lens	Quercus robur	Woody	Plant	0	25			European ash	Populus	Q.robur		1
334	4	Q.rob-pl	Google Lens	Quercus robur	Woody	Plant	0	25			Sycamore	Horse chestnut	Q.robur		1
335	5	Q.rob-pl	Google Lens	Quercus robur	Woody	Plant	0	20			Elm	Ash	Q.acrantha		1
336	1	E.nigr-fl	Google Lens	Empetrum nigrum	Woody	Flower	100	100	1		E.nigrum				
337	2	E.nigr-fl	Google Lens	Empetrum nigrum	Woody	Flower	100	100			E.nigrum				
338	3	E.nigr-fl	Google Lens	Empetrum nigrum	Woody	Flower	100	100			E.nigrum				
339	4	E.nigr-fl	Google Lens	Empetrum nigrum	Woody	Flower	100	100			E.nigrum				
340	5	E.nigr-fl	Google Lens	Empetrum nigrum	Woody	Flower	100	100			E.nigrum				
341	1	E.nigr-fr	Google Lens	Empetrum nigrum	Woody	Fruit	100	100	3		Crowberry				
342	2	E.nigr-fr	Google Lens	Empetrum nigrum	Woody	Fruit	50	50			Vaccinium myrtillus	Berberis			1
343	3	E.nigr-fr	Google Lens	Empetrum nigrum	Woody	Fruit	0	0			Rapholepis	Lava plum			1
344	4	E.nigr-fr	Google Lens	Empetrum nigrum	Woody	Fruit	100	100			Crowberry	Vaccinium myrtillus			
345	5	E.nigr-fr	Google Lens	Empetrum nigrum	Woody	Fruit	100	100			Crowberry				
346	1	A.pseudo	Google Lens	Acer pseudoplatanus	Woody	Leaf	100	100	1		Sycamore	Acer opalis			
347	2	A.pseudo	Google Lens	Acer pseudoplatanus	Woody	Leaf	100	100			Sycamore	Acer opalis			
348	3	A.pseudo	Google Lens	Acer pseudoplatanus	Woody	Leaf	100	100			Sycamore	Acer opalis			
349	4	A.pseudo	Google Lens	Acer pseudoplatanus	Woody	Leaf	100	100			Sycamore	Acer opalis			
350	5	A.pseudo	Google Lens	Acer pseudoplatanus	Woody	Leaf	100	100			Sycamore	Acer opalis			
351	1	C.pauci	Google Lens	Carex pauciflora	Monocot	Flower	100	100	1		C.pauciflora				
352	2	C.pauci	Google Lens	Carex pauciflora	Monocot	Flower	100	100			C.pauciflora				
353	3	C.pauci	Google Lens	Carex pauciflora	Monocot	Flower	100	100			C.pauciflora				
354	4	C.pauci	Google Lens	Carex pauciflora	Monocot	Flower	100	100			C.pauciflora				
355	5	C.pauci	Google Lens	Carex pauciflora	Monocot	Flower	100	100			C.pauciflora				
356	1	C.Fuscus	Google Lens	Cyperus fuscus	Monocot	Plant	100	100	1		C.fuscus				
357	2	C.Fuscus	Google Lens	Cyperus fuscus	Monocot	Plant	100	100			C.fuscus				
358	3	C.Fuscus	Google Lens	Cyperus fuscus	Monocot	Plant	100	100			C.fuscus				
359	4	C.Fuscus	Google Lens	Cyperus fuscus	Monocot	Plant	100	100			C.fuscus				
360	5	C.Fuscus	Google Lens	Cyperus fuscus	Monocot	Plant	100	100			C.fuscus				
361	1	T.marit	Google Lens	Triglochin maritima	Monocot	Plant	100	100	3.2		Seaside arrowgrass				
362	2	T.marit	Google Lens	Triglochin maritima	Monocot	Plant	90	90			Arrowgrasses				
363	3	T.marit	Google Lens	Triglochin maritima	Monocot	Plant	10	33			Sea plantain	Broadleaf plantain	Sea Arrowgrass		1
364	4	T.marit	Google Lens	Triglochin maritima	Monocot	Plant	0	25			Field horsetail	Lomandra	Sea Arrowgrass		1
365	5	T.marit	Google Lens	Triglochin maritima	Monocot	Plant	10	55			Sea plantain	Sea arrowgrass			1
366	1	Delairia	Google Lens	Delairea odorata	Herb	Plant	-5	-5	5		Brasiliopuntia	Norway maple			1
367	2	Delairia	Google Lens	Delairea odorata	Herb	Plant	100	100		tho' only genus only 1 sp!	D.odorata				
368	3	Delairia	Google Lens	Delairea odorata	Herb	Plant	0	50			Haemanthus	D.odorata			1
369	4	Delairia	Google Lens	Delairea odorata	Herb	Plant	-5	13			Pisonia	Guapara	Neea	D.odorata	1
370	5	Delairia	Google Lens	Delairea odorata	Herb	Plant	0	25			Atriplex	Baccharis	D.odorata		1
371	1	E.bonar	Google Lens	Erigeron bonariensis	Herb	Flower	100	100	1		E.bonariensis				
372	2	E.bonar	Google Lens	Erigeron bonariensis	Herb	Flower	100	100			E.bonariensis				
373	3	E.bonar	Google Lens	Erigeron bonariensis	Herb	Flower	100	100			E.bonariensis				
374	4	E.bonar	Google Lens	Erigeron bonariensis	Herb	Flower	100	100			E.bonariensis				
375	5	E.bonar	Google Lens	Erigeron bonariensis	Herb	Flower	100	100			E.bonariensis				
376	1	M.mosch	Google Lens	Malva moschata	Herb	Flower	100	100	1		Musk mallow				
377	2	M.mosch	Google Lens	Malva moschata	Herb	Flower	100	100			Musk mallow				
378	3	M.mosch	Google Lens	Malva moschata	Herb	Flower	100	100			Musk mallow				
379	4	M.mosch	Google Lens	Malva moschata	Herb	Flower	100	100			Musk mallow				
380	5	M.mosch	Google Lens	Malva moschata	Herb	Flower	100	100			Musk mallow				
381	1	P.lutea	PlantNet	Psuedofumaria lutea	Herb	Plant	100	100	3.2		P.lutea(1.56)				
382	2	P.lutea	PlantNet	Psuedofumaria lutea	Herb	Plant	0	0			Lapsana communis(0.3)				1
383	3	P.lutea	PlantNet	Psuedofumaria lutea	Herb	Plant	0	0			Lapsana(0.42)	Crepis(0.14)	Bryonia		1
384	4	P.lutea	PlantNet	Psuedofumaria lutea	Herb	Plant	100	100			P.lutea(4.57)				
385	5	P.lutea	PlantNet	Psuedofumaria lutea	Herb	Plant	0	50			Primula veris(2.49)	P.lutea(1.28)			1
386	1	D.purp.	PlantNet	Digitalis purpurea	Herb	Leaf	100	100	2		D.purpurea(3.59)				
387	2	D.purp.	PlantNet	Digitalis purpurea	Herb	Leaf	100	100			D.purpurea(3.22)				
388	3	D.purp.	PlantNet	Digitalis purpurea	Herb	Leaf	0	50			Borago(2.88)	D.purpurea(1.36)			1
389	4	D.purp.	PlantNet	Digitalis purpurea	Herb	Leaf	0	50			Borago(1.76)	D.purpurea(1.44)			1
390	5	D.purp.	PlantNet	Digitalis purpurea	Herb	Leaf	0	25			Borago(2.22)	Marrubium((0.76)	D.purpurea(0.35)		1
391	1	S.coron.	PlantNet	Silene coronaria	Herb	Leaf	10	10	2	Stachys/Verbascum both10	Stachys byzantina(3.2)	Verbascum thapsus(0.9)			1
392	2	S.coron.	PlantNet	Silene coronaria	Herb	Leaf	10	10		Stachys/Verbascum both10	Verbascum thapsus(3.54)	S.byzantina(0.36)			1
393	3	S.coron.	PlantNet	Silene coronaria	Herb	Leaf	10	10		Stachys/Verbascum both10	Verbascum thapsus(2.3)	S.byzantina(1.4)			1
394	4	S.coron.	PlantNet	Silene coronaria	Herb	Leaf	10	10		Stachys/Verbascum both10	Verbascum thapsus(1.87)	S.byzantina(1.28)			1
395	5	S.coron.	PlantNet	Silene coronaria	Herb	Leaf	10	10		Stachys/Verbascum both10	S.byzantina(2.18)	Plantago			1
396	1	C.off.	PlantNet	Calendula officinalis	Herb	Plant	100	100	2		C.officinalis(2.32)				
397	2	C.off.	PlantNet	Calendula officinalis	Herb	Plant	100	100			Pot marigold(3.2)				
398	3	C.off.	PlantNet	Calendula officinalis	Herb	Plant	100	100			Pot marigold(3.2)				
399	4	C.off.	PlantNet	Calendula officinalis	Herb	Plant	50	63			Zinnia	Common daisy	Pot marigold		1
400	5	C.off.	PlantNet	Calendula officinalis	Herb	Plant	50	75			Zinnia	Pot marigold			1
401	1	M.erecta	PlantNet	Moenchia erecta	Herb	Flower	50	50	4.2		Cerastium glomeratum	Ornithogallum			1
402	2	M.erecta	PlantNet	Moenchia erecta	Herb	Flower	50	81			Sagina subulata(0.36)	M.erecta(0.27)			1
403	3	M.erecta	PlantNet	Moenchia erecta	Herb	Flower	-5	3			Albizzia(0.13)	Myosotis(0.08)	Stellaria		1
404	4	M.erecta	PlantNet	Moenchia erecta	Herb	Flower	5	44		sl. similrity	Ornithogallum(0.3)	Sagina(0.2)	M.erecta(0.19)		1
405	5	M.erecta	PlantNet	Moenchia erecta	Herb	Flower	0	0			Plantago(0.18)	Holcus(0.12)	Capsella		1
406	1	S.verna	PlantNet	Sabulina verna	Herb	Plant	100	100	2.4	Sabulina=Minuartia	M.verna(1.59)				
407	2	S.verna	PlantNet	Sabulina verna	Herb	Plant	0	0			Poa bulbosa(0.59)	Carex humilis(0.39)			1
408	3	S.verna	PlantNet	Sabulina verna	Herb	Plant	0	0			Mibora(0.86)	Poa(0.59)			1
409	4	S.verna	PlantNet	Sabulina verna	Herb	Plant	90	95			Minuartia recurva((0.82)	M.verna(0.49)			
410	5	S.verna	PlantNet	Sabulina verna	Herb	Plant	100	100			M.verna(1.59)				
411	1	E.marit.	PlantNet	Erodium maritimum	Herb	Leaf	0	0	3		Unknown				
412	2	E.marit.	PlantNet	Erodium maritimum	Herb	Leaf	0	0			Bowlesia(1.32)	Sibthorpia(0.79)			1
413	3	E.marit.	PlantNet	Erodium maritimum	Herb	Leaf	0	0			Glechoma(0.56)	Acer campestre(0.22)			1
414	4	E.marit.	PlantNet	Erodium maritimum	Herb	Leaf	0	0			Glechoma(0.56)	Acer campestre(0.22)			1
415	5	E.marit.	PlantNet	Erodium maritimum	Herb	Leaf	0	0			Bowlesia(1.51)	Adoxa(0.83)			1
416	1	G.purp.	PlantNet	Geranium purpureum	Herb	Plant	90	95	1		G.robertianum(0.55)	G.purpureum(0.2)			
417	2	G.purp.	PlantNet	Geranium purpureum	Herb	Plant	90	90			G.robertianum(0.49)	Epilobium ciliatum(0.42)			
418	3	G.purp.	PlantNet	Geranium purpureum	Herb	Plant	90	93			G.robertianum(0.42)	Epilobium ciliatum(0.212)	G.purpureum		

419	4	G.purp.	PlantNet	Geranium purpureum	Herb	Plant	90	95			G.robertianum(2.97)	G.purpureum(1.62)			
420	5	G.purp.	PlantNet	Geranium purpureum	Herb	Plant	90	95			G.robertianum(3.93)	G.purpureum(0.8)			
421	1	A.diandra	PlantNet	Anisantha diandra	Monocot	Flower	90	93	1.2		A.tectorum	A.sterilis	A.diandra		
422	2	A.diandra	PlantNet	Anisantha diandra	Monocot	Flower	90	92			A.sterilis(1.06)	A.tectorum(0.45)	B.racemosus	A.diandra	
423	3	A.diandra	PlantNet	Anisantha diandra	Monocot	Flower	90	90			A.sterilis(0.96)	Sorghum milliaceum(0.27)	B.arvensis		
424	4	A.diandra	PlantNet	Anisantha diandra	Monocot	Flower	90	90			A.sterilis(1.46)	A.tectorum(0.31)			
425	5	A.diandra	PlantNet	Anisantha diandra	Monocot	Flower	90	95			A.tectorum(1.47)	A.diandra(0.42)			
426	1	S.arv.-lf	PlantNet	Spergula arvensis	Herb	Leaf	100	100	3		S.arvensis(3.2)				
427	2	S.arv.-lf	PlantNet	Spergula arvensis	Herb	Leaf	0	5			Lavandula(0.28)	Equisetum arvense(0.27)			1
428	3	S.arv.-lf	PlantNet	Spergula arvensis	Herb	Leaf	10	5		Equisetum merits 5	Equisetum arvense(1.57)				1
429	4	S.arv.-lf	PlantNet	Spergula arvensis	Herb	Leaf	10	33			Equisetum arvense(0.68)	Ranunculus auricomus().38)	S.arvensis(0.32)		1
430	5	S.arv.-lf	PlantNet	Spergula arvensis	Herb	Leaf	100	100			S.arvensis(4.58)				
431	1	S.arv.-fl	PlantNet	Spergula arvensis	Herb	Flower	0	0	2		Matricaria camomilla(0.95)	Fragaria vesca(0.66)	Anemone		1
432	2	S.arv.-fl	PlantNet	Spergula arvensis	Herb	Flower	100	100			S.arvensis(1.53)				
433	3	S.arv.-fl	PlantNet	Spergula arvensis	Herb	Flower	100	100			S.arvensis(4.87)				
434	4	S.arv.-fl	PlantNet	Spergula arvensis	Herb	Flower	100	100			S.arvensis(3.35)				
435	5	S.arv.-fl	PlantNet	Spergula arvensis	Herb	Flower	100	100			S.arvensis(4.48)				
436	1	S.arv.-pl	PlantNet	Spergula arvensis	Herb	Plant	100	100	1		S.arvensis(3.79)				
437	2	S.arv.-pl	PlantNet	Spergula arvensis	Herb	Plant	100	100			S.arvensis(3.91)				
438	3	S.arv.-pl	PlantNet	Spergula arvensis	Herb	Plant	100	100			S.arvensis(3.77)				
439	4	S.arv.-pl	PlantNet	Spergula arvensis	Herb	Plant	100	100			S.arvensis(4.16)				
440	5	S.arv.-pl	PlantNet	Spergula arvensis	Herb	Plant	100	100			S.arvensis(2.33)				
441	1	S.retic.	PlantNet	Salix reticulata	Woody	Leaf	90	90	1		S.herbacea(0.50)				
442	2	S.retic.	PlantNet	Salix reticulata	Woody	Leaf	90	90			S.herbacea(0.53)				
443	3	S.retic.	PlantNet	Salix reticulata	Woody	Leaf	90	90			S.herbacea(0.48)				
444	4	S.retic.	PlantNet	Salix reticulata	Woody	Leaf	90	90			S.herbacea(1.0)				
445	5	S.retic.	PlantNet	Salix reticulata	Woody	Leaf	90	90			S.herbacea(0.42)				
446	1	A.sylv-fl	PlantNet	Angelica sylvestris	Herb	Flower	100	100	1		A.sylvestris(4.0)				
447	2	A.sylv-fl	PlantNet	Angelica sylvestris	Herb	Flower	100	100			A.sylvestris(4.4)				
448	3	A.sylv-fl	PlantNet	Angelica sylvestris	Herb	Flower	100	100			A.sylvestris(3.4)				
449	4	A.sylv-fl	PlantNet	Angelica sylvestris	Herb	Flower	100	100			A.sylvestris(4.7)				
450	5	A.sylv-fl	PlantNet	Angelica sylvestris	Herb	Flower	100	100			A.sylvestris(1.43)				
451	1	A.sylv-lf	PlantNet	Angelica sylvestris	Herb	Leaf	100	100	1		A.sylvestris(2.20)				
452	2	A.sylv-lf	PlantNet	Angelica sylvestris	Herb	Leaf	100	100			A.sylvestris(3.92)				
453	3	A.sylv-lf	PlantNet	Angelica sylvestris	Herb	Leaf	100	100			A.sylvestris(1.43)				
454	4	A.sylv-lf	PlantNet	Angelica sylvestris	Herb	Leaf	100	100			A.sylvestris(4.92)				
455	5	A.sylv-lf	PlantNet	Angelica sylvestris	Herb	Leaf	100	100			A.sylvestris				
456	1	A.syl-pl	PlantNet	Angelica sylvestris	Herb	Plant	100	100	2.2		A.sylvestris				
457	2	A.syl-pl	PlantNet	Angelica sylvestris	Herb	Plant	0	25			Equisetum hyemale	Daucus	Heracleum		1
458	3	A.syl-pl	PlantNet	Angelica sylvestris	Herb	Plant	100	100			A.sylvestris(3.99)				
459	4	A.syl-pl	PlantNet	Angelica sylvestris	Herb	Plant	100	100			A.sylvestris(1.6)				
460	5	A.syl-pl	PlantNet	Angelica sylvestris	Herb	Plant	50	75			Daucus carota(1.41)	A.sylvestris(1.3)			1
461	1	A.sylv-fr	PlantNet	Angelica sylvestris	Herb	Fruit	-5	-5	3		Acanthus	Irrelevant			1
462	2	A.sylv-fr	PlantNet	Angelica sylvestris	Herb	Fruit	100	100			A.sylvestris				
463	3	A.sylv-fr	PlantNet	Angelica sylvestris	Herb	Fruit	100	100			A.sylvestris				
464	4	A.sylv-fr	PlantNet	Angelica sylvestris	Herb	Fruit	100	100			A.sylvestris				
465	5	A.sylv-fr	PlantNet	Angelica sylvestris	Herb	Fruit	0	50			Allium ampeloprasum	A.sylvestris			1
466	1	A.caucal.-fl	PlantNet	Anthriscus caucalis	Herb	Flower	-5	-5	3.4		Equisetum hyemale	Plantago lanceolata	Equisetum		1
467	2	A.caucal.-fl	PlantNet	Anthriscus caucalis	Herb	Flower	-5	-5			Triticum aestivum	Equisetum hyemale			1
468	3	A.caucal.-fl	PlantNet	Anthriscus caucalis	Herb	Flower	70	85			Torilis arvensis	Anthriscus caucalis			
469	4	A.caucal.-fl	PlantNet	Anthriscus caucalis	Herb	Flower	100	100			Anthriscus caucalis(2.22)				
470	5	A.caucal.-fl	PlantNet	Anthriscus caucalis	Herb	Flower	-5	-5			Equisetum hyemale(0.39)	Equisetum			1
471	1	A.caucal.-pl	PlantNet	Anthriscus caucalis	Herb	Plant	90	90	3.4		Cow parsley	Daucus carota	Scandix		
472	2	A.caucal.-pl	PlantNet	Anthriscus caucalis	Herb	Plant	0	0			Capsella	Saxifraga	Cardamine		1
473	3	A.caucal.-pl	PlantNet	Anthriscus caucalis	Herb	Plant	0	0			Cardamine(1.06)	Saxifraga(0.46)	Cardamine amara		1
474	4	A.caucal.-pl	PlantNet	Anthriscus caucalis	Herb	Plant	0	0			Saxifraga granulata(0.63)	Cardamine(0.36)			1
475	5	A.caucal.-pl	PlantNet	Anthriscus caucalis	Herb	Plant	50	75			Scandix pecten-veneris	A.caucalis			
476	1	A.caucal-lf	PlantNet	Anthriscus caucalis	Herb	Leaf	50	50	3.2		Conium	Heracleum			
477	2	A.caucal-lf	PlantNet	Anthriscus caucalis	Herb	Leaf	50	50			Heracleum mantegazzanium(0.91)	Papaver(0.65)	Thlaspi		1
478	3	A.caucal-lf	PlantNet	Anthriscus caucalis	Herb	Leaf	-5	-5			Equisetum hyemale(1.38)	Equisetum(0.84)			1
479	4	A.caucal-lf	PlantNet	Anthriscus caucalis	Herb	Leaf	-5	-5			Equisetum hyemale(2.2)	Equisetum(0.84)			1
480	5	A.caucal-lf	PlantNet	Anthriscus caucalis	Herb	Leaf	-5	-5			Pteridium aquilinum(0.69)	Papaver(0.49)			1
481	1	H.elod-Fl	PlantNet	Hypericum elodes	Herb	Flower	0	0	3		Lagurus	Irrelevant			1
482	2	H.elod-Fl	PlantNet	Hypericum elodes	Herb	Flower	10	10			Verbascum thapsus(1.94)				1
483	3	H.elod-Fl	PlantNet	Hypericum elodes	Herb	Flower	10	10			Verbascum thapsus(0.94)	Stachys byzantina			1
484	4	H.elod-Fl	PlantNet	Hypericum elodes	Herb	Flower	5	8			Verbascum sinuatum(0.41)	Verbascum thapsus			1
485	5	H.elod-Fl	PlantNet	Hypericum elodes	Herb	Flower	-5	-5			Equisetum arvense				1
486	1	H.elod-lf	PlantNet	Hypericum elodes	Herb	Leaf	-5	-5	5		Cedrus				1
487	2	H.elod-lf	PlantNet	Hypericum elodes	Herb	Leaf	-5	-5			Equisetum hyemale	Fagus			1
488	3	H.elod-lf	PlantNet	Hypericum elodes	Herb	Leaf	10	10			Verbascum pulverentum	Stachys byzantina			1
489	4	H.elod-lf	PlantNet	Hypericum elodes	Herb	Leaf	0	3			Phalaris	Triticum	Stachys byzantina		1
490	5	H.elod-lf	PlantNet	Hypericum elodes	Herb	Leaf	10	10			Stachys byzantina				1
491	1	H.elod-pl	PlantNet	Hypericum elodes	Herb	Plant	100	100	4		H.elodes				
492	2	H.elod-pl	PlantNet	Hypericum elodes	Herb	Plant	10	10			Verbascum thapsus				1
493	3	H.elod-pl	PlantNet	Hypericum elodes	Herb	Plant	-5	-5			Acer(0.4)	Hedera helix			1
494	4	H.elod-pl	PlantNet	Hypericum elodes	Herb	Plant	-5	-5			Quercus robur(0.36)	Equisetum			1
495	5	H.elod-pl	PlantNet	Hypericum elodes	Herb	Plant	10	33			Verbascum thapsus	Cistus	H.elodes		1
496	1	C.remot-fl1	PlantNet	Carex remota	Monocot	Flower	100	100	1		C.remota(3.58)				
497	2	C.remot-fl1	PlantNet	Carex remota	Monocot	Flower	100	100			C.remota(3.2)				
498	3	C.remot-fl1	PlantNet	Carex remota	Monocot	Flower	100	100			C.remota(3.4)				
499	4	C.remot-fl1	PlantNet	Carex remota	Monocot	Flower	100	100			C.remota(1.6)				
500	5	C.remot-fl1	PlantNet	Carex remota	Monocot	Flower	100	100			C.remota				
501	1	C.remot-pl	PlantNet	Carex remota	Monocot	Plant	10	10	2.4	grass/moncot-10;rush20	Holcus	Digitaria			1
502	2	C.remot-pl	PlantNet	Carex remota	Monocot	Plant	80	90		grass/moncot-10;rush20	C.hirta(1.69)	C.remota(0.64)			
503	3	C.remot-pl	PlantNet	Carex remota	Monocot	Plant	-5	-1		grass/moncot-10;rush20	Equisetum hyemale	Equisetum	Holcus		1
504	4	C.remot-pl	PlantNet	Carex remota	Monocot	Plant	80	80		grass/moncot-10;rush20	C.hirta	C.acutiformis			
505	5	C.remot-pl	PlantNet	Carex remota	Monocot	Plant	10	10			Digitaria	Equisetum	Hordeum		1
506	1	C.remot-fl2	PlantNet	Carex remota	Monocot	Flower	10	28	2.6		Juncus effusus(0.54)	Cortaderia	Ammophila	C.divulsa	1
507	2	C.remot-fl2	PlantNet	Carex remota	Monocot	Flower	10	10			Ammophila	Cortaderia			1
508	3	C.remot-fl2	PlantNet	Carex remota	Monocot	Flower	10	10			Cortaderia	Molinia			1
509	4	C.remot-fl2	PlantNet	Carex remota	Monocot	Flower	10	45			Nardus	C.paniculata			1
510	5	C.remot-fl2	PlantNet	Carex remota	Monocot	Flower	10	10			Cortaderia				1
511	1	Q.rob-lf	PlantNet	Quercus robur	Woody	Leaf	100	100	5		Q.robur(4.67)				
512	2	Q.rob-lf	PlantNet	Quercus robur	Woody	Leaf	0	0			Acer platanoides(1.26)	Acer splenium			1
513	3	Q.rob-lf	PlantNet	Quercus robur	Woody	Leaf	-5	-5			Cucurbita(0.25)	Acer			1
514	4	Q.rob-lf	PlantNet	Quercus robur	Woody	Leaf	-5	-5			Asplenium(1.79)	Acer			1
515	5	Q.rob-lf	PlantNet	Quercus robur	Woody	Leaf	-5	-5			Zea(0.29)	Platycerium			1
516	1	Q.rob-fr	PlantNet	Quercus robur	Woody	Fruit	100	100	3		Q.robur(4.85)				
517	2	Q.rob-fr	PlantNet	Quercus robur	Woody	Fruit	100	100			Q.robur(1.76)				
518	3	Q.rob-fr	PlantNet	Quercus robur	Woody	Fruit	0	40			Pyrus calleryana(0.36)	Q.pubescens(0.29)			1
519	4	Q.rob-fr	PlantNet	Quercus robur	Woody	Fruit	100	100			Q.robur(1.16)				
520	5	Q.rob-fr	PlantNet	Quercus robur	Woody	Fruit	0	50			Tilia(0.21)	Q.robur			1
521	1	Q.rob-pl	PlantNet	Quercus robur	Woody	Plant	0	0	5		unknown				
522	2	Q.rob-pl	PlantNet	Quercus robur	Woody	Plant	-5	-5			Abies(0.2)	Fagus			1
523	3	Q.rob-pl	PlantNet	Quercus robur	Woody	Plant	40	40		Score halved as "not found"	Q.rubra(not found)				
524	4	Q.rob-pl	PlantNet	Quercus robur	Woody	Plant	0	20			Fagus(0.37)	Populus(0.25)	Q.rubra		1
525	5	Q.rob-pl	PlantNet	Quercus robur	Woody	Plant	0	20			Populus(0.25)	Crataegus	Q.suber		1
526	1	E.nigr-fl	PlantNet	Empetrum nigrum	Woody	Flower	100	100	2		E.nigrum(1.3)				
527	2	E.nigr-fl	PlantNet	Empetrum nigrum	Woody	Flower	100	100			E.nigrum(1.02)				

528	3	E.nigr-fl	PlantNet	Empetrum nigrum	Woody	Flower	0	50			Sanguisorba(0.27)	E.nigrum(0.64)		1
529	4	E.nigr-fl	PlantNet	Empetrum nigrum	Woody	Flower	100	100			Sanguisorba(1.23)	E.nigrum(0.50)		1
530	5	E.nigr-fl	PlantNet	Empetrum nigrum	Woody	Flower	100	100			E.nigrum(2.04)			
531	1	E.nigr-fr	PlantNet	Empetrum nigrum	Woody	Fruit	100	100	1		E.nigrum(4.18)			
532	2	E.nigr-fr	PlantNet	Empetrum nigrum	Woody	Fruit	100	100			E.nigrum(3.59)			
533	3	E.nigr-fr	PlantNet	Empetrum nigrum	Woody	Fruit	100	100			E.nigrum(2.74)			
534	4	E.nigr-fr	PlantNet	Empetrum nigrum	Woody	Fruit	100	100			E.nigrum(2.35)			
535	5	E.nigr-fr	PlantNet	Empetrum nigrum	Woody	Fruit	100	100			E.nigrum(2.11)			
536	1	A.pseudo	PlantNet	Acer pseudoplatanus	Woody	Leaf	100	100	1		Sycamore(4.09)			
537	2	A.pseudo	PlantNet	Acer pseudoplatanus	Woody	Leaf	100	100			Sycamore(3.6)			
538	3	A.pseudo	PlantNet	Acer pseudoplatanus	Woody	Leaf	100	100			Sycamore(4.21)			
539	4	A.pseudo	PlantNet	Acer pseudoplatanus	Woody	Leaf	100	100			Sycamore(4.45)			
540	5	A.pseudo	PlantNet	Acer pseudoplatanus	Woody	Leaf	100	100			Sycamore(4.05)			
541	1	C.pauci	PlantNet	Carex pauciflora	Monocot	Flower	10	10	4		Lolium perenne	Melica		1
542	2	C.pauci	PlantNet	Carex pauciflora	Monocot	Flower	10	10			Lolium perenne	Elytrigia		1
543	3	C.pauci	PlantNet	Carex pauciflora	Monocot	Flower	10	10			Juncus effusus	Cytisus		1
544	4	C.pauci	PlantNet	Carex pauciflora	Monocot	Flower	50	50			Trichophorum	Lolium perenne		1
545	5	C.pauci	PlantNet	Carex pauciflora	Monocot	Flower	-5	3			Equisetum	Lolium perenne	Juncus	1
546	1	C.Fuscus	PlantNet	Cyperus fuscus	Monocot	Plant	10	30	4.2		Juncus	Carex		1
547	2	C.Fuscus	PlantNet	Cyperus fuscus	Monocot	Plant	0	44			Plantago lanceolata	Bolboschoenus	C.fuscus	1
548	3	C.Fuscus	PlantNet	Cyperus fuscus	Monocot	Plant	-5	48			Pinus	C.fuscus		1
549	4	C.Fuscus	PlantNet	Cyperus fuscus	Monocot	Plant	0	50			Plantago	C.fuscus		1
550	5	C.Fuscus	PlantNet	Cyperus fuscus	Monocot	Plant	100	100			C.fuscus			
551	1	T.marit	PlantNet	Triglochin maritima	Monocot	Plant	100	100	2		T.maritima			
552	2	T.marit	PlantNet	Triglochin maritima	Monocot	Plant	100	100			T.maritima			
553	3	T.marit	PlantNet	Triglochin maritima	Monocot	Plant	0	0			Cuscuta epithemium	Bistorta		1
554	4	T.marit	PlantNet	Triglochin maritima	Monocot	Plant	100	100			T.maritima			
555	5	T.marit	PlantNet	Triglochin maritima	Monocot	Plant	100	100			T.maritima			
556	1	Delairia	PlantNet	Delairea odorata	Herb	Plant	0	0	3		Tilia platyphyllos(0.35)	Liriodendron		1
557	2	Delairia	PlantNet	Delairea odorata	Herb	Plant	0	0			Hedera helix(1.34)	Tilia platyphyllos		1
558	3	Delairia	PlantNet	Delairea odorata	Herb	Plant	0	0			Hedera helix(1.3)	Tilia platyphyllos		1
559	4	Delairia	PlantNet	Delairea odorata	Herb	Plant	0	0			Hedera helix(0.36)	Emex		1
560	5	Delairia	PlantNet	Delairea odorata	Herb	Plant	100	100			d.odorata(3.58)			
561	1	E.bonar	PlantNet	Erigeron bonariensis	Herb	Flower	50	50	2.4		Cyanus segetum(0.92)			1
562	2	E.bonar	PlantNet	Erigeron bonariensis	Herb	Flower	50	65			Crepis foetida(0.73)	E.acer	E.canadensis	1
563	3	E.bonar	PlantNet	Erigeron bonariensis	Herb	Flower	100	100			E.bonariensis(0.76)			
564	4	E.bonar	PlantNet	Erigeron bonariensis	Herb	Flower	100	100			E.bonariensis(0.31)			
565	5	E.bonar	PlantNet	Erigeron bonariensis	Herb	Flower	0	0			Lolium perenne			1
566	1	M.mosch	PlantNet	Malva moschata	Herb	Flower	100	100	1		M.moschata(2.7)			
567	2	M.mosch	PlantNet	Malva moschata	Herb	Flower	100	100			M.moschata(3.14)			
568	3	M.mosch	PlantNet	Malva moschata	Herb	Flower	100	100			M.moschata(2.38)			
569	4	M.mosch	PlantNet	Malva moschata	Herb	Flower	100	100			M.moschata(3.26)			
570	5	M.mosch	PlantNet	Malva moschata	Herb	Flower	100	100			M.moschata(2.75)			
571	1	P.lutea	Seek	Psuedofumaria lutea	Herb	Plant	100	100	1		Yellow corydalis			
572	2	P.lutea	Seek	Psuedofumaria lutea	Herb	Plant	100	100			Yellow corydalis			
573	3	P.lutea	Seek	Psuedofumaria lutea	Herb	Plant	100	100			Yellow corydalis			
574	4	P.lutea	Seek	Psuedofumaria lutea	Herb	Plant	100	100			Yellow corydalis			
575	5	P.lutea	Seek	Psuedofumaria lutea	Herb	Plant	100	100			Yellow corydalis			
576	1	D.purp.	Seek	Digitalis purpurea	Herb	Leaf	100	100	2		Purple foxglove			
577	2	D.purp.	Seek	Digitalis purpurea	Herb	Leaf	0	0			Mints, Plantains, Allies			1
578	3	D.purp.	Seek	Digitalis purpurea	Herb	Leaf	100	100			Purple foxglove			
579	4	D.purp.	Seek	Digitalis purpurea	Herb	Leaf	100	100			Purple foxglove			
580	5	D.purp.	Seek	Digitalis purpurea	Herb	Leaf	0	0			Mints, Plantains, Allies			1
581	1	S.coron.	Seek	Silene coronaria	Herb	Leaf	60	60	1.1	Hovered with wait	Dicots/Rose campion			
582	2	S.coron.	Seek	Silene coronaria	Herb	Leaf	0	0			Dicots			
583	3	S.coron.	Seek	Silene coronaria	Herb	Leaf	0	0			Dicots			
584	4	S.coron.	Seek	Silene coronaria	Herb	Leaf	0	0			Dicots			
585	5	S.coron.	Seek	Silene coronaria	Herb	Leaf	0	0			Dicots			
586	1	C.off.	Seek	Calendula officinalis	Herb	Plant	95	95	1.4	required wait!	Pot marigold			
587	2	C.off.	Seek	Calendula officinalis	Herb	Plant	90	90			Marigolds/Pot marigold			
588	3	C.off.	Seek	Calendula officinalis	Herb	Plant	100	100			Pot marigold			
589	4	C.off.	Seek	Calendula officinalis	Herb	Plant	50	50			Sunflowers etc			
590	5	C.off.	Seek	Calendula officinalis	Herb	Plant	100	100			Pot marigold			
591	1	M.erecta	Seek	Moenchia erecta	Herb	Flower	0	0	2		Dicots			
592	2	M.erecta	Seek	Moenchia erecta	Herb	Flower	0	0			Dicots			
593	3	M.erecta	Seek	Moenchia erecta	Herb	Flower	0	0			Dicots			
594	4	M.erecta	Seek	Moenchia erecta	Herb	Flower	50	50			Pinks			
595	5	M.erecta	Seek	Moenchia erecta	Herb	Flower	50	50			Pinks			
596	1	S.verna	Seek	Sabulina verna	Herb	Plant	50	50	2.1		Pinks			
597	2	S.verna	Seek	Sabulina verna	Herb	Plant	50	50			Pinks			
598	3	S.verna	Seek	Sabulina verna	Herb	Plant	50	50			Pinks			
599	4	S.verna	Seek	Sabulina verna	Herb	Plant	40	40			Pinks cactuses and allies			
600	5	S.verna	Seek	Sabulina verna	Herb	Plant	50	50			Pinks			
601	1	E.marit.	Seek	Erodium maritimum	Herb	Leaf	0	0	2.2		Liverwort			1
602	2	E.marit.	Seek	Erodium maritimum	Herb	Leaf	0	0			Conocephalum			1
603	3	E.marit.	Seek	Erodium maritimum	Herb	Leaf	0	0			Snakewort			1
604	4	E.marit.	Seek	Erodium maritimum	Herb	Leaf	0	0			Sankewort			1
605	5	E.marit.	Seek	Erodium maritimum	Herb	Leaf	0	0			Conocephalum			1
606	1	G.purp.	Seek	Geranium purpureum	Herb	Plant	70	70	1.3		Geraniums&cranesbills			
607	2	G.purp.	Seek	Geranium purpureum	Herb	Plant	70	70			Geraniums&cranesbills			
608	3	G.purp.	Seek	Geranium purpureum	Herb	Plant	70	70			Geraniums&cranesbills			
609	4	G.purp.	Seek	Geranium purpureum	Herb	Plant	50	50			Geranium family			
610	5	G.purp.	Seek	Geranium purpureum	Herb	Plant	100	100			Little robin			
611	1	A.diandra	Seek	Anisantha diandra	Monocot	Flower	95	95	1.6	with wait	Gt Brome			
612	2	A.diandra	Seek	Anisantha diandra	Monocot	Flower	50	50			Grasses			
613	3	A.diandra	Seek	Anisantha diandra	Monocot	Flower	100	100			Gt Brome			
614	4	A.diandra	Seek	Anisantha diandra	Monocot	Flower	80	80			Brome			
615	5	A.diandra	Seek	Anisantha diandra	Monocot	Flower	80	80			Brome			
616	1	S.arv.-lf	Seek	Spergula arvensis	Herb	Leaf	100	100	1.3		Corn spurrey			
617	2	S.arv.-lf	Seek	Spergula arvensis	Herb	Leaf	100	100			Corn spurrey			
618	3	S.arv.-lf	Seek	Spergula arvensis	Herb	Leaf	50	50			Pinks			
619	4	S.arv.-lf	Seek	Spergula arvensis	Herb	Leaf	100	100			Corn spurrey			
620	5	S.arv.-lf	Seek	Spergula arvensis	Herb	Leaf	80	80		wavering	Dicot/Corn spurrey			
621	1	S.arv.-fl	Seek	Spergula arvensis	Herb	Flower	100	100	1		Corn spurrey			
622	2	S.arv.-fl	Seek	Spergula arvensis	Herb	Flower	100	100			Corn spurrey			
623	3	S.arv.-fl	Seek	Spergula arvensis	Herb	Flower	100	100			Corn spurrey			
624	4	S.arv.-fl	Seek	Spergula arvensis	Herb	Flower	100	100			Corn spurrey			
625	5	S.arv.-fl	Seek	Spergula arvensis	Herb	Flower	100	100			Corn spurrey			
626	1	S.arv.-pl	Seek	Spergula arvensis	Herb	Plant	100	100	1		Corn spurrey			
627	2	S.arv.-pl	Seek	Spergula arvensis	Herb	Plant	100	100			Corn spurrey			
628	3	S.arv.-pl	Seek	Spergula arvensis	Herb	Plant	100	100			Corn spurrey			
629	4	S.arv.-pl	Seek	Spergula arvensis	Herb	Plant	100	100			Corn spurrey			
630	5	S.arv.-pl	Seek	Spergula arvensis	Herb	Plant	100	100			Corn spurrey			
631	1	S.retic.	Seek	Salix reticulata	Woody	Leaf	90	90	1.1	wavering(=.1variati on)	Dicot/Net-leaved willow			
632	2	S.retic.	Seek	Salix reticulata	Woody	Leaf	100	100			Net-leaved willow			
633	3	S.retic.	Seek	Salix reticulata	Woody	Leaf	100	100			Net-leaved willow			
634	4	S.retic.	Seek	Salix reticulata	Woody	Leaf	100	100			Net-leaved willow			
635	5	S.retic.	Seek	Salix reticulata	Woody	Leaf	100	100			Net-leaved willow			
636	1	A.sylv-fl	Seek	Angelica sylvestris	Herb	Flower	100	100	1		Wild angelica			
637	2	A.sylv-fl	Seek	Angelica sylvestris	Herb	Flower	100	100			Wild angelica			
638	3	A.sylv-fl	Seek	Angelica sylvestris	Herb	Flower	100	100			Wild angelica			
639	4	A.sylv-fl	Seek	Angelica sylvestris	Herb	Flower	100	100			Wild angelica			
640	5	A.sylv-fl	Seek	Angelica sylvestris	Herb	Flower	100	100			Wild angelica			
641	1	A.sylv-lf	Seek	Angelica sylvestris	Herb	Leaf	100	100	1.3		Wild angelica			
642	2	A.sylv-lf	Seek	Angelica sylvestris	Herb	Leaf	100	100			Wild angelica			
643	3	A.sylv-lf	Seek	Angelica sylvestris	Herb	Leaf	50	50			Carrot family			
644	4	A.sylv-lf	Seek	Angelica sylvestris	Herb	Leaf	80	80			Angelica			
645	5	A.sylv-lf	Seek	Angelica sylvestris	Herb	Leaf	50	50			Carrot family			
646	1	A.syl-pl	Seek	Angelica sylvestris	Herb	Plant	100	100	1		Wild angelica			
647	2	A.syl-pl	Seek	Angelica sylvestris	Herb	Plant	100	100			Wild angelica			
648	3	A.syl-pl	Seek	Angelica sylvestris	Herb	Plant	100	100			Wild angelica			
649	4	A.syl-pl	Seek	Angelica sylvestris	Herb	Plant	100	100			Wild angelica			
650	5	A.syl-pl	Seek	Angelica sylvestris	Herb	Plant	100	100			Wild angelica			

651	1	A.sylv-fr	Seek	Angelica sylvestris	Herb	Fruit	50	50	1.4		Carrot family		
652	2	A.sylv-fr	Seek	Angelica sylvestris	Herb	Fruit	80	80			Angelica		
653	3	A.sylv-fr	Seek	Angelica sylvestris	Herb	Fruit	100	100			Wild angelica		
654	4	A.sylv-fr	Seek	Angelica sylvestris	Herb	Fruit	50	50			Carrot family		
655	5	A.sylv-fr	Seek	Angelica sylvestris	Herb	Fruit	80	80			Angelica		
656	1	A.caucal.-fl	Seek	Anthriscus caucalis	Herb	Flower	85	85	1	NB usually refers to Caucalis platyphyllos, but rarely A.caucalis	Bur parsley		
657	2	A.caucal.-fl	Seek	Anthriscus caucalis	Herb	Flower	85	85			Bur parsley		
658	3	A.caucal.-fl	Seek	Anthriscus caucalis	Herb	Flower	85	85			Bur parsley		
659	4	A.caucal.-fl	Seek	Anthriscus caucalis	Herb	Flower	85	85			Bur parsley		
660	5	A.caucal.-fl	Seek	Anthriscus caucalis	Herb	Flower	85	85			Bur parsley		
661	1	A.caucal.-pl	Seek	Anthriscus caucalis	Herb	Plant	85	85	1.3		Bur parsley		
662	2	A.caucal.-pl	Seek	Anthriscus caucalis	Herb	Plant	85	85			Bur parsley		
663	3	A.caucal.-pl	Seek	Anthriscus caucalis	Herb	Plant	85	85			Bur parsley		
664	4	A.caucal.-pl	Seek	Anthriscus caucalis	Herb	Plant	50	50			Carrot family		
665	5	A.caucal.-pl	Seek	Anthriscus caucalis	Herb	Plant	80	80		wavering	Dicots/Bur parsley		
666	1	A.caucal-lf	Seek	Anthriscus caucalis	Herb	Leaf	-5	-5	2		Conifers		1
667	2	A.caucal-lf	Seek	Anthriscus caucalis	Herb	Leaf	-5	-5			Polypodiales		1
668	3	A.caucal-lf	Seek	Anthriscus caucalis	Herb	Leaf	-5	-5			Conifers		1
669	4	A.caucal-lf	Seek	Anthriscus caucalis	Herb	Leaf	-5	-5			Conifers		1
670	5	A.caucal-lf	Seek	Anthriscus caucalis	Herb	Leaf	-5	-5			Conifers		1
671	1	H.elod-Fl	Seek	Hypericum elodes	Herb	Flower	0	0	2		Dicots		
672	2	H.elod-Fl	Seek	Hypericum elodes	Herb	Flower	-5	-5			Butterflies		1
673	3	H.elod-Fl	Seek	Hypericum elodes	Herb	Flower	0	0			Dicots		
674	4	H.elod-Fl	Seek	Hypericum elodes	Herb	Flower	0	0			Dicots		
675	5	H.elod-Fl	Seek	Hypericum elodes	Herb	Flower	0	0			Dicots		
676	1	H.elod-lf	Seek	Hypericum elodes	Herb	Leaf	10	10	3		lamb's ear		1
677	2	H.elod-lf	Seek	Hypericum elodes	Herb	Leaf	0	0			Dicots		
678	3	H.elod-lf	Seek	Hypericum elodes	Herb	Leaf	10	10			Hedgenettle genus		1
679	4	H.elod-lf	Seek	Hypericum elodes	Herb	Leaf	0	0			Dicots		
680	5	H.elod-lf	Seek	Hypericum elodes	Herb	Leaf	0	0			Dicots		
681	1	H.elod-pl	Seek	Hypericum elodes	Herb	Plant	0	0	1.1		Dicots		
682	2	H.elod-pl	Seek	Hypericum elodes	Herb	Plant	60	60		hovered with wait	Dicots/H.elodes		
683	3	H.elod-pl	Seek	Hypericum elodes	Herb	Plant	0	0			Dicots		
684	4	H.elod-pl	Seek	Hypericum elodes	Herb	Plant	0	0			Dicots		
685	5	H.elod-pl	Seek	Hypericum elodes	Herb	Plant	100	100			H.elodes		
686	1	C.remot-fl1	Seek	Carex remota	Monocot	Flower	80	80	2.1		True sedges		
687	2	C.remot-fl1	Seek	Carex remota	Monocot	Flower	80	80			Carex		
688	3	C.remot-fl1	Seek	Carex remota	Monocot	Flower	40	40			Grasses/sedges		
689	4	C.remot-fl1	Seek	Carex remota	Monocot	Flower	80	80			True sedges		
690	5	C.remot-fl1	Seek	Carex remota	Monocot	Flower	80	80			True sedges		
691	1	C.remot-pl	Seek	Carex remota	Monocot	Plant	80	80	3.1		True sedges		
692	2	C.remot-pl	Seek	Carex remota	Monocot	Plant	40	40			Grasses/sedges		
693	3	C.remot-pl	Seek	Carex remota	Monocot	Plant	20	20			Brazilian vervain		1
694	4	C.remot-pl	Seek	Carex remota	Monocot	Plant	50	50			Sedges		
695	5	C.remot-pl	Seek	Carex remota	Monocot	Plant	40	40			Grasses/sedges		
696	1	C.remot-fl2	Seek	Carex remota	Monocot	Flower	20	20	2.1		Grasses		1
697	2	C.remot-fl2	Seek	Carex remota	Monocot	Flower	40	40			Grasses/sedges		
698	3	C.remot-fl2	Seek	Carex remota	Monocot	Flower	20	20			Brazilian vervain		1
699	4	C.remot-fl2	Seek	Carex remota	Monocot	Flower	20	20			Grasses		1
700	5	C.remot-fl2	Seek	Carex remota	Monocot	Flower	20	20			Grasses		1
701	1	Q.rob-lf	Seek	Quercus robur	Woody	Leaf	80	80	1.2		Quercus		
702	2	Q.rob-lf	Seek	Quercus robur	Woody	Leaf	80	80			Post oak		
703	3	Q.rob-lf	Seek	Quercus robur	Woody	Leaf	80	80			Quercus		
704	4	Q.rob-lf	Seek	Quercus robur	Woody	Leaf	80	80			Quercus		
705	5	Q.rob-lf	Seek	Quercus robur	Woody	Leaf	80	80			Quercus		
706	1	Q.rob-fr	Seek	Quercus robur	Woody	Fruit	100	100			Q.robur		
707	2	Q.rob-fr	Seek	Quercus robur	Woody	Fruit	80	80	1.2		Oaks		
708	3	Q.rob-fr	Seek	Quercus robur	Woody	Fruit	80	80			Oaks		
709	4	Q.rob-fr	Seek	Quercus robur	Woody	Fruit	80	80			Oaks		
710	5	Q.rob-fr	Seek	Quercus robur	Woody	Fruit	80	80			Oaks		
711	1	Q.rob-pl	Seek	Quercus robur	Woody	Plant	0	0	1		Dicots		
712	2	Q.rob-pl	Seek	Quercus robur	Woody	Plant	0	0			Dicots		
713	3	Q.rob-pl	Seek	Quercus robur	Woody	Plant	0	0			Dicots		
714	4	Q.rob-pl	Seek	Quercus robur	Woody	Plant	0	0			Dicots		
715	5	Q.rob-pl	Seek	Quercus robur	Woody	Plant	0	0			Dicots		
716	1	E.nigr-fl	Seek	Empetrum nigrum	Woody	Flower	0	0	2		Dicots		
717	2	E.nigr-fl	Seek	Empetrum nigrum	Woody	Flower	0	0			Dicots		
718	3	E.nigr-fl	Seek	Empetrum nigrum	Woody	Flower	0	0			Dicots		
719	4	E.nigr-fl	Seek	Empetrum nigrum	Woody	Flower	0	0			Dicots		
720	5	E.nigr-fl	Seek	Empetrum nigrum	Woody	Flower	-5	-5			Maize		1
721	1	E.nigr-fr	Seek	Empetrum nigrum	Woody	Fruit	100	100	2		Black crowberry		
722	2	E.nigr-fr	Seek	Empetrum nigrum	Woody	Fruit	50	50			Heathers		1
723	3	E.nigr-fr	Seek	Empetrum nigrum	Woody	Fruit	100	100			Black crowberry		
724	4	E.nigr-fr	Seek	Empetrum nigrum	Woody	Fruit	50	50			Heathers		1
725	5	E.nigr-fr	Seek	Empetrum nigrum	Woody	Fruit	100	100			Black crowberry		
726	1	A.pseudo	Seek	Acer pseudoplatanus	Woody	Leaf	100	100	1		Sycamore		
727	2	A.pseudo	Seek	Acer pseudoplatanus	Woody	Leaf	100	100			Sycamore		
728	3	A.pseudo	Seek	Acer pseudoplatanus	Woody	Leaf	100	100			Sycamore		
729	4	A.pseudo	Seek	Acer pseudoplatanus	Woody	Leaf	100	100			Sycamore		
730	5	A.pseudo	Seek	Acer pseudoplatanus	Woody	Leaf	100	100			Sycamore		
731	1	C.pauci	Seek	Carex pauciflora	Monocot	Flower	0	0	4		None		
732	2	C.pauci	Seek	Carex pauciflora	Monocot	Flower	10	10			Moncots		
733	3	C.pauci	Seek	Carex pauciflora	Monocot	Flower	-5	-5			Insects		1
734	4	C.pauci	Seek	Carex pauciflora	Monocot	Flower	40	40			Grasses,sedges,etc		
735	5	C.pauci	Seek	Carex pauciflora	Monocot	Flower	0	0			None		
736	1	C.Fuscus	Seek	Cyperus fuscus	Monocot	Plant	50	50	2		Sedges		
737	2	C.Fuscus	Seek	Cyperus fuscus	Monocot	Plant	50	50			Sedges		
738	3	C.Fuscus	Seek	Cyperus fuscus	Monocot	Plant	40	40			Grasses, sedges etc		
739	4	C.Fuscus	Seek	Cyperus fuscus	Monocot	Plant	50	50			Sedges		
740	5	C.Fuscus	Seek	Cyperus fuscus	Monocot	Plant	40	40			Grasses, sedges, etc.		
741	1	T.marit	Seek	Triglochin maritima	Monocot	Plant	100	100	2		Common arrowgrass		
742	2	T.marit	Seek	Triglochin maritima	Monocot	Plant	100	100			Common arrowgrass		
743	3	T.marit	Seek	Triglochin maritima	Monocot	Plant	100	100			Common arrowgrass		
744	4	T.marit	Seek	Triglochin maritima	Monocot	Plant	10	10			Monocot		
745	5	T.marit	Seek	Triglochin maritima	Monocot	Plant	100	100			Common arrowgrass		
746	1	Delairia	Seek	Delairea odorata	Herb	Plant	100	100	1		Cape ivy		
747	2	Delairia	Seek	Delairea odorata	Herb	Plant	100	100			Cape ivy		
748	3	Delairia	Seek	Delairea odorata	Herb	Plant	100	100			Cape ivy		
749	4	Delairia	Seek	Delairea odorata	Herb	Plant	100	100			Cape ivy		
750	5	Delairia	Seek	Delairea odorata	Herb	Plant	100	100			Cape ivy		
751	1	E.bonar	Seek	Erigeron bonariensis	Herb	Flower	80	80	1		Fleabanes etc		
752	2	E.bonar	Seek	Erigeron bonariensis	Herb	Flower	80	80			Fleabanes etc		
753	3	E.bonar	Seek	Erigeron bonariensis	Herb	Flower	80	80			Fleabanes etc		
754	4	E.bonar	Seek	Erigeron bonariensis	Herb	Flower	80	80			Fleabanes etc		
755	5	E.bonar	Seek	Erigeron bonariensis	Herb	Flower	80	80			Fleabanes etc		
756	1	M.mosch	Seek	Malva moschata	Herb	Flower	100	100	1		Musk mallow		
757	2	M.mosch	Seek	Malva moschata	Herb	Flower	100	100			Musk mallow		
758	3	M.mosch	Seek	Malva moschata	Herb	Flower	100	100			Musk mallow		
759	4	M.mosch	Seek	Malva moschata	Herb	Flower	100	100			Musk mallow		
760	5	M.mosch	Seek	Malva moschata	Herb	Flower	100	100			Musk mallow		
761	1	P.lutea	Flora Incognita	Psuedofumaria lutea	Herb	Plant	100	100	1		P.lutea		
762	2	P.lutea	Flora Incognita	Psuedofumaria lutea	Herb	Plant	90	95			P.alba	P.lutea	
763	3	P.lutea	Flora Incognita	Psuedofumaria lutea	Herb	Plant	100	100			P.lutea		
764	4	P.lutea	Flora Incognita	Psuedofumaria lutea	Herb	Plant	100	100			P.lutea		
765	5	P.lutea	Flora Incognita	Psuedofumaria lutea	Herb	Plant	90	95			P.alba	P.lutea	
766	1	D.purp.	Flora Incognita	Digitalis purpurea	Herb	Leaf	0	0	3		unknown		
767	2	D.purp.	Flora Incognita	Digitalis purpurea	Herb	Leaf	0	0			Borage		1
768	3	D.purp.	Flora Incognita	Digitalis purpurea	Herb	Leaf	100	100			Foxglove		
769	4	D.purp.	Flora Incognita	Digitalis purpurea	Herb	Leaf	100	100			Focglove		
770	5	D.purp.	Flora Incognita	Digitalis purpurea	Herb	Leaf	100	100			Focglove		
771	1	S.coron.	Flora Incognita	Silene coronaria	Herb	Leaf	-5	-5	3		Nake plant	Agave	1

772	2	S.coron.	Flora Incognita	Silene coronaria	Herb	Leaf	0	0			unknown		
773	3	S.coron.	Flora Incognita	Silene coronaria	Herb	Leaf	-5	-5			Foxtail agave		1
774	4	S.coron.	Flora Incognita	Silene coronaria	Herb	Leaf	-5	-5			Foxtail agave		1
775	5	S.coron.	Flora Incognita	Silene coronaria	Herb	Leaf	0	0			unknown		
776	1	C.off.	Flora Incognita	Calendula officinalis	Herb	Plant	50	50	2		unknown		
777	2	C.off.	Flora Incognita	Calendula officinalis	Herb	Plant	50	50			Coreopsis		1
778	3	C.off.	Flora Incognita	Calendula officinalis	Herb	Plant	50	50			Coreopsis(85%)		1
779	4	C.off.	Flora Incognita	Calendula officinalis	Herb	Plant	50	50			Coreopsis(85%)		1
780	5	C.off.	Flora Incognita	Calendula officinalis	Herb	Plant	50	50			Coreopsis		1
781	1	M.erecta	Flora Incognita	Moenchia erecta	Herb	Flower	100	100	1		M.erecta(93%)		
782	2	M.erecta	Flora Incognita	Moenchia erecta	Herb	Flower	100	100			M.erecta(84%)		
783	3	M.erecta	Flora Incognita	Moenchia erecta	Herb	Flower	100	100			M.erecta(99%)		
784	4	M.erecta	Flora Incognita	Moenchia erecta	Herb	Flower	100	100			M.erecta(84%)		
785	5	M.erecta	Flora Incognita	Moenchia erecta	Herb	Flower	100	100			M.erecta(99%)		
786	1	S.verna	Flora Incognita	Sabulina verna	Herb	Plant	100	100	2		M.verna(93%)		
787	2	S.verna	Flora Incognita	Sabulina verna	Herb	Plant	100	100			M.verna(93%)		
788	3	S.verna	Flora Incognita	Sabulina verna	Herb	Plant	100	100			M.verna(93%)		
789	4	S.verna	Flora Incognita	Sabulina verna	Herb	Plant	-5	-5			Mariposa lily		1
790	5	S.verna	Flora Incognita	Sabulina verna	Herb	Plant	100	100			M.verna(99%)		
791	1	E.marit.	Flora Incognita	Erodium maritimum	Herb	Leaf	-5	-5	4		Azolla		1
792	2	E.marit.	Flora Incognita	Erodium maritimum	Herb	Leaf	-5	-5			Silkfloss tree		1
793	3	E.marit.	Flora Incognita	Erodium maritimum	Herb	Leaf	-5	-5			Maidenhair		1
794	4	E.marit.	Flora Incognita	Erodium maritimum	Herb	Leaf	0	0			unknown		
795	5	E.marit.	Flora Incognita	Erodium maritimum	Herb	Leaf	0	0			unknown		
796	1	G.purp.	Flora Incognita	Geranium purpureum	Herb	Plant	0	0	3		unknown		
797	2	G.purp.	Flora Incognita	Geranium purpureum	Herb	Plant	90	90			G.robertianum(92%)		
798	3	G.purp.	Flora Incognita	Geranium purpureum	Herb	Plant	0	0			unknown		
799	4	G.purp.	Flora Incognita	Geranium purpureum	Herb	Plant	-5	-5			Bird's-foot violet		1
800	5	G.purp.	Flora Incognita	Geranium purpureum	Herb	Plant	90	90			G.robertianum(99%)		
801	1	A.diandra	Flora Incognita	Anisantha diandra	Monocot	Flower	100	100	1		Gt. Brome		
802	2	A.diandra	Flora Incognita	Anisantha diandra	Monocot	Flower	100	100			B.diandrus(99%)		
803	3	A.diandra	Flora Incognita	Anisantha diandra	Monocot	Flower	100	100			B.diandrus(98%)		
804	4	A.diandra	Flora Incognita	Anisantha diandra	Monocot	Flower	100	100			B.diandrus(98%)		
805	5	A.diandra	Flora Incognita	Anisantha diandra	Monocot	Flower	100	100			B.diandrus(96%)		
806	1	S.arv.-lf	Flora Incognita	Spergula arvensis	Herb	Leaf	100	100	3		Corn spurrey		
807	2	S.arv.-lf	Flora Incognita	Spergula arvensis	Herb	Leaf	-5	-5			Pencil tree		1
808	3	S.arv.-lf	Flora Incognita	Spergula arvensis	Herb	Leaf	0	0			unknown		
809	4	S.arv.-lf	Flora Incognita	Spergula arvensis	Herb	Leaf	-5	-5			Pencil tree		1
810	5	S.arv.-lf	Flora Incognita	Spergula arvensis	Herb	Leaf	0	0			unknown		
811	1	S.arv.-fl	Flora Incognita	Spergula arvensis	Herb	Flower	100	100	2.2		Corn spurrey		
812	2	S.arv.-fl	Flora Incognita	Spergula arvensis	Herb	Flower	100	100			Corn spurrey		
813	3	S.arv.-fl	Flora Incognita	Spergula arvensis	Herb	Flower	75	75			Starwort mouseear		1
814	4	S.arv.-fl	Flora Incognita	Spergula arvensis	Herb	Flower	100	100			Corn spurrey(87%)		
815	5	S.arv.-fl	Flora Incognita	Spergula arvensis	Herb	Flower	0	0			unknown		
816	1	S.arv.-pl	Flora Incognita	Spergula arvensis	Herb	Plant	50	50	1.6		Minuartia austriaca		1
817	2	S.arv.-pl	Flora Incognita	Spergula arvensis	Herb	Plant	50	50			Minuartia verna	Sagina nodosa	1
818	3	S.arv.-pl	Flora Incognita	Spergula arvensis	Herb	Plant	100	100			S.arvensis(98%)		
819	4	S.arv.-pl	Flora Incognita	Spergula arvensis	Herb	Plant	100	100			Corn spurrey		
820	5	S.arv.-pl	Flora Incognita	Spergula arvensis	Herb	Plant	50	50			Minuartia verna	Sagina nodosa	1
821	1	S.retic.	Flora Incognita	Salix reticulata	Woody	Leaf	90	90	2		Willow		
822	2	S.retic.	Flora Incognita	Salix reticulata	Woody	Leaf	100	100			Net-leaved willow(95%)		
823	3	S.retic.	Flora Incognita	Salix reticulata	Woody	Leaf	100	100			Net-leaved willow(96%)		
824	4	S.retic.	Flora Incognita	Salix reticulata	Woody	Leaf	100	100			Net-leaved willow(93%)		
825	5	S.retic.	Flora Incognita	Salix reticulata	Woody	Leaf	100	100			Net-leaved willow(91%)		
826	1	A.sylv-fl	Flora Incognita	Angelica sylvestris	Herb	Flower	100	100	1		Wild angelica		
827	2	A.sylv-fl	Flora Incognita	Angelica sylvestris	Herb	Flower	100	100			Wild angelica		
828	3	A.sylv-fl	Flora Incognita	Angelica sylvestris	Herb	Flower	100	100			Wild angelica		
829	4	A.sylv-fl	Flora Incognita	Angelica sylvestris	Herb	Flower	100	100			Wild angelica		
830	5	A.sylv-fl	Flora Incognita	Angelica sylvestris	Herb	Flower	100	100			Wild angelica		
831	1	A.sylv-lf	Flora Incognita	Angelica sylvestris	Herb	Leaf	100	100	1		Wild angelica		
832	2	A.sylv-lf	Flora Incognita	Angelica sylvestris	Herb	Leaf	100	100			Wild angelica		
833	3	A.sylv-lf	Flora Incognita	Angelica sylvestris	Herb	Leaf	100	100			Wild angelica		
834	4	A.sylv-lf	Flora Incognita	Angelica sylvestris	Herb	Leaf	100	100			Wild angelica		
835	5	A.sylv-lf	Flora Incognita	Angelica sylvestris	Herb	Leaf	100	100			Wild angelica		
836	1	A.syl-pl	Flora Incognita	Angelica sylvestris	Herb	Plant	100	100	2		Wild angelica		
837	2	A.syl-pl	Flora Incognita	Angelica sylvestris	Herb	Plant	0	0			Miscanthus		1
838	3	A.syl-pl	Flora Incognita	Angelica sylvestris	Herb	Plant	100	100			Wild angelica		
839	4	A.syl-pl	Flora Incognita	Angelica sylvestris	Herb	Plant	100	100			Wild angelica		
840	5	A.syl-pl	Flora Incognita	Angelica sylvestris	Herb	Plant	100	100			Wild angelica		
841	1	A.sylv-fr	Flora Incognita	Angelica sylvestris	Herb	Fruit	100	100	4		Wild angelica		
842	2	A.sylv-fr	Flora Incognita	Angelica sylvestris	Herb	Fruit	0	0			unknown		
843	3	A.sylv-fr	Flora Incognita	Angelica sylvestris	Herb	Fruit	-5	-5			Water horsetail		1
844	4	A.sylv-fr	Flora Incognita	Angelica sylvestris	Herb	Fruit	0	0			unknown		
845	5	A.sylv-fr	Flora Incognita	Angelica sylvestris	Herb	Fruit	0	0			Holcus mollis		1
846	1	A.caucal.-fl	Flora Incognita	Anthriscus caucalis	Herb	Flower	0	0	2		unknown		
847	2	A.caucal.-fl	Flora Incognita	Anthriscus caucalis	Herb	Flower	0	0			unknown		
848	3	A.caucal.-fl	Flora Incognita	Anthriscus caucalis	Herb	Flower	0	0			unknown		
849	4	A.caucal.-fl	Flora Incognita	Anthriscus caucalis	Herb	Flower	100	100			A.caucalis		
850	5	A.caucal.-fl	Flora Incognita	Anthriscus caucalis	Herb	Flower	100	100			A.caucalis		
851	1	A.caucal.-pl	Flora Incognita	Anthriscus caucalis	Herb	Plant	-5	-5	3		Elodea canadensis		1
852	2	A.caucal.-pl	Flora Incognita	Anthriscus caucalis	Herb	Plant	-5	-5			Elodea canadensis		1
853	3	A.caucal.-pl	Flora Incognita	Anthriscus caucalis	Herb	Plant	-5	-5			Elodea canadensis	Elodea densa	1
854	4	A.caucal.-pl	Flora Incognita	Anthriscus caucalis	Herb	Plant	0	0			unknown		
855	5	A.caucal.-pl	Flora Incognita	Anthriscus caucalis	Herb	Plant	70	70			Scandix pecten-veneris		1
856	1	A.caucal-lf	Flora Incognita	Anthriscus caucalis	Herb	Leaf	85	85	3		Bur chervil		
857	2	A.caucal-lf	Flora Incognita	Anthriscus caucalis	Herb	Leaf	5	5			Urtica urens		1
858	3	A.caucal-lf	Flora Incognita	Anthriscus caucalis	Herb	Leaf	0	0			unknown		
859	4	A.caucal-lf	Flora Incognita	Anthriscus caucalis	Herb	Leaf	0	0			unknown		
860	5	A.caucal-lf	Flora Incognita	Anthriscus caucalis	Herb	Leaf	0	0			unknown		
861	1	H.elod-Fl	Flora Incognita	Hypericum elodes	Herb	Flower	0	0	2		unknown		
862	2	H.elod-Fl	Flora Incognita	Hypericum elodes	Herb	Flower	70	70		works with 3pics(penalty)	H.elodes		
863	3	H.elod-Fl	Flora Incognita	Hypericum elodes	Herb	Flower	70	70		works with 3pics(penalty)	H.elodes		
864	4	H.elod-Fl	Flora Incognita	Hypericum elodes	Herb	Flower	0	0			unknown		
865	5	H.elod-Fl	Flora Incognita	Hypericum elodes	Herb	Flower	0	0			unknown		
866	1	H.elod-lf	Flora Incognita	Hypericum elodes	Herb	Leaf	0	0	1		unknown		
867	2	H.elod-lf	Flora Incognita	Hypericum elodes	Herb	Leaf	0	0			unknown		
868	3	H.elod-lf	Flora Incognita	Hypericum elodes	Herb	Leaf	0	0			unknown		
869	4	H.elod-lf	Flora Incognita	Hypericum elodes	Herb	Leaf	0	0			unknown		
870	5	H.elod-lf	Flora Incognita	Hypericum elodes	Herb	Leaf	0	0			unknown		
871	1	H.elod-pl	Flora Incognita	Hypericum elodes	Herb	Plant	0	0	1		unknown		
872	2	H.elod-pl	Flora Incognita	Hypericum elodes	Herb	Plant	0	0			unknown		
873	3	H.elod-pl	Flora Incognita	Hypericum elodes	Herb	Plant	0	0			unknown		
874	4	H.elod-pl	Flora Incognita	Hypericum elodes	Herb	Plant	0	0			unknown		
875	5	H.elod-pl	Flora Incognita	Hypericum elodes	Herb	Plant	0	0			unknown		
876	1	C.remot-fl1	Flora Incognita	Carex remota	Monocot	Flower	100	100	1		C.remota		
877	2	C.remot-fl1	Flora Incognita	Carex remota	Monocot	Flower	100	100			C.remota(82%)		
878	3	C.remot-fl1	Flora Incognita	Carex remota	Monocot	Flower	100	100			C.remota		
879	4	C.remot-fl1	Flora Incognita	Carex remota	Monocot	Flower	100	100			C.remota(89%)		
880	5	C.remot-fl1	Flora Incognita	Carex remota	Monocot	Flower	100	100			C.remota		
881	1	C.remot-pl	Flora Incognita	Carex remota	Monocot	Plant	10	10	3.2		Tall fescue		1
882	2	C.remot-pl	Flora Incognita	Carex remota	Monocot	Plant	0	0			unknown		
883	3	C.remot-pl	Flora Incognita	Carex remota	Monocot	Plant	0	0			unknown		
884	4	C.remot-pl	Flora Incognita	Carex remota	Monocot	Plant	10	10			Phleum pratense		1
885	5	C.remot-pl	Flora Incognita	Carex remota	Monocot	Plant	50	50			C.hordelostycha		
886	1	C.remot-fl2	Flora Incognita	Carex remota	Monocot	Flower	50	50	2.4		Deer grass		1
887	2	C.remot-fl2	Flora Incognita	Carex remota	Monocot	Flower	10	10			Juncus inflexus	Juncus filiformis	1
888	3	C.remot-fl2	Flora Incognita	Carex remota	Monocot	Flower	10	10			Festuca alpina		1
889	4	C.remot-fl2	Flora Incognita	Carex remota	Monocot	Flower	10	10			Festuca amythestina		1
890	5	C.remot-fl2	Flora Incognita	Carex remota	Monocot	Flower	10	10			Festuca amythestina		1
891	1	Q.rob-lf	Flora Incognita	Quercus robur	Woody	Leaf	100	100	1.2		Q.robur		
892	2	Q.rob-lf	Flora Incognita	Quercus robur	Woody	Leaf	90	95			Q.petraea	Q.robur	
893	3	Q.rob-lf	Flora Incognita	Quercus robur	Woody	Leaf	90	95			Q.petraea	Q.robur	

894	4	Q.rob-lf	Flora Incognita	Quercus robur	Woody	Leaf	100	100			Q.robur				
895	5	Q.rob-lf	Flora Incognita	Quercus robur	Woody	Leaf	100	100			Q.robur				
896	1	Q.rob-fr	Flora Incognita	Quercus robur	Woody	Fruit	100	100			Q.robur				
897	2	Q.rob-fr	Flora Incognita	Quercus robur	Woody	Fruit	50	50	2	needs 2 diff pics(penalty)	Q.robur(95%)(2pics)				
898	3	Q.rob-fr	Flora Incognita	Quercus robur	Woody	Fruit	40	45		needs 2 diff pics(penalty)	Q.rubra	Q.robur(2pics)			
899	4	Q.rob-fr	Flora Incognita	Quercus robur	Woody	Fruit	80	85		needs 2 pics(penalty)	Q.petraea	Q.robur(2pics)			
900	5	Q.rob-fr	Flora Incognita	Quercus robur	Woody	Fruit	80	80		needs 2 pics(penalty)	Turkish lime (Q robur with 2 pics)	Silver lie			1
901	1	Q.rob-pl	Flora Incognita	Quercus robur	Woody	Plant	0	0	2		unknown				
902	2	Q.rob-pl	Flora Incognita	Quercus robur	Woody	Plant	0	0			unknown				
903	3	Q.rob-pl	Flora Incognita	Quercus robur	Woody	Plant	0	0			unknown				
904	4	Q.rob-pl	Flora Incognita	Quercus robur	Woody	Plant	0	0			unknown				
905	5	Q.rob-pl	Flora Incognita	Quercus robur	Woody	Plant	80	80			Q.garryana				
906	1	E.nigr-fl	Flora Incognita	Empetrum nigrum	Woody	Flower	90	90	1	needs 2 pics(penalty)	E.nigrum				
907	2	E.nigr-fl	Flora Incognita	Empetrum nigrum	Woody	Flower	90	90		needs 2 pics(penalty)	Empetrum				
908	3	E.nigr-fl	Flora Incognita	Empetrum nigrum	Woody	Flower	100	100			Crowberry(99%)				
909	4	E.nigr-fl	Flora Incognita	Empetrum nigrum	Woody	Flower	100	100			Crowberry				
910	5	E.nigr-fl	Flora Incognita	Empetrum nigrum	Woody	Flower	100	100			Crowberry				
911	1	E.nigr-fr	Flora Incognita	Empetrum nigrum	Woody	Fruit	100	100	1		E.nigrum(99%)				
912	2	E.nigr-fr	Flora Incognita	Empetrum nigrum	Woody	Fruit	100	100			E.nigrum(92%)				
913	3	E.nigr-fr	Flora Incognita	Empetrum nigrum	Woody	Fruit	90	90		needs 2 pics	E.nigrum(92%)				
914	4	E.nigr-fr	Flora Incognita	Empetrum nigrum	Woody	Fruit	90	90		needs 2 pics	E.nigrum(92%)				
915	5	E.nigr-fr	Flora Incognita	Empetrum nigrum	Woody	Fruit	100	100			E.nigrum				
916	1	A.pseudo	Flora Incognita	Acer pseudoplatanus	Woody	Leaf	80	90	1.2		A.opalus	A.pseuodoplatanus			
917	2	A.pseudo	Flora Incognita	Acer pseudoplatanus	Woody	Leaf	100	100			A.pseudoplatanus(92%)				
918	3	A.pseudo	Flora Incognita	Acer pseudoplatanus	Woody	Leaf	100	100			A.pseudoplatanus(90%)				
919	4	A.pseudo	Flora Incognita	Acer pseudoplatanus	Woody	Leaf	100	100			A.pseudoplatanus(99%)				
920	5	A.pseudo	Flora Incognita	Acer pseudoplatanus	Woody	Leaf	100	100			A.pseudoplatanus(				
921	1	C.pauci	Flora Incognita	Carex pauciflora	Monocot	Flower	0	0	2		unknown				
922	2	C.pauci	Flora Incognita	Carex pauciflora	Monocot	Flower	90	90		needs 2 pics	C.pauciflora				
923	3	C.pauci	Flora Incognita	Carex pauciflora	Monocot	Flower	100	100			C.pauciflora				
924	4	C.pauci	Flora Incognita	Carex pauciflora	Monocot	Flower	0	0			unknown				
925	5	C.pauci	Flora Incognita	Carex pauciflora	Monocot	Flower	0	0			unknown				
926	1	C.Fuscus	Flora Incognita	Cyperus fuscus	Monocot	Plant	80	85	1.2	needs 2 pics	Cyoerus longus	C.fuscus			
927	2	C.Fuscus	Flora Incognita	Cyperus fuscus	Monocot	Plant	100	100			C.fuscus				
928	3	C.Fuscus	Flora Incognita	Cyperus fuscus	Monocot	Plant	100	100			C.fuscus				
929	4	C.Fuscus	Flora Incognita	Cyperus fuscus	Monocot	Plant	100	100			C.fuscus				
930	5	C.Fuscus	Flora Incognita	Cyperus fuscus	Monocot	Plant	100	100			C.fuscus(92%)				
931	1	T.marit	Flora Incognita	Triglochin maritima	Monocot	Plant	100	100	1		T.maritimum(93%)				
932	2	T.marit	Flora Incognita	Triglochin maritima	Monocot	Plant	100	100			T.maritimum(93%)				
933	3	T.marit	Flora Incognita	Triglochin maritima	Monocot	Plant	100	100			T.maritimum(90%)				
934	4	T.marit	Flora Incognita	Triglochin maritima	Monocot	Plant	100	100			T.maritimum(83%)				
935	5	T.marit	Flora Incognita	Triglochin maritima	Monocot	Plant	100	100			T.maritimum(88%)				
936	1	Delairia	Flora Incognita	Delairea odorata	Herb	Plant	100	100	3		unknown				
937	2	Delairia	Flora Incognita	Delairea odorata	Herb	Plant	100	100			Cape ivy				
938	3	Delairia	Flora Incognita	Delairea odorata	Herb	Plant	100	100			Cape ivy				
939	4	Delairia	Flora Incognita	Delairea odorata	Herb	Plant	100	100			Cecropia				1
940	5	Delairia	Flora Incognita	Delairea odorata	Herb	Plant	100	100			Unknown				
941	1	E.bonar	Flora Incognita	Erigeron bonariensis	Herb	Flower	100	100	1		E.bonariensis(95%)				
942	2	E.bonar	Flora Incognita	Erigeron bonariensis	Herb	Flower	100	100			E.bonariensis(90%)				
943	3	E.bonar	Flora Incognita	Erigeron bonariensis	Herb	Flower	100	100			E.bonariensis(99%)				
944	4	E.bonar	Flora Incognita	Erigeron bonariensis	Herb	Flower	100	100			E.bonariensis(88%)				
945	5	E.bonar	Flora Incognita	Erigeron bonariensis	Herb	Flower	100	100			E.bonariensis				
946	1	M.mosch	Flora Incognita	Malva moschata	Herb	Flower	80	90	1.2		M.alcea	M.moschata			
947	2	M.mosch	Flora Incognita	Malva moschata	Herb	Flower	70	80		needs 2 pics(10 penalty)	M.alcea	M.moschata			
948	3	M.mosch	Flora Incognita	Malva moschata	Herb	Flower	80	90			M.alcea	M.moschata			
949	4	M.mosch	Flora Incognita	Malva moschata	Herb	Flower	100	100			M.moschata				
950	5	M.mosch	Flora Incognita	Malva moschata	Herb	Flower	100	100			M.moschata				
951	1	P.lutea	Candide	Psuedofumaria lutea	Herb	Plant	0	0	4		Oxalis	Oxalis	Salvia		1
952	2	P.lutea	Candide	Psuedofumaria lutea	Herb	Plant	0	55			Aquilegia	Corydalis	Pale corydalis(+Pseudo fumaria)		1
953	3	P.lutea	Candide	Psuedofumaria lutea	Herb	Plant	80	80			Corydalis				
954	4	P.lutea	Candide	Psuedofumaria lutea	Herb	Plant	0	40			Tamarid	Corydalis			1
955	5	P.lutea	Candide	Psuedofumaria lutea	Herb	Plant	80	80			Corydalis	Tamarind			
956	1	D.purp.	Candide	Digitalis purpurea	Herb	Leaf	0	10	4		Adenostoma	Brunnera	Primula	Digitalis	1
957	2	D.purp.	Candide	Digitalis purpurea	Herb	Leaf	0	0			Primrose	Primula			1
958	3	D.purp.	Candide	Digitalis purpurea	Herb	Leaf	80	83			Digitalis	Adenostoma	Foxglove		
959	4	D.purp.	Candide	Digitalis purpurea	Herb	Leaf	0	0			Salvia	Adenostoma			1
960	5	D.purp.	Candide	Digitalis purpurea	Herb	Leaf	0	0			Primrose	Primula			1
961	1	S.coron.	Candide	Silene coronaria	Herb	Leaf	-5	-5	3		Oldenbergia				1
962	2	S.coron.	Candide	Silene coronaria	Herb	Leaf	10	10			Stachys	Mullein	Agave		1
963	3	S.coron.	Candide	Silene coronaria	Herb	Leaf	10	10			Mullein	Verbascum			1
964	4	S.coron.	Candide	Silene coronaria	Herb	Leaf	10	10			Verbascum				1
965	5	S.coron.	Candide	Silene coronaria	Herb	Leaf	10	10			Mullein	Verbascum			1
966	1	C.off.	Candide	Calendula officinalis	Herb	Plant	0	25	2.4		Cucurbita	Asteraceae			1
967	2	C.off.	Candide	Calendula officinalis	Herb	Plant	90	95			Calendula	Pot marigold			
968	3	C.off.	Candide	Calendula officinalis	Herb	Plant	50	50			Taraxacum	Picris			1
969	4	C.off.	Candide	Calendula officinalis	Herb	Plant	90	90			Calendula				
970	5	C.off.	Candide	Calendula officinalis	Herb	Plant	50	70			Coreopsis	Calendula			1
971	1	M.erecta	Candide	Moenchia erecta	Herb	Flower	-5	-5	2.4		Babiana	Xerophyta			1
972	2	M.erecta	Candide	Moenchia erecta	Herb	Flower	50	50			Sagina				1
973	3	M.erecta	Candide	Moenchia erecta	Herb	Flower	50	50			Stellaria				1
974	4	M.erecta	Candide	Moenchia erecta	Herb	Flower	50	50			Stellaria				1
975	5	M.erecta	Candide	Moenchia erecta	Herb	Flower	50	50			Stellaria media				1
976	1	S.verna	Candide	Sabulina verna	Herb	Plant	0	25	4		Xerophyta	Spergulara			1
977	2	S.verna	Candide	Sabulina verna	Herb	Plant	0	13			Parnassia	Heliophila	Spergularia		1
978	3	S.verna	Candide	Sabulina verna	Herb	Plant	50	50			Sagina				1
979	4	S.verna	Candide	Sabulina verna	Herb	Plant	50	50			Sagina				1
980	5	S.verna	Candide	Sabulina verna	Herb	Plant	0	25			Heliophila	Spergularia			1
981	1	E.marit.	Candide	Erodium maritimum	Herb	Leaf	0	0	4		Balota	Glechoma			1
982	2	E.marit.	Candide	Erodium maritimum	Herb	Leaf	80	80			Erodium spp.				
983	3	E.marit.	Candide	Erodium maritimum	Herb	Leaf	0	40			Lamium	Erodium spp.			1
984	4	E.marit.	Candide	Erodium maritimum	Herb	Leaf	-5	-5			Azolla	Nephrolepis			
985	5	E.marit.	Candide	Erodium maritimum	Herb	Leaf	80	80			Erodium spp.				
986	1	G.purp.	Candide	Geranium purpureum	Herb	Plant	90	90	2.2		Herb robert				
987	2	G.purp.	Candide	Geranium purpureum	Herb	Plant	80	85			Geranium	Herb robert			
988	3	G.purp.	Candide	Geranium purpureum	Herb	Plant	-5	45			Sundew	Erodium spp.	Geranium		1
989	4	G.purp.	Candide	Geranium purpureum	Herb	Plant	90	90			Herb robert				
990	5	G.purp.	Candide	Geranium purpureum	Herb	Plant	80	89			Geranium	Herb robert	Little robin		
991	1	A.diandra	Candide	Anisantha diandra	Monocot	Flower	0	0	4		Galingale				1
992	2	A.diandra	Candide	Anisantha diandra	Monocot	Flower	50	50			Cynodon				1
993	3	A.diandra	Candide	Anisantha diandra	Monocot	Flower	50	50			Vulpia				1
994	4	A.diandra	Candide	Anisantha diandra	Monocot	Flower	0	25			Tillandsia	Vupia			1
995	5	A.diandra	Candide	Anisantha diandra	Monocot	Flower	50	50			Vulpia				1
996	1	S.arv.-lf	Candide	Spergula arvensis	Herb	Leaf	90	90	5	Small genus	Spergula				
997	2	S.arv.-lf	Candide	Spergula arvensis	Herb	Leaf	0	23			Ceratophyllum	Cynodon	Spergula		1
998	3	S.arv.-lf	Candide	Spergula arvensis	Herb	Leaf	-5	-5			Sanseveria	Lebedouria			1
999	4	S.arv.-lf	Candide	Spergula arvensis	Herb	Leaf	0	45			Oxalis	Spergula			1
1000	5	S.arv.-lf	Candide	Spergula arvensis	Herb	Leaf	-5	-5			Pine tree	Cuminium	Ceratophyllum		1
1001	1	S.arv.-fl	Candide	Spergula arvensis	Herb	Flower	70	75	2.2		Spergularia	Sagina	Spergula		1
1002	2	S.arv.-fl	Candide	Spergula arvensis	Herb	Flower	90	90			Spergula				
1003	3	S.arv.-fl	Candide	Spergula arvensis	Herb	Flower	70	70			Spergularia				1
1004	4	S.arv.-fl	Candide	Spergula arvensis	Herb	Flower	0	0			Achillea	Sysirinchium	Olearia		1
1005	5	S.arv.-fl	Candide	Spergula arvensis	Herb	Flower	90	90			Spergula				
1006	1	S.arv.-pl	Candide	Spergula arvensis	Herb	Plant	0	25	2		Sibbaldiopsis	Cerastium			1
1007	2	S.arv.-pl	Candide	Spergula arvensis	Herb	Plant	0	25			Sibbaldiopsis	Gypsophila			1
1008	3	S.arv.-pl	Candide	Spergula arvensis	Herb	Plant	0	25			Sibbaldiopsis	Stellaria			1

1009	4	S.arv.-pl	Candide	Spergula arvensis	Herb	Plant	50	50			Silene				1
1010	5	S.arv.-pl	Candide	Spergula arvensis	Herb	Plant	0	13			Sibbaldiopsis	Raoulia	Gypsophila		1
1011	1	S.retic.	Candide	Salix reticulata	Woody	Leaf	100	100	3.2		Net-leaved willow				
1012	2	S.retic.	Candide	Salix reticulata	Woody	Leaf	80	90			Willow	Net-leaved willow			
1013	3	S.retic.	Candide	Salix reticulata	Woody	Leaf	-5	55			Lantern flower	Willow	Net-leaved willow		1
1014	4	S.retic.	Candide	Salix reticulata	Woody	Leaf	-5	55			Pilea	Willow	Net-leaved willow		1
1015	5	S.retic.	Candide	Salix reticulata	Woody	Leaf	80	90			Willow	Net-leaved willow			
1016	1	A.sylv-fl	Candide	Angelica sylvestris	Herb	Flower	50	50	1		Oenanthe				1
1017	2	A.sylv-fl	Candide	Angelica sylvestris	Herb	Flower	50	50			Oenanthe				1
1018	3	A.sylv-fl	Candide	Angelica sylvestris	Herb	Flower	50	50			Oenanthe				1
1019	4	A.sylv-fl	Candide	Angelica sylvestris	Herb	Flower	50	50			Oenanthe				1
1020	5	A.sylv-fl	Candide	Angelica sylvestris	Herb	Flower	50	50			Oenanthe				1
1021	1	A.sylv-lf	Candide	Angelica sylvestris	Herb	Leaf	-5	-5	3		Blechnum	Pellion			1
1022	2	A.sylv-lf	Candide	Angelica sylvestris	Herb	Leaf	-5	-5			Roses				1
1023	3	A.sylv-lf	Candide	Angelica sylvestris	Herb	Leaf	5	5			Ash	Roses			1
1024	4	A.sylv-lf	Candide	Angelica sylvestris	Herb	Leaf	-5	-5			Roses				1
1025	5	A.sylv-lf	Candide	Angelica sylvestris	Herb	Leaf	-5	-5			Roses				1
1026	1	A.syl-pl	Candide	Angelica sylvestris	Herb	Plant	-5	-5	2.6		Phragmites				1
1027	2	A.syl-pl	Candide	Angelica sylvestris	Herb	Plant	50	50			Foeniculum				1
1028	3	A.syl-pl	Candide	Angelica sylvestris	Herb	Plant	50	50			Hogweeds				1
1029	4	A.syl-pl	Candide	Angelica sylvestris	Herb	Plant	-5	-5			Echinops	Dandelion			1
1030	5	A.syl-pl	Candide	Angelica sylvestris	Herb	Plant	50	50			Daucus				1
1031	1	A.sylv-fr	Candide	Angelica sylvestris	Herb	Fruit	-5	-5	5		Teasels				1
1032	2	A.sylv-fr	Candide	Angelica sylvestris	Herb	Fruit	-5	-5			Xysmalobium	Pineapple			1
1033	3	A.sylv-fr	Candide	Angelica sylvestris	Herb	Fruit	-5	-5			Roseling	Turtle vine			1
1034	4	A.sylv-fr	Candide	Angelica sylvestris	Herb	Fruit	-5	-5			Crassula				1
1035	5	A.sylv-fr	Candide	Angelica sylvestris	Herb	Fruit	-5	-5			Pineapple				1
1036	1	A.caucal.-fl	Candide	Anthriscus caucalis	Herb	Flower	0	40	4		Erodium spp.	Anthriscus			1
1037	2	A.caucal.-fl	Candide	Anthriscus caucalis	Herb	Flower	0	0			Silene	Galium	Myosotis		1
1038	3	A.caucal.-fl	Candide	Anthriscus caucalis	Herb	Flower	80	85			Anthriscus	Ornithopus	A.caucalis		
1039	4	A.caucal.-fl	Candide	Anthriscus caucalis	Herb	Flower	0	13			Valerianella	Vicia	Galium	A.caucalis	1
1040	5	A.caucal.-fl	Candide	Anthriscus caucalis	Herb	Flower	0	0			Valerianella	Ornithopus			1
1041	1	A.caucal.-pl	Candide	Anthriscus caucalis	Herb	Plant	0	25	3.2		Cheilanthes	Meum			1
1042	2	A.caucal.-pl	Candide	Anthriscus caucalis	Herb	Plant	0	0			Tripleurospermum	Arabis			1
1043	3	A.caucal.-pl	Candide	Anthriscus caucalis	Herb	Plant	0	13			Pritzelago	Sagina	Meum		1
1044	4	A.caucal.-pl	Candide	Anthriscus caucalis	Herb	Plant	0	7			Pritzelago	Arabis	Crambe	Meum	1
1045	5	A.caucal.-pl	Candide	Anthriscus caucalis	Herb	Plant	80	80			Anthriscus				
1046	1	A.caucal-lf	Candide	Anthriscus caucalis	Herb	Leaf	-5	-5	4		Rabbit-foot fern				1
1047	2	A.caucal-lf	Candide	Anthriscus caucalis	Herb	Leaf	-5	-5			Haworthia	Crassula			1
1048	3	A.caucal-lf	Candide	Anthriscus caucalis	Herb	Leaf	-5	38			Araucaria	Anthriscus			1
1049	4	A.caucal-lf	Candide	Anthriscus caucalis	Herb	Leaf	-5	-5			Haworthia	Fabiana			1
1050	5	A.caucal-lf	Candide	Anthriscus caucalis	Herb	Leaf	0	0			Senecio	Fish-tail fern			1
1051	1	H.elod-Fl	Candide	Hypericum elodes	Herb	Flower	0	0	4		Echballium				1
1052	2	H.elod-Fl	Candide	Hypericum elodes	Herb	Flower	-5	-5			Spiderwort	Ivy			1
1053	3	H.elod-Fl	Candide	Hypericum elodes	Herb	Flower	-5	-5			Spiderwort	Salvia			1
1054	4	H.elod-Fl	Candide	Hypericum elodes	Herb	Flower	0	0			Oxalis	Salvia			1
1055	5	H.elod-Fl	Candide	Hypericum elodes	Herb	Flower	0	0			Mirabilis	Barleria			1
1056	1	H.elod-lf	Candide	Hypericum elodes	Herb	Leaf	0	0	4		Orchids				1
1057	2	H.elod-lf	Candide	Hypericum elodes	Herb	Leaf	10	10			Stachys	Lamb's ear			1
1058	3	H.elod-lf	Candide	Hypericum elodes	Herb	Leaf	0	0			Salvia	Cactus			1
1059	4	H.elod-lf	Candide	Hypericum elodes	Herb	Leaf	0	0			Nepeta	Taraxacum			1
1060	5	H.elod-lf	Candide	Hypericum elodes	Herb	Leaf	10	10			Stachys				1
1061	1	H.elod-pl	Candide	Hypericum elodes	Herb	Plant	0	0	5		unknown				
1062	2	H.elod-pl	Candide	Hypericum elodes	Herb	Plant	0	0			Senecio	String of tears			1
1063	3	H.elod-pl	Candide	Hypericum elodes	Herb	Plant	-5	-5			Loofah	Creeping fig			1
1064	4	H.elod-pl	Candide	Hypericum elodes	Herb	Plant	-5	-5			Crassula				1
1065	5	H.elod-pl	Candide	Hypericum elodes	Herb	Plant	-5	-5			Calisia	Crassula			1
1066	1	C.remot-fl1	Candide	Carex remota	Monocot	Flower	50	50	2.4	non-native genus	Schoenoxiphium				1
1067	2	C.remot-fl1	Candide	Carex remota	Monocot	Flower	10	10			Triraphis	Tristachya			1
1068	3	C.remot-fl1	Candide	Carex remota	Monocot	Flower	50	50		non-native genus	Schoenoxiphium				1
1069	4	C.remot-fl1	Candide	Carex remota	Monocot	Flower	50	50			Cyperaceae				
1070	5	C.remot-fl1	Candide	Carex remota	Monocot	Flower	50	50			Cyperus				1
1071	1	C.remot-pl	Candide	Carex remota	Monocot	Plant	10	10	5		Stentotaphrum				1
1072	2	C.remot-pl	Candide	Carex remota	Monocot	Plant	10	10			Juncus	Festuca			1
1073	3	C.remot-pl	Candide	Carex remota	Monocot	Plant	0	5			Plantago	Juncus	Pinus		1
1074	4	C.remot-pl	Candide	Carex remota	Monocot	Plant	0	5			Cardamine	Juncus			1
1075	5	C.remot-pl	Candide	Carex remota	Monocot	Plant	50	50		non-native genus	Schleria				1
1076	1	C.remot-fl2	Candide	Carex remota	Monocot	Flower	0	5	2.2		Plant	Eragrostis			
1077	2	C.remot-fl2	Candide	Carex remota	Monocot	Flower	10	10			Eragrostis	Spartina			1
1078	3	C.remot-fl2	Candide	Carex remota	Monocot	Flower	10	10			Eragrostis	Molinia			1
1079	4	C.remot-fl2	Candide	Carex remota	Monocot	Flower	10	20			Molinia	Wheat	Cyperaceae		1
1080	5	C.remot-fl2	Candide	Carex remota	Monocot	Flower	10	10			Molinia				1
1081	1	Q.rob-lf	Candide	Quercus robur	Woody	Leaf	100	100	5		Q.robur				
1082	2	Q.rob-lf	Candide	Quercus robur	Woody	Leaf	-5	-5			Asplenium	Plantago			1
1083	3	Q.rob-lf	Candide	Quercus robur	Woody	Leaf	-5	-5			Prayer plant	Fern			1
1084	4	Q.rob-lf	Candide	Quercus robur	Woody	Leaf	0	0			none				
1085	5	Q.rob-lf	Candide	Quercus robur	Woody	Leaf	-5	-5			Brassica	Brassica			1
1086	1	Q.rob-fr	Candide	Quercus robur	Woody	Fruit	-5	-5	5		Loofah	Marrow			1
1087	2	Q.rob-fr	Candide	Quercus robur	Woody	Fruit	-5	-5			Silene	Antizoma			1
1088	3	Q.rob-fr	Candide	Quercus robur	Woody	Fruit	-5	-5			Solanum	Landolfia			1
1089	4	Q.rob-fr	Candide	Quercus robur	Woody	Fruit	-5	-5			Rhadamanthus	Cycad			1
1090	5	Q.rob-fr	Candide	Quercus robur	Woody	Fruit	-5	-5			Araucaria	Solanum			1
1091	1	Q.rob-pl	Candide	Quercus robur	Woody	Plant	-5	-5	4		Pinus	Acacia			1
1092	2	Q.rob-pl	Candide	Quercus robur	Woody	Plant	-5	-5			Pinus	Hornbeam			1
1093	3	Q.rob-pl	Candide	Quercus robur	Woody	Plant	0	0			Wych elm	Ceratonia			1
1094	4	Q.rob-pl	Candide	Quercus robur	Woody	Plant	-5	-5			Fig	Hawthorn			1
1095	5	Q.rob-pl	Candide	Quercus robur	Woody	Plant	-5	-5			Salvia	Clubrush			1
1096	1	E.nigr-fl	Candide	Empetrum nigrum	Woody	Flower	0	0	5		Cocornia				1
1097	2	E.nigr-fl	Candide	Empetrum nigrum	Woody	Flower	-5	-5			Salicornia	Hatiora			1
1098	3	E.nigr-fl	Candide	Empetrum nigrum	Woody	Flower	-5	-5			Hatiora	Andromiscus			1
1099	4	E.nigr-fl	Candide	Empetrum nigrum	Woody	Flower	-5	-5			Alternanthera				1
1100	5	E.nigr-fl	Candide	Empetrum nigrum	Woody	Flower	-5	-5			Echeverria				1
1101	1	E.nigr-fr	Candide	Empetrum nigrum	Woody	Fruit	10	10	3		Juniper				1
1102	2	E.nigr-fr	Candide	Empetrum nigrum	Woody	Fruit	0	0			no match				
1103	3	E.nigr-fr	Candide	Empetrum nigrum	Woody	Fruit	10	30			Juniper	Gaultheria			1
1104	4	E.nigr-fr	Candide	Empetrum nigrum	Woody	Fruit	50	50			Vaccinium				
1105	5	E.nigr-fr	Candide	Empetrum nigrum	Woody	Fruit	-5	-5			Araucaria				1
1106	1	A.pseudo	Candide	Acer pseudoplatanus	Woody	Leaf	80	90	4		Maples	Sycamore			
1107	2	A.pseudo	Candide	Acer pseudoplatanus	Woody	Leaf	70	70			Planes	Ribes			
1108	3	A.pseudo	Candide	Acer pseudoplatanus	Woody	Leaf	80	90			Maples	Sycamore			
1109	4	A.pseudo	Candide	Acer pseudoplatanus	Woody	Leaf	20	20		similar	Ribes				1
1110	5	A.pseudo	Candide	Acer pseudoplatanus	Woody	Leaf	0	0			Oplopanax				1
1111	1	C.pauci	Candide	Carex pauciflora	Monocot	Flower	10	25	2.4	Non-native genus	Cynodon	Pycreus			1
1112	2	C.pauci	Candide	Carex pauciflora	Monocot	Flower	10	30			Cynodon	Cyperus			1
1113	3	C.pauci	Candide	Carex pauciflora	Monocot	Flower	10	10			Stentotaphrum	Grama			1
1114	4	C.pauci	Candide	Carex pauciflora	Monocot	Flower	10	10			Cynodon	Oats			1
1115	5	C.pauci	Candide	Carex pauciflora	Monocot	Flower	50	50			Galingale				1
1116	1	C.Fuscus	Candide	Cyperus fuscus	Monocot	Plant	80	80	1.2		Cyperus	C.eragostis			
1117	2	C.Fuscus	Candide	Cyperus fuscus	Monocot	Plant	80	80			Cyperus	Juncus			
1118	3	C.Fuscus	Candide	Cyperus fuscus	Monocot	Plant	50	50			Scirpoides	Juncus			1
1119	4	C.Fuscus	Candide	Cyperus fuscus	Monocot	Plant	80	80			Cyperus	Juncus			
1120	5	C.Fuscus	Candide	Cyperus fuscus	Monocot	Plant	80	80			Cyperus	Juncus			
1121	1	T.marit	Candide	Triglochin maritima	Monocot	Plant	90	90	2		Triglochin				
1122	2	T.marit	Candide	Triglochin maritima	Monocot	Plant	90	90			Triglochin				
1123	3	T.marit	Candide	Triglochin maritima	Monocot	Plant	10	10		Looks similarish	Juncus	Scirpoides			1
1124	4	T.marit	Candide	Triglochin maritima	Monocot	Plant	90	90			Triglochin				
1125	5	T.marit	Candide	Triglochin maritima	Monocot	Plant	90	90			Triglochin				
1126	1	Delairia	Candide	Delairea odorata	Herb	Plant	5	5	4	similar to vitis	Achillea	Vitis			1
1127	2	Delairia	Candide	Delairea odorata	Herb	Plant	-5	-5			Cylindrophyllum	Macaranga			1
1128	3	Delairia	Candide	Delairea odorata	Herb	Plant	-5	-5			Lamium	Echballium			1
1129	4	Delairia	Candide	Delairea odorata	Herb	Plant	0	0			Cissus				1
1130	5	Delairia	Candide	Delairea odorata	Herb	Plant	0	0			Cissus				1

1131	1	E.bonar	Candide	Erigeron bonariensis	Herb	Flower	50	80	3.2		Fleabane	Sonchus	E.acer		
1132	2	E.bonar	Candide	Erigeron bonariensis	Herb	Flower	-5	-5			Dryopteris	Daucus			1
1133	3	E.bonar	Candide	Erigeron bonariensis	Herb	Flower	0	0			Lagurus	Daucus			1
1134	4	E.bonar	Candide	Erigeron bonariensis	Herb	Flower	80	80			Erigeron				
1135	5	E.bonar	Candide	Erigeron bonariensis	Herb	Flower	0	50			Achillea	Silene			1
1136	1	M.mosch	Candide	Malva moschata	Herb	Flower	80	80	2.2		Malva	Sidalcea			
1137	2	M.mosch	Candide	Malva moschata	Herb	Flower	80	80			Malva	Sidalcea			
1138	3	M.mosch	Candide	Malva moschata	Herb	Flower	0	40			Cosmos	Malva			1
1139	4	M.mosch	Candide	Malva moschata	Herb	Flower	80	80			Malva				
1140	5	M.mosch	Candide	Malva moschata	Herb	Flower	50	58			Alogyne	Geranium incanum	Malva		1
1141	1	P.lutea	Bing	Psuedofumaria lutea	Herb	Plant	90	90	2		Pseudofumaria				
1142	2	P.lutea	Bing	Psuedofumaria lutea	Herb	Plant	90	90			Pseudofumaria				
1143	3	P.lutea	Bing	Psuedofumaria lutea	Herb	Plant	90	90			Pseudofumaria				
1144	4	P.lutea	Bing	Psuedofumaria lutea	Herb	Plant	90	90			Pseudofumaria				
1145	5	P.lutea	Bing	Psuedofumaria lutea	Herb	Plant	-5	-5			Pointsettia	Perennial flower			1
1146	1	D.purp.	Bing	Digitalis purpurea	Herb	Leaf	-5	-5	3		"Leaves"				
1147	2	D.purp.	Bing	Digitalis purpurea	Herb	Leaf	-5	-5			"Leaves"				
1148	3	D.purp.	Bing	Digitalis purpurea	Herb	Leaf	0	0			Lamium	"Leaves"			1
1149	4	D.purp.	Bing	Digitalis purpurea	Herb	Leaf	-5	-5			"Leaves"				
1150	5	D.purp.	Bing	Digitalis purpurea	Herb	Leaf	-5	-5			Rheum				1
1151	1	S.coron.	Bing	Silene coronaria	Herb	Leaf	-5	-5	3		Agave				1
1152	2	S.coron.	Bing	Silene coronaria	Herb	Leaf	10	10			Verbasum	Agave			1
1153	3	S.coron.	Bing	Silene coronaria	Herb	Leaf	10	10			Verbascum	Agave			1
1154	4	S.coron.	Bing	Silene coronaria	Herb	Leaf	10	10			Verbascum	Perennial			1
1155	5	S.coron.	Bing	Silene coronaria	Herb	Leaf	-5	-5			Irrelevant				1
1156	1	C.off.	Bing	Calendula officinalis	Herb	Plant	-5	18	3		"Autumn"	Helianthus			1
1157	2	C.off.	Bing	Calendula officinalis	Herb	Plant	-5	7			"Leaves"	Jackfruit	Sunflower		
1158	3	C.off.	Bing	Calendula officinalis	Herb	Plant	0	0			Iris	Lily			1
1159	4	C.off.	Bing	Calendula officinalis	Herb	Plant	-5	-5			"Leaves"	Aloe	Cactus		
1160	5	C.off.	Bing	Calendula officinalis	Herb	Plant	-5	-5			"Leaves"	Lily			
1161	1	M.erecta	Bing	Moenchia erecta	Herb	Flower	-5	-5	3		Bellflower				1
1162	2	M.erecta	Bing	Moenchia erecta	Herb	Flower	50	50			Cerastium				1
1163	3	M.erecta	Bing	Moenchia erecta	Herb	Flower	-5	-5			Wildflower	Ornithogallum			
1164	4	M.erecta	Bing	Moenchia erecta	Herb	Flower	-5	25			Wildflower	Stellaria			
1165	5	M.erecta	Bing	Moenchia erecta	Herb	Flower	-5	-5			Wildflower	Ligustrum			
1166	1	S.verna	Bing	Sabulina verna	Herb	Plant	-5	-5	5	each irrelev=1	Irrelevant				1
1167	2	S.verna	Bing	Sabulina verna	Herb	Plant	-5	-5			Irrelevant				1
1168	3	S.verna	Bing	Sabulina verna	Herb	Plant	-5	-5			Grass	Irrelevant			1
1169	4	S.verna	Bing	Sabulina verna	Herb	Plant	-5	-5			Irrelevant				1
1170	5	S.verna	Bing	Sabulina verna	Herb	Plant	-5	7			Irrelevant	flower website	flower website	Caryophyllaceae	1
1171	1	E.marit.	Bing	Erodium maritimum	Herb	Leaf	0	0	5		Marchantia				1
1172	2	E.marit.	Bing	Erodium maritimum	Herb	Leaf	-5	-5			Irrelevant				1
1173	3	E.marit.	Bing	Erodium maritimum	Herb	Leaf	0	0			Leptinella	Zucchini			1
1174	4	E.marit.	Bing	Erodium maritimum	Herb	Leaf	0	0			Liverworts	irrelevant			1
1175	5	E.marit.	Bing	Erodium maritimum	Herb	Leaf	-5	-5			Ground cover				1
1176	1	G.purp.	Bing	Geranium purpureum	Herb	Plant	50	50	5		Erodium				1
1177	2	G.purp.	Bing	Geranium purpureum	Herb	Plant	-5	-5			Croton				1
1178	3	G.purp.	Bing	Geranium purpureum	Herb	Plant	-5	-5			Aloe	Willow			1
1179	4	G.purp.	Bing	Geranium purpureum	Herb	Plant	-5	-5			Persicaria	irrelevant			1
1180	5	G.purp.	Bing	Geranium purpureum	Herb	Plant	-5	-5			irrelevant				1
1181	1	A.diandra	Bing	Anisantha diandra	Monocot	Flower	-5	-5	4		irrelevant	Bromeliads			1
1182	2	A.diandra	Bing	Anisantha diandra	Monocot	Flower	-5	-5			irrelevant				1
1183	3	A.diandra	Bing	Anisantha diandra	Monocot	Flower	50	50			Grass	Ficus			
1184	4	A.diandra	Bing	Anisantha diandra	Monocot	Flower	0	25			Grass seed	Grass			
1185	5	A.diandra	Bing	Anisantha diandra	Monocot	Flower	50	50			Grass	Tree			
1186	1	S.arv.-lf	Bing	Spergula arvensis	Herb	Leaf	-5	-5	2		Houstonia	Actaea			1
1187	2	S.arv.-lf	Bing	Spergula arvensis	Herb	Leaf	90	90			Spergula				
1188	3	S.arv.-lf	Bing	Spergula arvensis	Herb	Leaf	90	90			Spergula				
1189	4	S.arv.-lf	Bing	Spergula arvensis	Herb	Leaf	90	90			Spergula				
1190	5	S.arv.-lf	Bing	Spergula arvensis	Herb	Leaf	90	90			Spergula				
1191	1	S.arv.-fl	Bing	Spergula arvensis	Herb	Flower	90	90	1		Spergula				
1192	2	S.arv.-fl	Bing	Spergula arvensis	Herb	Flower	90	90			Spergula				
1193	3	S.arv.-fl	Bing	Spergula arvensis	Herb	Flower	90	90			Spergula				
1194	4	S.arv.-fl	Bing	Spergula arvensis	Herb	Flower	90	90			Spergula				
1195	5	S.arv.-fl	Bing	Spergula arvensis	Herb	Flower	90	90			Spergula				
1196	1	S.arv.-pl	Bing	Spergula arvensis	Herb	Plant	-5	-5	4		Perennial flowers	Houseplant			
1197	2	S.arv.-pl	Bing	Spergula arvensis	Herb	Plant	-5	-5			Jasmine	Gardenia			1
1198	3	S.arv.-pl	Bing	Spergula arvensis	Herb	Plant	-5	-1			Jasmine	Jasmine	Saxifraga		1
1199	4	S.arv.-pl	Bing	Spergula arvensis	Herb	Plant	-5	-5			Native plants	Gardenia			
1200	5	S.arv.-pl	Bing	Spergula arvensis	Herb	Plant	-5	-5			Saxifraga	Phlox			1
1201	1	S.retic.	Bing	Salix reticulata	Woody	Leaf	-5	-5	5		irrelevant				1
1202	2	S.retic.	Bing	Salix reticulata	Woody	Leaf	-5	-5			Sage	Salvia			1
1203	3	S.retic.	Bing	Salix reticulata	Woody	Leaf	-5	-5			Plectranthus	Seeds	Cecropia		1
1204	4	S.retic.	Bing	Salix reticulata	Woody	Leaf	-5	-5			Indoor plants	Sage	bacteria		1
1205	5	S.retic.	Bing	Salix reticulata	Woody	Leaf	-5	-5			Plant varieties	Indoor plants	Cecropia		
1206	1	A.sylv-fl	Bing	Angelica sylvestris	Herb	Flower	0	10	3		Milkweed	Allium	Lavender	Angelica	1
1207	2	A.sylv-fl	Bing	Angelica sylvestris	Herb	Flower	-5	-5			Lavender	Milkweed			1
1208	3	A.sylv-fl	Bing	Angelica sylvestris	Herb	Flower	0	0		slightly similar	Allium	Perennial flower			1
1209	4	A.sylv-fl	Bing	Angelica sylvestris	Herb	Flower	0	25		slightly similar	Allium	Sanicle			1
1210	5	A.sylv-fl	Bing	Angelica sylvestris	Herb	Flower	0	0		slightly similar	Allium	Allium	Allium		1
1211	1	A.sylv-lf	Bing	Angelica sylvestris	Herb	Leaf	-5	-5	5		Climbing vine				1
1212	2	A.sylv-lf	Bing	Angelica sylvestris	Herb	Leaf	-5	-5			Cirsium	Piper			1
1213	3	A.sylv-lf	Bing	Angelica sylvestris	Herb	Leaf	-5	-5			Lawn weeds	Grass			1
1214	4	A.sylv-lf	Bing	Angelica sylvestris	Herb	Leaf	-5	-5			irrelevant				1
1215	5	A.sylv-lf	Bing	Angelica sylvestris	Herb	Leaf	-5	-5			Ficus	irrelevant			1
1216	1	A.syl-pl	Bing	Angelica sylvestris	Herb	Plant	80	80	3		Angelica				
1217	2	A.syl-pl	Bing	Angelica sylvestris	Herb	Plant	80	80			Angelica				
1218	3	A.syl-pl	Bing	Angelica sylvestris	Herb	Plant	-5	-5			Bamboo	Grass			1
1219	4	A.syl-pl	Bing	Angelica sylvestris	Herb	Plant	80	80			Angelica				
1220	5	A.syl-pl	Bing	Angelica sylvestris	Herb	Plant	-5	-5			Grass				1
1221	1	A.sylv-fr	Bing	Angelica sylvestris	Herb	Fruit	-5	-5	4	fruits not similar	Allium				1
1222	2	A.sylv-fr	Bing	Angelica sylvestris	Herb	Fruit	-5	-5			Succulents	irrelevant			1
1223	3	A.sylv-fr	Bing	Angelica sylvestris	Herb	Fruit	-5	-5			Petasites	Succulents			1
1224	4	A.sylv-fr	Bing	Angelica sylvestris	Herb	Fruit	-5	-5			Stick insect	Aeonium			1
1225	5	A.sylv-fr	Bing	Angelica sylvestris	Herb	Fruit	-5	-5			Allium	irrelevant			1
1226	1	A.caucal.-fl	Bing	Anthriscus caucalis	Herb	Flower	70	70	3.4		Torilis				
1227	2	A.caucal.-fl	Bing	Anthriscus caucalis	Herb	Flower	0	25			Cornsalad	Mountain Sweet cicely			1
1228	3	A.caucal.-fl	Bing	Anthriscus caucalis	Herb	Flower	50	50			Mountain Sweet cicely				1
1229	4	A.caucal.-fl	Bing	Anthriscus caucalis	Herb	Flower	85	85			Caucalis platycarpos				
1230	5	A.caucal.-fl	Bing	Anthriscus caucalis	Herb	Flower	0	25			Cryptantha	Torilis			1
1231	1	A.caucal.-pl	Bing	Anthriscus caucalis	Herb	Plant	80	80	4		Anthriscus				
1232	2	A.caucal.-pl	Bing	Anthriscus caucalis	Herb	Plant	-5	-5			Eriophorum	Leptospermum			1
1233	3	A.caucal.-pl	Bing	Anthriscus caucalis	Herb	Plant	-5	-5			Flowers	Wildflower	Saxifrage		
1234	4	A.caucal.-pl	Bing	Anthriscus caucalis	Herb	Plant	-5	-5			Forget-me-not	Flowers			1
1235	5	A.caucal.-pl	Bing	Anthriscus caucalis	Herb	Plant	-5	-5			Eriophorum	irrelevant			1
1236	1	A.caucal-lf	Bing	Anthriscus caucalis	Herb	Leaf	50	50	3		Conium				1
1237	2	A.caucal-lf	Bing	Anthriscus caucalis	Herb	Leaf	-5	-5			Tomato	Cocnut			1
1238	3	A.caucal-lf	Bing	Anthriscus caucalis	Herb	Leaf	-5	-5			Succulent	Cactus			1
1239	4	A.caucal-lf	Bing	Anthriscus caucalis	Herb	Leaf	-5	-5			Succulent	Aloe			1
1240	5	A.caucal-lf	Bing	Anthriscus caucalis	Herb	Leaf	-5	-5			Tomato	Vegetable garden			1
1241	1	H.elod-Fl	Bing	Hypericum elodes	Herb	Flower	-5	-5	4		irrelevant				1
1242	2	H.elod-Fl	Bing	Hypericum elodes	Herb	Flower	-5	-5			Buddha	Tequila mexicana			1
1243	3	H.elod-Fl	Bing	Hypericum elodes	Herb	Flower	-5	-5			Tequila mexicana	Herbal remedies			1
1244	4	H.elod-Fl	Bing	Hypericum elodes	Herb	Flower	-5	-5			Moth	Knitted jumper			1
1245	5	H.elod-Fl	Bing	Hypericum elodes	Herb	Flower	-5	-5			Tequila mexicana	irrelevant			1
1246	1	H.elod-lf	Bing	Hypericum elodes	Herb	Leaf	10	10	4		Lamb's ear				1
1247	2	H.elod-lf	Bing	Hypericum elodes	Herb	Leaf	10	10			Lamb's ear				1
1248	3	H.elod-lf	Bing	Hypericum elodes	Herb	Leaf	-5	-5			Towel				1
1249	4	H.elod-lf	Bing	Hypericum elodes	Herb	Leaf	-5	-5			Clothes				1
1250	5	H.elod-lf	Bing	Hypericum elodes	Herb	Leaf	-5	-5			Knitting				1
1251	1	H.elod-pl	Bing	Hypericum elodes	Herb	Plant	-5	-5	5		Hebe	Hedera			1

1252	2	H.elod-pl	Bing	Hypericum elodes	Herb	Plant	-5	-5			irrelevant				1
1253	3	H.elod-pl	Bing	Hypericum elodes	Herb	Plant	10	10			Lamb's ear				1
1254	4	H.elod-pl	Bing	Hypericum elodes	Herb	Plant	0	0			Salix	irrelevant			1
1255	5	H.elod-pl	Bing	Hypericum elodes	Herb	Plant	-5	-5			irrelevant				1
1256	1	C.remot-fl1	Bing	Carex remota	Monocot	Flower	10	10	4		Grasses				1
1257	2	C.remot-fl1	Bing	Carex remota	Monocot	Flower	-5	-5			Stick insect				1
1258	3	C.remot-fl1	Bing	Carex remota	Monocot	Flower	-5	-5			Liatris	Baculum			1
1259	4	C.remot-fl1	Bing	Carex remota	Monocot	Flower	-5	-5			Liatris	irrelevant			1
1260	5	C.remot-fl1	Bing	Carex remota	Monocot	Flower	80	80			Carex				
1261	1	C.remot-pl	Bing	Carex remota	Monocot	Plant	-5	-5	5		irrelevant				1
1262	2	C.remot-pl	Bing	Carex remota	Monocot	Plant	-5	-5			Garlick	Ladybird			1
1263	3	C.remot-pl	Bing	Carex remota	Monocot	Plant	-5	-5			Giraffe				1
1264	4	C.remot-pl	Bing	Carex remota	Monocot	Plant	-5	-5			Succulent	Necklace			1
1265	5	C.remot-pl	Bing	Carex remota	Monocot	Plant	0	0			Golden rod				1
1266	1	C.remot-fl2	Bing	Carex remota	Monocot	Flower	10	10	1.2		Grass				1
1267	2	C.remot-fl2	Bing	Carex remota	Monocot	Flower	10	10			Grass				1
1268	3	C.remot-fl2	Bing	Carex remota	Monocot	Flower	10	10			Ornamental grass				1
1269	4	C.remot-fl2	Bing	Carex remota	Monocot	Flower	10	10			Grass				1
1270	5	C.remot-fl2	Bing	Carex remota	Monocot	Flower	10	10			Ornamental grass				1
1271	1	Q.rob-lf	Bing	Quercus robur	Woody	Leaf	80	80	5		Quercus				
1272	2	Q.rob-lf	Bing	Quercus robur	Woody	Leaf	20	20		ish	Acer	Agrimonia			1
1273	3	Q.rob-lf	Bing	Quercus robur	Woody	Leaf	-5	-5			Yoga				1
1274	4	Q.rob-lf	Bing	Quercus robur	Woody	Leaf	-5	-5			Orange	Leaves			1
1275	5	Q.rob-lf	Bing	Quercus robur	Woody	Leaf	-5	-5			irrelevant				1
1276	1	Q.rob-fr	Bing	Quercus robur	Woody	Fruit	0	0	4		Fruit tree	Walnut			1
1277	2	Q.rob-fr	Bing	Quercus robur	Woody	Fruit	-5	-5			Tomato	Fruit tree			1
1278	3	Q.rob-fr	Bing	Quercus robur	Woody	Fruit	80	80			Oak tree	Walnut tree			
1279	4	Q.rob-fr	Bing	Quercus robur	Woody	Fruit	0	0			Fruit tree	Guava			1
1280	5	Q.rob-fr	Bing	Quercus robur	Woody	Fruit	20	20			Chestnut	Fruit tree			1
1281	1	Q.rob-pl	Bing	Quercus robur	Woody	Plant	80	80	4		Quercus				
1282	2	Q.rob-pl	Bing	Quercus robur	Woody	Plant	0	40			Magnolia	Quercus			1
1283	3	Q.rob-pl	Bing	Quercus robur	Woody	Plant	40	40			Tree	Oak			
1284	4	Q.rob-pl	Bing	Quercus robur	Woody	Plant	-5	-5			Pine	Spruce			1
1285	5	Q.rob-pl	Bing	Quercus robur	Woody	Plant	0	20			Tree	Forest	Oak		
1286	1	E.nigr-fl	Bing	Empetrum nigrum	Woody	Flower	-5	-5	5		irrelevant				1
1287	2	E.nigr-fl	Bing	Empetrum nigrum	Woody	Flower	-5	-5			Prada	Rosemary	Butterfly		1
1288	3	E.nigr-fl	Bing	Empetrum nigrum	Woody	Flower	-5	-5			irrelevant				1
1289	4	E.nigr-fl	Bing	Empetrum nigrum	Woody	Flower	-5	-5			irrelevant				1
1290	5	E.nigr-fl	Bing	Empetrum nigrum	Woody	Flower	-5	-5			irrelevant				1
1291	1	E.nigr-fr	Bing	Empetrum nigrum	Woody	Fruit	80	80	3		Empetrum				
1292	2	E.nigr-fr	Bing	Empetrum nigrum	Woody	Fruit	50	50			Blueberry	Fruit trees			1
1293	3	E.nigr-fr	Bing	Empetrum nigrum	Woody	Fruit	10	45			Juniperus	Empetrum			1
1294	4	E.nigr-fr	Bing	Empetrum nigrum	Woody	Fruit	10	30			Juniperus	Blueberry			1
1295	5	E.nigr-fr	Bing	Empetrum nigrum	Woody	Fruit	50	50			Blueberry				1
1296	1	A.pseudo	Bing	Acer pseudoplatanus	Woody	Leaf	-5	-5	5		Leaves	Mint			
1297	2	A.pseudo	Bing	Acer pseudoplatanus	Woody	Leaf	-5	-5			irrelevant				1
1298	3	A.pseudo	Bing	Acer pseudoplatanus	Woody	Leaf	-5	-5			irrelevant				1
1299	4	A.pseudo	Bing	Acer pseudoplatanus	Woody	Leaf	-5	-5			Environmental workflow	irrelevant			1
1300	5	A.pseudo	Bing	Acer pseudoplatanus	Woody	Leaf	-5	-5			Umami	Environmental workflow			1
1301	1	C.pauci	Bing	Carex pauciflora	Monocot	Flower	-5	-5	5		Orchids	Cerastium			1
1302	2	C.pauci	Bing	Carex pauciflora	Monocot	Flower	-5	-5			Buds	irrelevant			1
1303	3	C.pauci	Bing	Carex pauciflora	Monocot	Flower	-5	-5			Wildflower guide	Insect			1
1304	4	C.pauci	Bing	Carex pauciflora	Monocot	Flower	-5	-5			Ophioglossum	Twig			1
1305	5	C.pauci	Bing	Carex pauciflora	Monocot	Flower	-5	-5			Cerastium	Koeleria			1
1306	1	C.Fuscus	Bing	Cyperus fuscus	Monocot	Plant	-5	-5	3		Spider				1
1307	2	C.Fuscus	Bing	Cyperus fuscus	Monocot	Plant	-5	-5			Spider				1
1308	3	C.Fuscus	Bing	Cyperus fuscus	Monocot	Plant	-5	-5			irrelevant				1
1309	4	C.Fuscus	Bing	Cyperus fuscus	Monocot	Plant	-5	-5			Spider	irrelevant	irrelevant	Cyperus	1
1310	5	C.Fuscus	Bing	Cyperus fuscus	Monocot	Plant	80	80			Cyperus				
1311	1	T.marit	Bing	Triglochin maritima	Monocot	Plant	0	0	2		Grass				1
1312	2	T.marit	Bing	Triglochin maritima	Monocot	Plant	0	0			Grass				1
1313	3	T.marit	Bing	Triglochin maritima	Monocot	Plant	0	0			Grass				1
1314	4	T.marit	Bing	Triglochin maritima	Monocot	Plant	90	90			Triglochin				
1315	5	T.marit	Bing	Triglochin maritima	Monocot	Plant	90	90			Triglochin				
1316	1	Delairia	Bing	Delairea odorata	Herb	Plant	100	100	1	monospecific genus	Delairea				
1317	2	Delairia	Bing	Delairea odorata	Herb	Plant	100	100		monospecific genus	Delairea				
1318	3	Delairia	Bing	Delairea odorata	Herb	Plant	100	100		monospecific genus	Delairea				
1319	4	Delairia	Bing	Delairea odorata	Herb	Plant	100	100		monospecific genus	Delairea				
1320	5	Delairia	Bing	Delairea odorata	Herb	Plant	100	100		monospecific genus	Delairea				
1321	1	E.bonar	Bing	Erigeron bonariensis	Herb	Flower	-5	-5	4.2		Ranunculus	Dames violet			1
1322	2	E.bonar	Bing	Erigeron bonariensis	Herb	Flower	-5	-5			Dames violet	malva			1
1323	3	E.bonar	Bing	Erigeron bonariensis	Herb	Flower	-5	-5			Malva	Cerastium			1
1324	4	E.bonar	Bing	Erigeron bonariensis	Herb	Flower	80	80			Erigeron				
1325	5	E.bonar	Bing	Erigeron bonariensis	Herb	Flower	80	80			Conyza				
1326	1	M.mosch	Bing	Malva moschata	Herb	Flower	-5	-5	5		Flickr	Erodium			1
1327	2	M.mosch	Bing	Malva moschata	Herb	Flower	-5	23			Purple flower	M.moschata			1
1328	3	M.mosch	Bing	Malva moschata	Herb	Flower	50	50			Sidalcia	Purple flower			1
1329	4	M.mosch	Bing	Malva moschata	Herb	Flower	-5	-5			Dierama	irrelevant			1
1330	5	M.mosch	Bing	Malva moschata	Herb	Flower	-5	-5			Birth flowers	Hibiscus			1
1331	1	P.lutea	iPlant (Plant Identif	Psuedofumaria lutea	Herb	Plant	-5	-5	5		Sensitive plant				1
1332	2	P.lutea	iPlant (Plant Identif	Psuedofumaria lutea	Herb	Plant	0	0			Ranunculus repens				1
1333	3	P.lutea	iPlant (Plant Identif	Psuedofumaria lutea	Herb	Plant	-5	-5			Redwood	Sorrel			1
1334	4	P.lutea	iPlant (Plant Identif	Psuedofumaria lutea	Herb	Plant	-5	-5			Nymphaea				1
1335	5	P.lutea	iPlant (Plant Identif	Psuedofumaria lutea	Herb	Plant	-5	-5			Wolfsbane				1
1336	1	D.purp.	iPlant (Plant Identif	Digitalis purpurea	Herb	Leaf	-5	-5	5		Stem				
1337	2	D.purp.	iPlant (Plant Identif	Digitalis purpurea	Herb	Leaf	-5	-5			Grapevine				1
1338	3	D.purp.	iPlant (Plant Identif	Digitalis purpurea	Herb	Leaf	-5	-5			Annual Plant				1
1339	4	D.purp.	iPlant (Plant Identif	Digitalis purpurea	Herb	Leaf	-5	-5			Flowers				1
1340	5	D.purp.	iPlant (Plant Identif	Digitalis purpurea	Herb	Leaf	0	0			Coltsfoot				1
1341	1	S.coron.	iPlant (Plant Identif	Silene coronaria	Herb	Leaf	-5	-5	5		Flowers				1
1342	2	S.coron.	iPlant (Plant Identif	Silene coronaria	Herb	Leaf	-5	-5			Agave				1
1343	3	S.coron.	iPlant (Plant Identif	Silene coronaria	Herb	Leaf	-5	-5			Stonecrops				1
1344	4	S.coron.	iPlant (Plant Identif	Silene coronaria	Herb	Leaf	-5	-5			Coca family				1
1345	5	S.coron.	iPlant (Plant Identif	Silene coronaria	Herb	Leaf	-5	-5			Houseplant				1
1346	1	C.off.	iPlant (Plant Identif	Calendula officinalis	Herb	Plant	50	50	1.6		Chrysanthemum				1
1347	2	C.off.	iPlant (Plant Identif	Calendula officinalis	Herb	Plant	90	90			Field marigold				
1348	3	C.off.	iPlant (Plant Identif	Calendula officinalis	Herb	Plant	90	90			Field marigold				
1349	4	C.off.	iPlant (Plant Identif	Calendula officinalis	Herb	Plant	100	100			Pot marigold				
1350	5	C.off.	iPlant (Plant Identif	Calendula officinalis	Herb	Plant	50	50			Tickseed				1
1351	1	M.erecta	iPlant (Plant Identif	Moenchia erecta	Herb	Flower	0	0	3		none				
1352	2	M.erecta	iPlant (Plant Identif	Moenchia erecta	Herb	Flower	0	0			Bud				
1353	3	M.erecta	iPlant (Plant Identif	Moenchia erecta	Herb	Flower	50	50			Chickweeds				
1354	4	M.erecta	iPlant (Plant Identif	Moenchia erecta	Herb	Flower	0	0			Bud				
1355	5	M.erecta	iPlant (Plant Identif	Moenchia erecta	Herb	Flower	50	50			Chickweeds				
1356	1	S.verna	iPlant (Plant Identif	Sabulina verna	Herb	Plant	0	0	2		Lilies				1
1357	2	S.verna	iPlant (Plant Identif	Sabulina verna	Herb	Plant	-5	-5			Crocus				1
1358	3	S.verna	iPlant (Plant Identif	Sabulina verna	Herb	Plant	0	0			Lilies				1
1359	4	S.verna	iPlant (Plant Identif	Sabulina verna	Herb	Plant	0	0			Liles				1
1360	5	S.verna	iPlant (Plant Identif	Sabulina verna	Herb	Plant	-5	-5			Crocus				1
1361	1	E.marit.	iPlant (Plant Identif	Erodium maritimum	Herb	Leaf	0	0	3		Liverworts				1
1362	2	E.marit.	iPlant (Plant Identif	Erodium maritimum	Herb	Leaf	0	0			Liverworts				1
1363	3	E.marit.	iPlant (Plant Identif	Erodium maritimum	Herb	Leaf	0	0			Flowers				
1364	4	E.marit.	iPlant (Plant Identif	Erodium maritimum	Herb	Leaf	-5	-5			Fern				1
1365	5	E.marit.	iPlant (Plant Identif	Erodium maritimum	Herb	Leaf	-5	-5			Fern				1
1366	1	G.purp.	iPlant (Plant Identif	Geranium purpureum	Herb	Plant	50	50	3		Geraniaceae				
1367	2	G.purp.	iPlant (Plant Identif	Geranium purpureum	Herb	Plant	50	50			Geraniaceae				
1368	3	G.purp.	iPlant (Plant Identif	Geranium purpureum	Herb	Plant	0	0			Flowers				
1369	4	G.purp.	iPlant (Plant Identif	Geranium purpureum	Herb	Plant	-5	-5			Lobelias				1
1370	5	G.purp.	iPlant (Plant Identif	Geranium purpureum	Herb	Plant	50	50			Cranesbill				
1371	1	A.diandra	iPlant (Plant Identif	Anisantha diandra	Monocot	Flower	-5	-5	3		Pond pine				1
1372	2	A.diandra	iPlant (Plant Identif	Anisantha diandra	Monocot	Flower	10	10			Sedges				1
1373	3	A.diandra	iPlant (Plant Identif	Anisantha diandra	Monocot	Flower	-5	-5			Pond Pine				1
1374	4	A.diandra	iPlant (Plant Identif	Anisantha diandra	Monocot	Flower	-5	-5			Houseplant				1

1375	5	A.diandra	iPlant (Plant Identif	Anisantha diandra	Monocot	Flower	10	10			Sedges		1
1376	1	S.arv.-lf	iPlant (Plant Identif	Spergula arvensis	Herb	Leaf	0	0	2		none		
1377	2	S.arv.-lf	iPlant (Plant Identif	Spergula arvensis	Herb	Leaf	0	0			none		
1378	3	S.arv.-lf	iPlant (Plant Identif	Spergula arvensis	Herb	Leaf	0	0			none		
1379	4	S.arv.-lf	iPlant (Plant Identif	Spergula arvensis	Herb	Leaf	0	0			none		
1380	5	S.arv.-lf	iPlant (Plant Identif	Spergula arvensis	Herb	Leaf	0	0			Sea plantain		1
1381	1	S.arv.-fl	iPlant (Plant Identif	Spergula arvensis	Herb	Flower	0	0	4		Wood anenome		1
1382	2	S.arv.-fl	iPlant (Plant Identif	Spergula arvensis	Herb	Flower	0	0			Borages		1
1383	3	S.arv.-fl	iPlant (Plant Identif	Spergula arvensis	Herb	Flower	0	0			Lilies		1
1384	4	S.arv.-fl	iPlant (Plant Identif	Spergula arvensis	Herb	Flower	0	0			Jasmine		1
1385	5	S.arv.-fl	iPlant (Plant Identif	Spergula arvensis	Herb	Flower	0	0			Lilies		1
1386	1	S.arv.-pl	iPlant (Plant Identif	Spergula arvensis	Herb	Plant	-5	-5	3		Evergreen rose		1
1387	2	S.arv.-pl	iPlant (Plant Identif	Spergula arvensis	Herb	Plant	-5	-5			Crown vetch		1
1388	3	S.arv.-pl	iPlant (Plant Identif	Spergula arvensis	Herb	Plant	0	0			Wood anenome		1
1389	4	S.arv.-pl	iPlant (Plant Identif	Spergula arvensis	Herb	Plant	-5	-5			Evergreen rose		1
1390	5	S.arv.-pl	iPlant (Plant Identif	Spergula arvensis	Herb	Plant	-5	-5			Evergreen rose		1
1391	1	S.retic.	iPlant (Plant Identif	Salix reticulata	Woody	Leaf	-5	-5	3		Dossinia(orchid)		1
1392	2	S.retic.	iPlant (Plant Identif	Salix reticulata	Woody	Leaf	-5	-5			Nervillia(Asparagales)		1
1393	3	S.retic.	iPlant (Plant Identif	Salix reticulata	Woody	Leaf	-5	-5			Nervillia(Asparagales)		1
1394	4	S.retic.	iPlant (Plant Identif	Salix reticulata	Woody	Leaf	-5	-5			Aquatic plant		1
1395	5	S.retic.	iPlant (Plant Identif	Salix reticulata	Woody	Leaf	-5	-5			Dossinia		1
1396	1	A.sylv-fl	iPlant (Plant Identif	Angelica sylvestris	Herb	Flower	0	0	2		Valerian		1
1397	2	A.sylv-fl	iPlant (Plant Identif	Angelica sylvestris	Herb	Flower	0	0			Valerian		1
1398	3	A.sylv-fl	iPlant (Plant Identif	Angelica sylvestris	Herb	Flower	0	0			Valerian		1
1399	4	A.sylv-fl	iPlant (Plant Identif	Angelica sylvestris	Herb	Flower	0	0			Valerian		1
1400	5	A.sylv-fl	iPlant (Plant Identif	Angelica sylvestris	Herb	Flower	0	0			Milkweed		1
1401	1	A.sylv-lf	iPlant (Plant Identif	Angelica sylvestris	Herb	Leaf	-5	-5	1		Alsike clover		1
1402	2	A.sylv-lf	iPlant (Plant Identif	Angelica sylvestris	Herb	Leaf	-5	-5			Alsike clover		1
1403	3	A.sylv-lf	iPlant (Plant Identif	Angelica sylvestris	Herb	Leaf	-5	-5			Alsike clover		1
1404	4	A.sylv-lf	iPlant (Plant Identif	Angelica sylvestris	Herb	Leaf	-5	-5			Alsike clover		1
1405	5	A.sylv-lf	iPlant (Plant Identif	Angelica sylvestris	Herb	Leaf	-5	-5			Alsike clover		1
1406	1	A.syl-pl	iPlant (Plant Identif	Angelica sylvestris	Herb	Plant	50	50	3.2		Wild celery		1
1407	2	A.syl-pl	iPlant (Plant Identif	Angelica sylvestris	Herb	Plant	50	50			Wild celery		1
1408	3	A.syl-pl	iPlant (Plant Identif	Angelica sylvestris	Herb	Plant	50	50			Cowparsley		
1409	4	A.syl-pl	iPlant (Plant Identif	Angelica sylvestris	Herb	Plant	0	0			Milkweed		1
1410	5	A.syl-pl	iPlant (Plant Identif	Angelica sylvestris	Herb	Plant	0	0			Flowers		
1411	1	A.sylv-fr	iPlant (Plant Identif	Angelica sylvestris	Herb	Fruit	-5	-5	2		Floral design		1
1412	2	A.sylv-fr	iPlant (Plant Identif	Angelica sylvestris	Herb	Fruit	-5	-5			Bud		1
1413	3	A.sylv-fr	iPlant (Plant Identif	Angelica sylvestris	Herb	Fruit	-5	-5			Bud		1
1414	4	A.sylv-fr	iPlant (Plant Identif	Angelica sylvestris	Herb	Fruit	-5	-5			Bud		1
1415	5	A.sylv-fr	iPlant (Plant Identif	Angelica sylvestris	Herb	Fruit	-5	-5			Bud		1
1416	1	A.caucal.-fl	iPlant (Plant Identif	Anthriscus caucalis	Herb	Flower	-5	-5	2.4		Tanacetum		1
1417	2	A.caucal.-fl	iPlant (Plant Identif	Anthriscus caucalis	Herb	Flower	-5	-5			Symphotrichum		1
1418	3	A.caucal.-fl	iPlant (Plant Identif	Anthriscus caucalis	Herb	Flower	-5	-5			Tansey		1
1419	4	A.caucal.-fl	iPlant (Plant Identif	Anthriscus caucalis	Herb	Flower	0	0			Allyssum		1
1420	5	A.caucal.-fl	iPlant (Plant Identif	Anthriscus caucalis	Herb	Flower	-5	-5			Phlox		1
1421	1	A.caucal.-pl	iPlant (Plant Identif	Anthriscus caucalis	Herb	Plant	-5	-5	4		Flowers		
1422	2	A.caucal.-pl	iPlant (Plant Identif	Anthriscus caucalis	Herb	Plant	-5	-5			Eyebright		1
1423	3	A.caucal.-pl	iPlant (Plant Identif	Anthriscus caucalis	Herb	Plant	-5	-5			Tree of heaven		1
1424	4	A.caucal.-pl	iPlant (Plant Identif	Anthriscus caucalis	Herb	Plant	-5	-5			Borages		1
1425	5	A.caucal.-pl	iPlant (Plant Identif	Anthriscus caucalis	Herb	Plant	-5	-5			Eyebright		1
1426	1	A.caucal-lf	iPlant (Plant Identif	Anthriscus caucalis	Herb	Leaf	-5	-5	2		Houseplant		1
1427	2	A.caucal-lf	iPlant (Plant Identif	Anthriscus caucalis	Herb	Leaf	-5	-5			Houseplant		1
1428	3	A.caucal-lf	iPlant (Plant Identif	Anthriscus caucalis	Herb	Leaf	-5	-5			Fern		1
1429	4	A.caucal-lf	iPlant (Plant Identif	Anthriscus caucalis	Herb	Leaf	-5	-5			Houseplant		1
1430	5	A.caucal-lf	iPlant (Plant Identif	Anthriscus caucalis	Herb	Leaf	-5	-5			Fern		1
1431	1	H.elod-Fl	iPlant (Plant Identif	Hypericum elodes	Herb	Flower	0	0	3		none		
1432	2	H.elod-Fl	iPlant (Plant Identif	Hypericum elodes	Herb	Flower	0	0			Eyebright		1
1433	3	H.elod-Fl	iPlant (Plant Identif	Hypericum elodes	Herb	Flower	0	0			none		
1434	4	H.elod-Fl	iPlant (Plant Identif	Hypericum elodes	Herb	Flower	-5	-5			Cabbage white		1
1435	5	H.elod-Fl	iPlant (Plant Identif	Hypericum elodes	Herb	Flower	0	0			none		
1436	1	H.elod-lf	iPlant (Plant Identif	Hypericum elodes	Herb	Leaf	0	0	3		none		
1437	2	H.elod-lf	iPlant (Plant Identif	Hypericum elodes	Herb	Leaf	-5	-5			Cactus		1
1438	3	H.elod-lf	iPlant (Plant Identif	Hypericum elodes	Herb	Leaf	-5	-5			Cactus		1
1439	4	H.elod-lf	iPlant (Plant Identif	Hypericum elodes	Herb	Leaf	-5	-5			Succulent		1
1440	5	H.elod-lf	iPlant (Plant Identif	Hypericum elodes	Herb	Leaf	0	0			none		
1441	1	H.elod-pl	iPlant (Plant Identif	Hypericum elodes	Herb	Plant	-5	-5	5		Borages		1
1442	2	H.elod-pl	iPlant (Plant Identif	Hypericum elodes	Herb	Plant	-5	-5			Medick		1
1443	3	H.elod-pl	iPlant (Plant Identif	Hypericum elodes	Herb	Plant	-5	-5			Dietary fibre		1
1444	4	H.elod-pl	iPlant (Plant Identif	Hypericum elodes	Herb	Plant	-5	-5			Stonecrops		1
1445	5	H.elod-pl	iPlant (Plant Identif	Hypericum elodes	Herb	Plant	-5	-5			Fern		1
1446	1	C.remot-fl1	iPlant (Plant Identif	Carex remota	Monocot	Flower	50	50	1		Sedge		
1447	2	C.remot-fl1	iPlant (Plant Identif	Carex remota	Monocot	Flower	50	50			Sedge		
1448	3	C.remot-fl1	iPlant (Plant Identif	Carex remota	Monocot	Flower	50	50			Sedge		
1449	4	C.remot-fl1	iPlant (Plant Identif	Carex remota	Monocot	Flower	50	50			Sedge		
1450	5	C.remot-fl1	iPlant (Plant Identif	Carex remota	Monocot	Flower	50	50			Sedge		
1451	1	C.remot-pl	iPlant (Plant Identif	Carex remota	Monocot	Plant	50	50	4		Sedge		
1452	2	C.remot-pl	iPlant (Plant Identif	Carex remota	Monocot	Plant	-5	-5			Bromeliad		1
1453	3	C.remot-pl	iPlant (Plant Identif	Carex remota	Monocot	Plant	0	0			none		
1454	4	C.remot-pl	iPlant (Plant Identif	Carex remota	Monocot	Plant	10	10			Triticale		1
1455	5	C.remot-pl	iPlant (Plant Identif	Carex remota	Monocot	Plant	50	50			Sedge		
1456	1	C.remot-fl2	iPlant (Plant Identif	Carex remota	Monocot	Flower	10	10	4		Sweetgrass		1
1457	2	C.remot-fl2	iPlant (Plant Identif	Carex remota	Monocot	Flower	0	0			herbaceous		
1458	3	C.remot-fl2	iPlant (Plant Identif	Carex remota	Monocot	Flower	10	10			Sweetgrass		1
1459	4	C.remot-fl2	iPlant (Plant Identif	Carex remota	Monocot	Flower	-5	-5			Yarn		1
1460	5	C.remot-fl2	iPlant (Plant Identif	Carex remota	Monocot	Flower	0	0			none		
1461	1	Q.rob-lf	iPlant (Plant Identif	Quercus robur	Woody	Leaf	80	80	1.2		Q.macrocarpa		
1462	2	Q.rob-lf	iPlant (Plant Identif	Quercus robur	Woody	Leaf	80	80			Q.macrocarpa		
1463	3	Q.rob-lf	iPlant (Plant Identif	Quercus robur	Woody	Leaf	80	80			Q.macrocarpa		
1464	4	Q.rob-lf	iPlant (Plant Identif	Quercus robur	Woody	Leaf	80	80			Q.gambelii		
1465	5	Q.rob-lf	iPlant (Plant Identif	Quercus robur	Woody	Leaf	80	80			Q.macrocarpa		
1466	1	Q.rob-fr	iPlant (Plant Identif	Quercus robur	Woody	Fruit	80	80	2.2		Quercus		
1467	2	Q.rob-fr	iPlant (Plant Identif	Quercus robur	Woody	Fruit	100	100			Q.robur		
1468	3	Q.rob-fr	iPlant (Plant Identif	Quercus robur	Woody	Fruit	80	80			Quercus		
1469	4	Q.rob-fr	iPlant (Plant Identif	Quercus robur	Woody	Fruit	80	80			Quercus		
1470	5	Q.rob-fr	iPlant (Plant Identif	Quercus robur	Woody	Fruit	0	0			no plant		
1471	1	Q.rob-pl	iPlant (Plant Identif	Quercus robur	Woody	Plant	-5	-5	2.6	spruce/evergreen etc one family(isH0	Black spruce		1
1472	2	Q.rob-pl	iPlant (Plant Identif	Quercus robur	Woody	Plant	-5	-5			Evergreen		1
1473	3	Q.rob-pl	iPlant (Plant Identif	Quercus robur	Woody	Plant	-5	-5			Black spruce		1
1474	4	Q.rob-pl	iPlant (Plant Identif	Quercus robur	Woody	Plant	-5	-5			Cupressaceae		1
1475	5	Q.rob-pl	iPlant (Plant Identif	Quercus robur	Woody	Plant	-5	-5			Larch		1
1476	1	E.nigr-fl	iPlant (Plant Identif	Empetrum nigrum	Woody	Flower	0	0	3		Bud		
1477	2	E.nigr-fl	iPlant (Plant Identif	Empetrum nigrum	Woody	Flower	0	0			no plant		
1478	3	E.nigr-fl	iPlant (Plant Identif	Empetrum nigrum	Woody	Flower	-5	-5			Orchids		1
1479	4	E.nigr-fl	iPlant (Plant Identif	Empetrum nigrum	Woody	Flower	0	0			no plants		
1480	5	E.nigr-fl	iPlant (Plant Identif	Empetrum nigrum	Woody	Flower	0	0			no plants		
1481	1	E.nigr-fr	iPlant (Plant Identif	Empetrum nigrum	Woody	Fruit	10	10	3		Juniper berry		1
1482	2	E.nigr-fr	iPlant (Plant Identif	Empetrum nigrum	Woody	Fruit	0	0			Sugar		1
1483	3	E.nigr-fr	iPlant (Plant Identif	Empetrum nigrum	Woody	Fruit	50	50			Cyanococcus	Heathers	1
1484	4	E.nigr-fr	iPlant (Plant Identif	Empetrum nigrum	Woody	Fruit	10	10			Juniper berry		1
1485	5	E.nigr-fr	iPlant (Plant Identif	Empetrum nigrum	Woody	Fruit	10	10			Juniper berry		1
1486	1	A.pseudo	iPlant (Plant Identif	Acer pseudoplatanus	Woody	Leaf	20	20	3		Vitis		1
1487	2	A.pseudo	iPlant (Plant Identif	Acer pseudoplatanus	Woody	Leaf	80	80			Acer glabrum		
1488	3	A.pseudo	iPlant (Plant Identif	Acer pseudoplatanus	Woody	Leaf	20	20			Vitis		1
1489	4	A.pseudo	iPlant (Plant Identif	Acer pseudoplatanus	Woody	Leaf	80	80			Maple leaf		
1490	5	A.pseudo	iPlant (Plant Identif	Acer pseudoplatanus	Woody	Leaf	80	80			Maple leaf		
1491	1	C.pauci	iPlant (Plant Identif	Carex pauciflora	Monocot	Flower	0	0	2		none		
1492	2	C.pauci	iPlant (Plant Identif	Carex pauciflora	Monocot	Flower	0	0			none		
1493	3	C.pauci	iPlant (Plant Identif	Carex pauciflora	Monocot	Flower	0	0			none		
1494	4	C.pauci	iPlant (Plant Identif	Carex pauciflora	Monocot	Flower	50	50			Sedges		
1495	5	C.pauci	iPlant (Plant Identif	Carex pauciflora	Monocot	Flower	50	50			Sedges		
1496	1	C.Fuscus	iPlant (Plant Identif	Cyperus fuscus	Monocot	Plant	-5	-5	2		Wolf spider		1
1497	2	C.Fuscus	iPlant (Plant Identif	Cyperus fuscus	Monocot	Plant	50	50			Sedges		

1498	3	C.Fuscus	iPlant (Plant Identif	Cyperus fuscus	Monocot	Plant	-5	-5		Wolf spider				1
1499	4	C.Fuscus	iPlant (Plant Identif	Cyperus fuscus	Monocot	Plant	50	50		Sedges				
1500	5	C.Fuscus	iPlant (Plant Identif	Cyperus fuscus	Monocot	Plant	50	50		Sedges				
1501	1	T.marit	iPlant (Plant Identif	Triglochin maritima	Monocot	Plant	0	0	4	Hierochloe				1
1502	2	T.marit	iPlant (Plant Identif	Triglochin maritima	Monocot	Plant	0	0		none				
1503	3	T.marit	iPlant (Plant Identif	Triglochin maritima	Monocot	Plant	0	0		Flowers				
1504	4	T.marit	iPlant (Plant Identif	Triglochin maritima	Monocot	Plant	0	0		none				
1505	5	T.marit	iPlant (Plant Identif	Triglochin maritima	Monocot	Plant	0	0		Sedges				
1506	1	Delairia	iPlant (Plant Identif	Delairea odorata	Herb	Plant	5	5	4	Acer saccharum				1
1507	2	Delairia	iPlant (Plant Identif	Delairea odorata	Herb	Plant	10	10		Grape vine				1
1508	3	Delairia	iPlant (Plant Identif	Delairea odorata	Herb	Plant	-5	-5		Dietary fibre				1
1509	4	Delairia	iPlant (Plant Identif	Delairea odorata	Herb	Plant	80	80		Acer saccharum				1
1510	5	Delairia	iPlant (Plant Identif	Delairea odorata	Herb	Plant	-5	-5		Milkweed				1
1511	1	E.bonar	iPlant (Plant Identif	Erigeron bonariensis	Herb	Flower	-5	-5	2.4	Teasels				1
1512	2	E.bonar	iPlant (Plant Identif	Erigeron bonariensis	Herb	Flower	50	50		Hawkweed				1
1513	3	E.bonar	iPlant (Plant Identif	Erigeron bonariensis	Herb	Flower	50	50		Field marigold				1
1514	4	E.bonar	iPlant (Plant Identif	Erigeron bonariensis	Herb	Flower	50	50		Symphotrichum				1
1515	5	E.bonar	iPlant (Plant Identif	Erigeron bonariensis	Herb	Flower	50	50		Hawkweed				1
1516	1	M.mosch	iPlant (Plant Identif	Malva moschata	Herb	Flower	5	5	2	Crane's-bill				1
1517	2	M.mosch	iPlant (Plant Identif	Malva moschata	Herb	Flower	5	5		Crane's-bill				1
1518	3	M.mosch	iPlant (Plant Identif	Malva moschata	Herb	Flower	50	50		Blue hibiscus				1
1519	4	M.mosch	iPlant (Plant Identif	Malva moschata	Herb	Flower	0	0		Crane's-bill				1
1520	5	M.mosch	iPlant (Plant Identif	Malva moschata	Herb	Flower	50	50		Blue hibiscus				1
1521	1	P.lutea	PlantSnap	Psuedofumaria lutea	Herb	Plant	95	95	1.4	Corydalis fruticosa	P.lutea			
1522	2	P.lutea	PlantSnap	Psuedofumaria lutea	Herb	Plant	100	100		P.lutea				
1523	3	P.lutea	PlantSnap	Psuedofumaria lutea	Herb	Plant	75	75		Dactylicapnos	P.lutea			
1524	4	P.lutea	PlantSnap	Psuedofumaria lutea	Herb	Plant	100	100		P.lutea				
1525	5	P.lutea	PlantSnap	Psuedofumaria lutea	Herb	Plant	100	100		P.lutea				
1526	1	D.purp.	PlantSnap	Digitalis purpurea	Herb	Leaf	100	100	1	D.purpurea				
1527	2	D.purp.	PlantSnap	Digitalis purpurea	Herb	Leaf	100	100		D.purpurea				
1528	3	D.purp.	PlantSnap	Digitalis purpurea	Herb	Leaf	100	100		D.purpurea				
1529	4	D.purp.	PlantSnap	Digitalis purpurea	Herb	Leaf	100	100		D.purpurea				
1530	5	D.purp.	PlantSnap	Digitalis purpurea	Herb	Leaf	100	100		D.purpurea				
1531	1	S.coron.	PlantSnap	Silene coronaria	Herb	Leaf	10	10	3	Verbascum thapsus				1
1532	2	S.coron.	PlantSnap	Silene coronaria	Herb	Leaf	10	10		Verbascum thapsus				1
1533	3	S.coron.	PlantSnap	Silene coronaria	Herb	Leaf	10	10		Verbascum thapsus				1
1534	4	S.coron.	PlantSnap	Silene coronaria	Herb	Leaf	-5	-5		Amorphophallus				1
1535	5	S.coron.	PlantSnap	Silene coronaria	Herb	Leaf	-5	-5		Cotinus	White sage			1
1536	1	C.off.	PlantSnap	Calendula officinalis	Herb	Plant	-5	-5	3.2	Lachenalia	Monarch fern			1
1537	2	C.off.	PlantSnap	Calendula officinalis	Herb	Plant	80	80		Glebionis				
1538	3	C.off.	PlantSnap	Calendula officinalis	Herb	Plant	92	92		Field marigold	Calendula	Clover	C.officinalis	
1539	4	C.off.	PlantSnap	Calendula officinalis	Herb	Plant	90	90		Calendula suffruticosa				
1540	5	C.off.	PlantSnap	Calendula officinalis	Herb	Plant	90	90		Calendula suffruticosa				
1541	1	M.erecta	PlantSnap	Moenchia erecta	Herb	Flower	-5	-5	4.2	Subterranean clover	Waterlily	Vetch	Chickweed	1
1542	2	M.erecta	PlantSnap	Moenchia erecta	Herb	Flower	-5	-5		Yellow vetch	Enephalartos			1
1543	3	M.erecta	PlantSnap	Moenchia erecta	Herb	Flower	-5	-5		Banana	Lily			1
1544	4	M.erecta	PlantSnap	Moenchia erecta	Herb	Flower	-5	-5		Amorphophallus				1
1545	5	M.erecta	PlantSnap	Moenchia erecta	Herb	Flower	0	0		Anemone	Euphorbia			1
1546	1	S.verna	PlantSnap	Sabulina verna	Herb	Plant	-5	-5	4.2	Lycopodium	Petrosedum			1
1547	2	S.verna	PlantSnap	Sabulina verna	Herb	Plant	-5	23		Gentiana acaulis	Cerastium arcticum			1
1548	3	S.verna	PlantSnap	Sabulina verna	Herb	Plant	50	50		Arenaria paludicola				1
1549	4	S.verna	PlantSnap	Sabulina verna	Herb	Plant	50	50		Cheleria arctica				1
1550	5	S.verna	PlantSnap	Sabulina verna	Herb	Plant	0	25		Parnassia	Stitchwort			1
1551	1	E.marit.	PlantSnap	Erodium maritimum	Herb	Leaf	-5	-5	5	Marchantia	Asplenium			1
1552	2	E.marit.	PlantSnap	Erodium maritimum	Herb	Leaf	-5	-5		Salvia argentea	Aloe			1
1553	3	E.marit.	PlantSnap	Erodium maritimum	Herb	Leaf	-5	-5		Spleenwort	Spleenwort			1
1554	4	E.marit.	PlantSnap	Erodium maritimum	Herb	Leaf	-5	-5		Kidney weed	Berula	Oxalis		1
1555	5	E.marit.	PlantSnap	Erodium maritimum	Herb	Leaf	-5	-5		Teasels	Tiarella			1
1556	1	G.purp.	PlantSnap	Geranium purpureum	Herb	Plant	90	90	2.4	G.robertianum				
1557	2	G.purp.	PlantSnap	Geranium purpureum	Herb	Plant	50	58		Erodium circuitarum	Malva	Geranium		1
1558	3	G.purp.	PlantSnap	Geranium purpureum	Herb	Plant	0	50		Malva sylvestris	Little robin			1
1559	4	G.purp.	PlantSnap	Geranium purpureum	Herb	Plant	80	80		G.carolinianum	Malva sylvestris	G.dissectum		
1560	5	G.purp.	PlantSnap	Geranium purpureum	Herb	Plant	80	83		G.carolinianum	Malva sylvestris	Little robin		
1561	1	A.diandra	PlantSnap	Anisantha diandra	Monocot	Flower	50	78	1.8	Elymus diversiglumis	Bromus tectorum	B.diandrus		1
1562	2	A.diandra	PlantSnap	Anisantha diandra	Monocot	Flower	90	93		B.tectorum	Callipedium	B.diandrus		
1563	3	A.diandra	PlantSnap	Anisantha diandra	Monocot	Flower	50	57		Elymus	Hystrix	E.diversiglumis	B.tectorum	1
1564	4	A.diandra	PlantSnap	Anisantha diandra	Monocot	Flower	50	70		Elymus	B.tectorum			1
1565	5	A.diandra	PlantSnap	Anisantha diandra	Monocot	Flower	50	78		Callipedium	B.ramosus	B.diandrus		1
1566	1	S.arv.-lf	PlantSnap	Spergula arvensis	Herb	Leaf	0	0	3.2	Allium sativum	Allium			1
1567	2	S.arv.-lf	PlantSnap	Spergula arvensis	Herb	Leaf	0	0		Allium sativum	Umbrella pine			
1568	3	S.arv.-lf	PlantSnap	Spergula arvensis	Herb	Leaf	0	0		Allium vineale	Allium sativum			1
1569	4	S.arv.-lf	PlantSnap	Spergula arvensis	Herb	Leaf	-5	-5		Banksia	Plantago			1
1570	5	S.arv.-lf	PlantSnap	Spergula arvensis	Herb	Leaf	0	0		Equisetum arvense	Equisetum hyemale			1
1571	1	S.arv.-fl	PlantSnap	Spergula arvensis	Herb	Flower	0	0	3.2	Drosera villosa				1
1572	2	S.arv.-fl	PlantSnap	Spergula arvensis	Herb	Flower	100	100		Corn spurrey				
1573	3	S.arv.-fl	PlantSnap	Spergula arvensis	Herb	Flower	100	100		Corn spurrey				
1574	4	S.arv.-fl	PlantSnap	Spergula arvensis	Herb	Flower	0	0		Galanthus plicatus	Galanthus	Parnassia		1
1575	5	S.arv.-fl	PlantSnap	Spergula arvensis	Herb	Flower	0	50		Drosera linearis	Corn spurrey			1
1576	1	S.arv.-pl	PlantSnap	Spergula arvensis	Herb	Plant	50	50	1.4	Cerastium tomentosum	Cerastium alpinum			1
1577	2	S.arv.-pl	PlantSnap	Spergula arvensis	Herb	Plant	50	50		Cerastium arvense	Cerastium bannaticum			1
1578	3	S.arv.-pl	PlantSnap	Spergula arvensis	Herb	Plant	90	90		Spergula villosa				
1579	4	S.arv.-pl	PlantSnap	Spergula arvensis	Herb	Plant	100	100		Spergula arvensis				
1580	5	S.arv.-pl	PlantSnap	Spergula arvensis	Herb	Plant	50	50		Cerastium tomentosum	Cerastium arvensis			1
1581	1	S.retic.	PlantSnap	Salix reticulata	Woody	Leaf	100	100	2	Net-leaved willow				
1582	2	S.retic.	PlantSnap	Salix reticulata	Woody	Leaf	100	100		Net-leaved willow				
1583	3	S.retic.	PlantSnap	Salix reticulata	Woody	Leaf	0	25		Plectranthus cilliatus	Brunnera	Net-leaved willow		1
1584	4	S.retic.	PlantSnap	Salix reticulata	Woody	Leaf	100	100		Net-leaved willow				
1585	5	S.retic.	PlantSnap	Salix reticulata	Woody	Leaf	-5	-5		Plectranthus cilliatus	Phlomis	Brunnera		1
1586	1	A.sylv-fl	PlantSnap	Angelica sylvestris	Herb	Flower	-5	3	2	Allium pskemenske	Allium stitiatum	A.ampeloprasum	#6-A.sylvestris	1
1587	2	A.sylv-fl	PlantSnap	Angelica sylvestris	Herb	Flower	100	100		A.sylvestris				
1588	3	A.sylv-fl	PlantSnap	Angelica sylvestris	Herb	Flower	100	100		A.sylvestris				
1589	4	A.sylv-fl	PlantSnap	Angelica sylvestris	Herb	Flower	100	100		A.sylvestris				
1590	5	A.sylv-fl	PlantSnap	Angelica sylvestris	Herb	Flower	100	100		A.sylvestris				
1591	1	A.sylv-lf	PlantSnap	Angelica sylvestris	Herb	Leaf	80	80	2.2	A.sinensis				
1592	2	A.sylv-lf	PlantSnap	Angelica sylvestris	Herb	Leaf	0	50		Rhus copalinum	A.sylvestris			1
1593	3	A.sylv-lf	PlantSnap	Angelica sylvestris	Herb	Leaf	100	100		A.sylvestris				
1594	4	A.sylv-lf	PlantSnap	Angelica sylvestris	Herb	Leaf	100	100		A.sylvestris				
1595	5	A.sylv-lf	PlantSnap	Angelica sylvestris	Herb	Leaf	0	0		Rhus copalinum	American liquorice			1
1596	1	A.syl-pl	PlantSnap	Angelica sylvestris	Herb	Plant	100	100	1	A.sylvestris				
1597	2	A.syl-pl	PlantSnap	Angelica sylvestris	Herb	Plant	100	100		A.sylvestris				
1598	3	A.syl-pl	PlantSnap	Angelica sylvestris	Herb	Plant	100	100		A.sylvestris				
1599	4	A.syl-pl	PlantSnap	Angelica sylvestris	Herb	Plant	100	100		A.sylvestris				
1600	5	A.syl-pl	PlantSnap	Angelica sylvestris	Herb	Plant	100	100		A.sylvestris				
1601	1	A.sylv-fr	PlantSnap	Angelica sylvestris	Herb	Fruit	-5	-5	4	Kalanchoe	Rheum	Asclepias		1
1602	2	A.sylv-fr	PlantSnap	Angelica sylvestris	Herb	Fruit	-5	-5		Aeonium	Kalanchoe	Senecio		1
1603	3	A.sylv-fr	PlantSnap	Angelica sylvestris	Herb	Fruit	-5	-5		Senecio	Euphorbia			1
1604	4	A.sylv-fr	PlantSnap	Angelica sylvestris	Herb	Fruit	-5	-5		Petasites hybridus	Allium			1
1605	5	A.sylv-fr	PlantSnap	Angelica sylvestris	Herb	Fruit	-5	-5		Kalanchoe	Allium			1
1606	1	A.caucal.-fl	PlantSnap	Anthriscus caucalis	Herb	Flower	-5	-5	5	Pulsatilla occidentale	Poterium			1
1607	2	A.caucal.-fl	PlantSnap	Anthriscus caucalis	Herb	Flower	-5	-5		Equisetum variegatum	Plantago			1
1608	3	A.caucal.-fl	PlantSnap	Anthriscus caucalis	Herb	Flower	-5	-5		Cleistocactus	other Cacti			1
1609	4	A.caucal.-fl	PlantSnap	Anthriscus caucalis	Herb	Flower	0	0		Boechera fendleri	Abronia turbinata			1
1610	5	A.caucal.-fl	PlantSnap	Anthriscus caucalis	Herb	Flower	0	0		Draba muralis	Lappula occidentalis			1
1611	1	A.caucal.-pl	PlantSnap	Anthriscus caucalis	Herb	Plant	13	13	3.2	Achillea millefolium	Achillea	Scandix pecten-veneris		1
1612	2	A.caucal.-pl	PlantSnap	Anthriscus caucalis	Herb	Plant	0	0		Alisma plantago-aquatica	Hottonia	Euphorbia		1
1613	3	A.caucal.-pl	PlantSnap	Anthriscus caucalis	Herb	Plant	0	0		Saxifraga paniculata	Saxifraga			1
1614	4	A.caucal.-pl	PlantSnap	Anthriscus caucalis	Herb	Plant	0	0		Saxifraga paniculata	Anemone	Hottonia		1
1615	5	A.caucal.-pl	PlantSnap	Anthriscus caucalis	Herb	Plant	0	0		Saxifraga	Achillea			1

1616	1	A.caucal-lf	PlantSnap	Anthriscus caucalis	Herb	Leaf	0	40	3		Tanacetum	A.sylvestris			1
1617	2	A.caucal-lf	PlantSnap	Anthriscus caucalis	Herb	Leaf	-5	-5			Amorphophallus	Nephrolepis			1
1618	3	A.caucal-lf	PlantSnap	Anthriscus caucalis	Herb	Leaf	-5	-5			Amorphophallus				1
1619	4	A.caucal-lf	PlantSnap	Anthriscus caucalis	Herb	Leaf	50	65			Conium maculatum	A.sylvestris			1
1620	5	A.caucal-lf	PlantSnap	Anthriscus caucalis	Herb	Leaf	-5	-5			Amorphophallus				1
1621	1	H.elod-Fl	PlantSnap	Hypericum elodes	Herb	Flower	-5	-5			Asclepias	Luffa			1
1622	2	H.elod-Fl	PlantSnap	Hypericum elodes	Herb	Flower	-5	-5	4		Asclepias	Balota			1
1623	3	H.elod-Fl	PlantSnap	Hypericum elodes	Herb	Flower	-5	-5			Luffa	Xerophyllum			1
1624	4	H.elod-Fl	PlantSnap	Hypericum elodes	Herb	Flower	-5	-5			various fungi				1
1625	5	H.elod-Fl	PlantSnap	Hypericum elodes	Herb	Flower	-5	-5			Asclepias	Xerophyllum			1
1626	1	H.elod-lf	PlantSnap	Hypericum elodes	Herb	Leaf	-5	3	5		Tradescantia	Stachys byzantina	Cotton cactus		1
1627	2	H.elod-lf	PlantSnap	Hypericum elodes	Herb	Leaf	-5	3			Silver cactus	Stachys byzantina			1
1628	3	H.elod-lf	PlantSnap	Hypericum elodes	Herb	Leaf	-5	-5			Asclepias pod	Clematis			1
1629	4	H.elod-lf	PlantSnap	Hypericum elodes	Herb	Leaf	-5	-5			Victoria amazonica	Cactus			1
1630	5	H.elod-lf	PlantSnap	Hypericum elodes	Herb	Leaf	0	5			Teucrium	Verascum pulverentum			1
1631	1	H.elod-pl	PlantSnap	Hypericum elodes	Herb	Plant	100	100	4		H.elodes				
1632	2	H.elod-pl	PlantSnap	Hypericum elodes	Herb	Plant	100	100			H.elodes				
1633	3	H.elod-pl	PlantSnap	Hypericum elodes	Herb	Plant	-5	-5			Eutrema	Opuntia			1
1634	4	H.elod-pl	PlantSnap	Hypericum elodes	Herb	Plant	-5	3		Asarina=10	Clematis	Asarina			1
1635	5	H.elod-pl	PlantSnap	Hypericum elodes	Herb	Plant	10	10			Salvia argentea				1
1636	1	C.remot-fl1	PlantSnap	Carex remota	Monocot	Flower	80	80	2.8		C.atherodes				
1637	2	C.remot-fl1	PlantSnap	Carex remota	Monocot	Flower	80	80			C.brunnescens	C.pilulifera			
1638	3	C.remot-fl1	PlantSnap	Carex remota	Monocot	Flower	-5	3			Chinese beech	Juncus longistylis			1
1639	4	C.remot-fl1	PlantSnap	Carex remota	Monocot	Flower	80	80			C.bauxbaumii	C.pilulifera			
1640	5	C.remot-fl1	PlantSnap	Carex remota	Monocot	Flower	80	80			C.cherokensis	C.pilulifera			
1641	1	C.remot-pl	PlantSnap	Carex remota	Monocot	Plant	10	27	2.2		Calamagrostis	Calamagrostis	C.rosea		1
1642	2	C.remot-pl	PlantSnap	Carex remota	Monocot	Plant	10	27			Calamagrostis	Calamagrostis	C.rubescens		1
1643	3	C.remot-pl	PlantSnap	Carex remota	Monocot	Plant	80	80			C.rubescens	C.arundinacea			
1644	4	C.remot-pl	PlantSnap	Carex remota	Monocot	Plant	10	10			Miscanthus	Calamagrostis	Sporobolus		1
1645	5	C.remot-pl	PlantSnap	Carex remota	Monocot	Plant	80	80			C.rubescens	Calamagrostis			
1646	1	C.remot-fl2	PlantSnap	Carex remota	Monocot	Flower	-5	38	3		Banana	Briza			1
1647	2	C.remot-fl2	PlantSnap	Carex remota	Monocot	Flower	10	20			Sporobolus	Buotoloa	Fimbristylis(sedge)		1
1648	3	C.remot-fl2	PlantSnap	Carex remota	Monocot	Flower	-5	-1			Banana	Asplenium	Zea		1
1649	4	C.remot-fl2	PlantSnap	Carex remota	Monocot	Flower	-5	-5			Quesnellia	Boutoloa	Achmeia		1
1650	5	C.remot-fl2	PlantSnap	Carex remota	Monocot	Flower	-5	-1			Musa	Tillandsia	Boutoloa		1
1651	1	Q.rob-lf	PlantSnap	Quercus robur	Woody	Leaf	-5	-5	4		Pitcher plant	Asplenium			1
1652	2	Q.rob-lf	PlantSnap	Quercus robur	Woody	Leaf	-5	-5			Horseradish	Pitcher plant			1
1653	3	Q.rob-lf	PlantSnap	Quercus robur	Woody	Leaf	-5	-5			Harts tongue	Asplenium			1
1654	4	Q.rob-lf	PlantSnap	Quercus robur	Woody	Leaf	80	80			Q.macrophylla				
1655	5	Q.rob-lf	PlantSnap	Quercus robur	Woody	Leaf	-5	-5			Horseradish	Microsorum musifolium			1
1656	1	Q.rob-fr	PlantSnap	Quercus robur	Woody	Fruit	-5	-5	3		Banksia sp	Banksia			1
1657	2	Q.rob-fr	PlantSnap	Quercus robur	Woody	Fruit	80	80			Q.macrocarpa				
1658	3	Q.rob-fr	PlantSnap	Quercus robur	Woody	Fruit	80	80			Q.macrocarpa				
1659	4	Q.rob-fr	PlantSnap	Quercus robur	Woody	Fruit	80	80			Q.macrocarpa				
1660	5	Q.rob-fr	PlantSnap	Quercus robur	Woody	Fruit	-5	18			Ficus	Q.macrocaarpa			1
1661	1	Q.rob-pl	PlantSnap	Quercus robur	Woody	Plant	0	0	3		Fagus sylvatica				1
1662	2	Q.rob-pl	PlantSnap	Quercus robur	Woody	Plant	-5	-5			Buxus	Oak mistletoe			1
1663	3	Q.rob-pl	PlantSnap	Quercus robur	Woody	Plant	-5	-5			Portulacaria	Fagus	Box		1
1664	4	Q.rob-pl	PlantSnap	Quercus robur	Woody	Plant	-5	-5			Box	Fagus	Portulacaria		1
1665	5	Q.rob-pl	PlantSnap	Quercus robur	Woody	Plant	-5	-5			Box	Fagus			1
1666	1	E.nigr-fl	PlantSnap	Empetrum nigrum	Woody	Flower	-5	-5	2		Sundew	Knightia	Potea	Empetrum	1
1667	2	E.nigr-fl	PlantSnap	Empetrum nigrum	Woody	Flower	100	100			E.nigrum				
1668	3	E.nigr-fl	PlantSnap	Empetrum nigrum	Woody	Flower	100	100			E.nigrum				
1669	4	E.nigr-fl	PlantSnap	Empetrum nigrum	Woody	Flower	100	100			E.nigrum				
1670	5	E.nigr-fl	PlantSnap	Empetrum nigrum	Woody	Flower	100	100			E.nigrum				
1671	1	E.nigr-fr	PlantSnap	Empetrum nigrum	Woody	Fruit	100	100	1		E.nigrum				
1672	2	E.nigr-fr	PlantSnap	Empetrum nigrum	Woody	Fruit	100	100			E.nigrum				
1673	3	E.nigr-fr	PlantSnap	Empetrum nigrum	Woody	Fruit	100	100			E.nigrum				
1674	4	E.nigr-fr	PlantSnap	Empetrum nigrum	Woody	Fruit	100	100			E.nigrum				
1675	5	E.nigr-fr	PlantSnap	Empetrum nigrum	Woody	Fruit	100	100			E.nigrum				
1676	1	A.pseudo	PlantSnap	Acer pseudoplatanus	Woody	Leaf	50	50	1.2		Aesculus hippocastaneum				1
1677	2	A.pseudo	PlantSnap	Acer pseudoplatanus	Woody	Leaf	100	100			A.pseudoplatanus				
1678	3	A.pseudo	PlantSnap	Acer pseudoplatanus	Woody	Leaf	100	100			A.pseudoplatanus				
1679	4	A.pseudo	PlantSnap	Acer pseudoplatanus	Woody	Leaf	100	100			A.pseudoplatanus				
1680	5	A.pseudo	PlantSnap	Acer pseudoplatanus	Woody	Leaf	50	75			Aesculus hippocastaneum	A.pseudoplatanus			1
1681	1	C.pauci	PlantSnap	Carex pauciflora	Monocot	Flower	10	10	3.2		Cenchrus americanus	Cactus			1
1682	2	C.pauci	PlantSnap	Carex pauciflora	Monocot	Flower	0	5			Equisetum hyemale	Grass			1
1683	3	C.pauci	PlantSnap	Carex pauciflora	Monocot	Flower	100	100			C.pauciflora	C.microglochin			
1684	4	C.pauci	PlantSnap	Carex pauciflora	Monocot	Flower	10	10			Boutoloa gracilis				1
1685	5	C.pauci	PlantSnap	Carex pauciflora	Monocot	Flower	0	5			Equisetum hyemale	Grass			1
1686	1	C.Fuscus	PlantSnap	Cyperus fuscus	Monocot	Plant	-5	3	4.2		Casuarina	Setaria			1
1687	2	C.Fuscus	PlantSnap	Cyperus fuscus	Monocot	Plant	-5	-5			Pinus	Wollemi			1
1688	3	C.Fuscus	PlantSnap	Cyperus fuscus	Monocot	Plant	50	65			Schoenoplectus acutus	Cyperus sphaerocephalus			1
1689	4	C.Fuscus	PlantSnap	Cyperus fuscus	Monocot	Plant	10	30			Juncus acutus	Gahnia(sedge)			1
1690	5	C.Fuscus	PlantSnap	Cyperus fuscus	Monocot	Plant	80	80			Cyperus				
1691	1	T.marit	PlantSnap	Triglochin maritima	Monocot	Plant	0	0	3		Goodyera repens	Equisetum			1
1692	2	T.marit	PlantSnap	Triglochin maritima	Monocot	Plant	20	20		P.maritma=20	Plantago maritima				1
1693	3	T.marit	PlantSnap	Triglochin maritima	Monocot	Plant	-5	-5			Equisetum arvense	Equisetum telemateia			1
1694	4	T.marit	PlantSnap	Triglochin maritima	Monocot	Plant	20	20			Plantago maritima				1
1695	5	T.marit	PlantSnap	Triglochin maritima	Monocot	Plant	20	20			Plantago maritima				1
1696	1	Delairia	PlantSnap	Delairea odorata	Herb	Plant	-5	-5	3		Mellispermum	Cissus			1
1697	2	Delairia	PlantSnap	Delairea odorata	Herb	Plant	0	0			Hedera nepalensis	Firmiana			1
1698	3	Delairia	PlantSnap	Delairea odorata	Herb	Plant	100	100			Delairea				
1699	4	Delairia	PlantSnap	Delairea odorata	Herb	Plant	-5	-5			Mellispermum	Mallotus			1
1700	5	Delairia	PlantSnap	Delairea odorata	Herb	Plant	100	100			Delariea				
1701	1	E.bonar	PlantSnap	Erigeron bonariensis	Herb	Flower	0	25	2.4		Crassocephalum	Erechtites			1
1702	2	E.bonar	PlantSnap	Erigeron bonariensis	Herb	Flower	100	100			E.bonariensis				
1703	3	E.bonar	PlantSnap	Erigeron bonariensis	Herb	Flower	90	90			E.sumatrensis				
1704	4	E.bonar	PlantSnap	Erigeron bonariensis	Herb	Flower	90	90			E.sumatrensis				
1705	5	E.bonar	PlantSnap	Erigeron bonariensis	Herb	Flower	80	80			E.acer				
1706	1	M.mosch	PlantSnap	Malva moschata	Herb	Flower	80	80	1.2		M.alcea	Alcea biennis			
1707	2	M.mosch	PlantSnap	Malva moschata	Herb	Flower	100	100			M.moschata	M.alcea			
1708	3	M.mosch	PlantSnap	Malva moschata	Herb	Flower	80	90			M.alcea	M.moschata			
1709	4	M.mosch	PlantSnap	Malva moschata	Herb	Flower	80	90			M.alcea	M.moschata			
1710	5	M.mosch	PlantSnap	Malva moschata	Herb	Flower	100	100			M.moschata				
